# HIV/HCV therapy with ledipasvir/sofosbuvir after randomized switch to emtricitabine-tenofovir alafenamide-based single-tablet regimens

**DOI:** 10.1371/journal.pone.0224875

**Published:** 2020-01-29

**Authors:** Gregory D. Huhn, Moti Ramgopal, Mamta K. Jain, Federico Hinestrosa, David M. Asmuth, Jihad Slim, Deborah Goldstein, Shauna Applin, Julie H. Ryu, Shuping Jiang, Stephanie Cox, Moupali Das, Thai Nguyen-Cleary, David Piontkowsky, Bill Guyer, Lorenzo Rossaro, Richard H. Haubrich

**Affiliations:** 1 Ruth M Rothstein CORE Center, Chicago, IL, United States of America; 2 Midway Research Center, Fort Pierce, FL, United States of America; 3 UT Southwestern Medical Center, Dallas, TX, United States of America; 4 Orlando Immunology Center, Orlando, FL, United States of America; 5 University of California Davis, Sacramento, CA, United States of America; 6 Saint Michael's Medical Center, Newark, NJ, United States of America; 7 Whitman-Walker Health, Washington DC, United States of America; 8 Community Health Care, Tacoma, WA, United States of America; 9 Gilead Sciences, Inc., Foster City, CA, United States of America; Kaohsiung Medical University, TAIWAN

## Abstract

**Introduction:**

Guidelines advocate the treatment of HCV in all HIV/HCV co-infected individuals. The aim of this randomized, open-label study (ClinicalTrials.gov identifier: NCT02707601; https://clinicaltrials.gov/ct2/show/NCT02707601) was to evaluate the safety/efficacy of ledipasvir/sofosbuvir (LDV/SOF) co-administered with elvitegravir/cobicistat/emtricitabine/tenofovir alafenamide (E/C/F/TAF) or rilpivirine/F/TAF (R/F/TAF) in HIV-1/HCV co-infected participants.

**Methods:**

Participants with HIV-1 RNA <50 copies/mL and chronic HCV-genotype (GT) 1 (HCV treatment-naïve ± compensated cirrhosis or HCV treatment-experienced non-cirrhotic) were randomized 1:1 to switch to E/C/F/TAF or R/F/TAF. If HIV suppression was maintained at Week 8, participants received 12 weeks of LDV/SOF. The primary endpoint was sustained HCV virologic response 12 weeks after LDV/SOF completion (SVR12).

**Results:**

Of 150 participants, 148 received ≥1 dose of HIV study drug and 144 received LDV/SOF (72 in each F/TAF group; 83% GT1a, 94% HCV treatment-naïve, 12% cirrhotic). Overall, SVR12 was 97% (95% confidence interval: 93–99%). Black race did not affect SVR12. Of four participants not achieving SVR12, one had HCV relapse, one had HCV virologic non-response due to non-adherence, and two missed the post-HCV Week 12 visit. Of 148 participants, 96% receiving E/C/F/TAF and 95% receiving R/F/TAF maintained HIV suppression at Week 24; no HIV resistance was detected. No participant discontinued LDV/SOF or E/C/F/TAF due to adverse events; one participant discontinued R/F/TAF due to worsening of pre-existing hypercholesterolemia. Renal toxicity was not observed in either F/TAF regimen during LDV/SOF co-administration. In conclusion, high rates of HCV SVR12 and maintenance of HIV suppression were achieved with LDV/SOF and F/TAF-based regimens.

**Conclusion:**

This study supports LDV/SOF co-administered with an F/TAF-based regimen in HIV-1/HCV-GT1 co-infected patients.

## Introduction

Current HIV and HCV guidelines advocate the treatment of HCV in all HIV/HCV co-infected individuals [1–3]. However, recent studies suggest that <50% of HIV/HCV-infected patients have been successfully treated for HCV [4, 5]. This is despite the introduction of direct-acting antiviral agents (DAAs), which have increased sustained virologic response (SVR) rates and shortened therapy durations [6]. The single-tablet regimen (STR) of ledipasvir/sofosbuvir (LDV/SOF) combines two DAAs active against HCV NS5A and NS5B, respectively. Phase 3 clinical trials have shown high rates (94–99%) of SVR at 12 weeks post-treatment (SVR12) in individuals mono-infected with HCV-genotype (GT) 1 [7–9]. Furthermore, LDV/SOF has been associated with high SVR12 rates in individuals co-infected with HIV/HCV-GT1 or HIV/HCV-GT4. The phase 3 ION-4 study evaluated 12 weeks of LDV/SOF treatment in 335 individuals co-infected with HIV and HCV (98% GT1, 2% GT4). Overall, 96% of individuals achieved SVR12 [10], with significant improvement in health-related quality of life [11]. Combined analysis of the ION-1-3 (HCV mono-infection) and ION-4 (HIV/HCV co-infection) studies in 865 participants found that LDV/SOF efficacy was not affected by the presence of HIV infection [12]. Clinical cohort studies have demonstrated high SVR12 rates for LDV/SOF treatment in HIV/HCV co-infected individuals, consistent with clinical trial findings [13–17].

The potential for undesirable drug–drug interactions (DDIs) remains a major consideration when treating HCV in HIV co-infected individuals. Some HIV/HCV regimen combinations are contraindicated [18]. Plasma tenofovir (TFV) concentrations are elevated when tenofovir disoproxil fumarate (TDF) is given as part of regimens pharmacokinetically boosted with ritonavir or cobicistat; addition of LDV/SOF can further elevate plasma TFV levels [2, 19]. Hence treatment guidelines recommend changing the antiretroviral (ARV) regimen from a boosted TDF-containing regimen when LDV/SOF is used to treat HCV [2]. Since tenofovir alafenamide (TAF) has 80–91% lower plasma TFV concentrations than TDF, the renal safety of concomitant treatment with LDV/SOF may be improved with TAF compared to TDF-based regimens [20]. Two phase 1 studies in healthy participants evaluated DDIs between LDV/SOF and two F/TAF-based antiretroviral therapy (ART) regimens: rilpivirine/emtricitabine/TAF (R/F/TAF) or elvitegravir/cobicistat/emtricitabine/TAF (E/C/F/TAF) [21, 22]. LDV/SOF co-administered with R/F/TAF modestly increased plasma TFV exposure, however, TFV area under the curve (AUC) levels remained substantially lower than TFV exposures from TDF-containing regimens (362 vs 2000–5000 ng.h/mL; R/F/TAF + LDV/SOF vs R/F/TDF). Co-administering LDV/SOF with E/C/F/TAF yielded modestly increased cobicistat AUC levels (17,000 vs 11,400 ng.h/mL for E/C/F/TAF alone), but did not alter TFV AUC levels. R/F/TAF did not affect LDV, SOF, and GS-331007 (SOF metabolite) levels, while E/C/F/TAF modestly increased levels of LDV, SOF, and GS-331007 (within the exposure-safety window levels defined by clinical data). Thus, the data in healthy volunteers do not suggest any safety or efficacy concerns with LDV/SOF treatment in HIV-infected patients receiving F/TAF-based regimens.

No study to date has evaluated the safety or efficacy of LDV/SOF when co-administered with either E/C/F/TAF or R/F/TAF for HIV/HCV co-infected patients. Therefore, Co-STARs (Co-infection treatment with Single-TAblet RegimenS) was conducted to evaluate the efficacy, safety, and tolerability of switching from a stable three-drug regimen to E/C/F/TAF or R/F/TAF followed by treatment with LDV/SOF in HIV/HCV-GT1 participants.

## Methods

### Study design

This phase 3b randomized, open-label study (NCT02707601) was conducted across 44 centers in the US between April 2016 and September 2017 in two parts. In Part 1, participants on stable ART were stratified by race (black or non-black) and then randomized 1:1 in parallel to receive 8 weeks of one of two F/TAF-based STRs, both administered orally, once daily with food. Participants received either elvitegravir 150 mg, cobicistat 150 mg, emtricitabine 200 mg, and TAF 10 mg (E/C/F/TAF; Genvoya^®^, Gilead Sciences, Inc.) or emtricitabine 200 mg, rilpivirine 25 mg, and TAF 25 mg (R/F/TAF; Odefsey^®^, Gilead Sciences, Inc.). Randomization was conducted prior to or during the day 1 visit by the investigator or designee using an Interactive Web Response System (utilizing unique subject numbers assigned to each participant following screening).

In Part 2 at Week (W) 8, all participants with HIV viral suppression (<50 copies/mL) received 12 weeks of LDV 90 mg and SOF 400 mg as an STR (Harvoni^®^, Gilead Sciences, Inc.) administered orally, once daily with or without food, and remained on their assigned HIV treatment. Participants who had HIV RNA ≥50 copies/mL or who did not tolerate F/TAF-based therapy during Part 1 did not continue to the 12-week HCV treatment phase. At W20, after completion of HCV treatment, ART was maintained. Participants continued in the study for another 12 (post-HCV) weeks to determine SVR4 and SVR12.

The study was approved by the institutional review board or independent ethics committee at each participating site (full list provided in the S1 Table of the Supplementary Material). The study conformed to Good Clinical Practice guidelines and Declaration of Helsinki Principles and all participants provided written informed consent.

### Study population

Participants were HIV-1/HCV-GT1 co-infected adults (aged ≥18 years). Key inclusion criteria were use of a stable ART (two nucleoside/nucleotide reverse transcriptase inhibitors plus either a protease inhibitor, integrase inhibitor, or non-nucleoside reverse transcriptase inhibitor); maintenance of HIV RNA <50 copies/mL for at least 6 months prior to screening; and no history of HIV virologic failure. Participants had chronic HCV-GT1 infection and were HCV treatment-naïve (with or without cirrhosis) or interferon-based treatment-experienced (without cirrhosis). Key exclusion criteria included prior HCV treatment with HCV DAAs (except boceprevir, telaprevir, and simeprevir in combination with interferon ± ribavirin), chronic liver disease of a non-HCV etiology, pregnancy, malignancy including hepatocellular carcinoma, decompensated liver disease, or evidence of hepatitis B virus infection. Full inclusion and exclusion criteria are provided in the S1 Text and S2 Text of the Supplementary Material.

### Endpoints and assessments

The primary endpoint was SVR12 (plasma HCV RNA below lower limit of quantification [LLOQ; 15 IU/mL] 12 weeks post-LDV/SOF treatment). Secondary endpoints were SVR4 (HCV RNA below LLOQ 4 weeks post-LDV/SOF treatment), HIV-1 RNA ≥50 copies/mL (HIV virologic failure) 24 weeks after switching to E/C/F/TAF or R/F/TAF based on FDA-defined snapshot algorithm, and Grade 1–4 AEs throughout the study and during co-administration with LDV/SOF.

Full details of assessments performed at each study visit are provided in the S3 Text of the Supplementary Material; laboratories (done centrally) included HIV RNA, HCV RNA, chemistry, hematology, and fasting metabolic parameters.

Historical HIV GT reports or sequencing of HIV baseline samples (proviral DNA) (Monogram Biosciences, Inc., South San Francisco, CA, USA) were required for study entry to screen for any pre-existing resistance-associated mutations related to study drugs. During the study, genotypic and phenotypic resistance testing of reverse transcriptase, protease, and integrase (Monogram Biosciences, Inc.) were performed on any participant who had a confirmed HIV-1 RNA ≥50 copies/mL (within 2–4 weeks of the first sample) and the confirmation HIV-1 RNA ≥400 copies/mL, or at the last visit on study drug. For participants with HCV virologic failure, HCV NS5A and NS5B coding regions were amplified by reverse transcriptase polymerase chain reaction and deep sequenced using the Illumina MiSeq deep sequencing platform (DDL Diagnostic Laboratory, Rijswijk, the Netherlands).

### Statistical analysis

In the primary efficacy analysis, the SVR12 rate was compared to a performance goal of 88% using a two-sided exact one-sample binomial test at the 0.05 significance level, with associated two-sided exact 95% confidence interval (CI) based on Clopper–Pearson method. This performance goal was based on overall high rates of SVR12 in DAA therapy with HIV/HCV co-infected participants. To assess the relationship between SVR4/SVR12 and baseline demographic and disease characteristics, 95% CIs were generated using the same method as the primary efficacy analysis for subgroup analyses. HCV analyses included participants receiving ≥1 dose of LDV/SOF; participants who discontinued during Part 1 (prior to W8) were not included in HCV analyses (pre-determined analytical plan).

The secondary HIV efficacy endpoint (HIV-1 RNA ≥50 copies/mL 24 weeks after switch) was assessed using the FDA-defined snapshot algorithm for participants receiving ≥1 dose of HIV study drug and hence included participants who discontinued study during Part 1.

In order to assess laboratory abnormalities and AEs due to co-administration of LDV/SOF with F/TAF regimens, the abnormalities/events were evaluated in three time periods: i) from Day 1 up to W8 (during change to F/TAF-based regimen), ii) during the co-administration period (W8–20, events prior to W8 were not included), and iii) for the entire study duration. Safety data were summarized according to the randomized HIV treatment group and overall.

SAS software V9.4 (SAS Institute Inc., Cary, NC, USA) was used for statistical analyses.

A sample size of 240 participants was originally planned for the study, but was reduced with a protocol amendment (to approximately 120 participants) due to slow accrual and consensus that the study objectives could be achieved with fewer participants. A sample size of 120 provides ≥85% power to detect an improvement of ≥8% points in SVR12 rate from the performance goal of 88%.

## Results

### Participants

**Fig 1:**
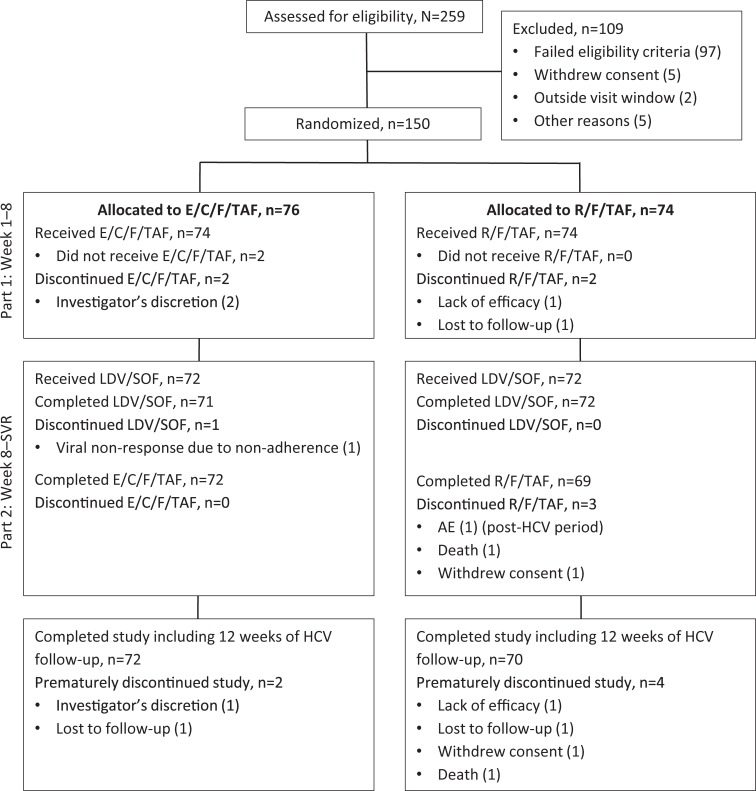
Disposition of participants. AE, adverse event; E/C/F/TAF, elvitegravir/cobicistat/emtricitabine/tenofovir alafenamide; LDV/SOF, ledipasvir/sofosbuvir; R/F/TAF, rilpivirine/ emtricitabine/tenofovir alafenamide; SVR, sustained virologic response.

Participants (n = 150) were randomized 1:1 to switch their ART regimen to either E/C/F/TAF or R/F/TAF in Part 1 of the study (Fig 1). Overall, 74 participants switched to each regimen. Four participants discontinued the study in Part 1 (Fig 1, two participants never received the F/TAF regimen). In total, 144 participants entered Part 2 of the study, the LDV/SOF co-administration period, and continued HIV study drug treatment. One participant in the E/C/F/TAF treatment arm discontinued LDV/SOF treatment due to HCV virologic non-response associated with non-adherence. In the Post-HCV period, three participants discontinued R/F/TAF (Fig 1).

Baseline demographics were similar between the participant groups randomized to E/C/F/TAF or R/F/TAF (Table 1). Overall, the median age of participants was 53 years (range 25–70); most were male (74%) and 41% were black. Baseline HIV and HCV disease characteristics were generally similar between randomized treatment groups (Table 1). Overall, baseline median CD4 cell count was 651 cells/μL, and 74% of participants had a CD4 cell count ≥500 cells/μL. The median duration of prior ART was 13 years. Participants starting LDV/SOF (Part 2 of the study) had a median HCV RNA of 6.4 log_10_ IU/mL, and most (72%) had HCV RNA ≥800,000 IU/mL. Overall, 94% of participants were HCV treatment-naïve, and 12% had cirrhosis.

**Table 1:** Demographics and HIV and HCV disease characteristics of study participants per randomized HIV treatment group.

	E/C/F/TAF (n = 74)	R/F/TAF (n = 74)	Total (N = 148)
**Demographics**^a^			
Median age, years (range)	52 (26–70)	55 (25–69)	53 (25–70)
Male, n (%)	58 (78)	52 (70)	110 (74)
Race, n (%)			
White	41 (55)	37 (50)	78 (53)
Black	30 (41)	31 (42)	61 (41)
Other	3 (4)	6 (8)	9 (6)
**HIV disease characteristics**^a^			
CD4 count, cells/μL; median (1^st^–3^rd^ quartile)	671 (450–830)	640 (507–795)	651 (484–806)
eGFR_CG_, mL/min; median (1^st^–3^rd^ quartile)	99 (79–115)	100 (75–118)	100 (77–117)
Duration of prior ART, years median (range)	12 (1–31)	16 (1–32)	13 (1–32)
ART type received immediately prior to first dose of study drug, n (%)^b^			
INSTI	41 (55)	32 (43)	73 (49)
NNRTI	28 (38)	23 (31)	51 (34)
PI ± PK boost	5 (7)	16 (22)	21 (14)
NRTI	72 (97)	73 (99)	145 (98)
TAF or TDF	58 (78)	56 (76)	114 (77)
ABC	14 (19)	15 (20)	29 (20)
Other	0	2 (3)	2 (1)
**HCV disease characteristics (at Part 2 baseline)**^c^	**n = 72**	**n = 72**	**N = 144**
HCV genotype, n (%)			
1a	62 (86)	58 (81)	120 (83)
1b	10 (14)	13 (18)	23 (16)
1, unknown subtype^d^	0	1 (1)	1 (1)
HCV RNA, log_10_ IU/mL, median (range)	6.4 (1.1–7.3)	6.5 (4.3–7.5)	6.4 (1.1–7.5)
HCV RNA category, ≥800,000 IU/mL, n (%)	53 (74)	51 (71)	104 (72)
HCV treatment-experienced, n (%)^e^	6 (8)	3 (4)	9 (6)
ALT >1.5 x ULN, n (%)	24 (33)	23 (32)	47 (33)
Cirrhosis, n (%)^f^	8 (11)	9 (13)	17 (12)
IL28B CC genotype, n (%)	21 (29)	16 (22)	37 (26)

^a^Baseline values were determined at the Day 1 study visit.

^b^One participant in the E/C/F/TAF group and two in the R/F/TAF group were excluded due to missing data.

^c^Baseline HCV disease characteristics were determined at the Week 8 study visit.

^d^One participant in the R/F/TAF group had HCV genotype 1 of unknown subtype (neither 1a or 1b).

^e^Prior HCV treatment constituted interferon + ribavirin ± HCV PI (boceprevir, telaprevir, or simeprevir only).

^f^Definition of cirrhosis provided in the S1 Text (inclusion criteria number 11) of the Supplementary Material.

ABC, abacavir; ALT, alanine aminotransferase; ART, antiretroviral therapy; E/C/F/TAF, elvitegravir/cobicistat/emtricitabine/tenofovir alafenamide; eGFR_CG_, estimated glomerular filtration rate calculated using the Cockcroft–Gault equation; INSTI, integrase strand transfer inhibitor; NNRTI, non-nucleoside reverse transcriptase inhibitor; NRTI, nucleoside reverse transcriptase inhibitor; PI, protease inhibitor; PK, pharmacokinetic; R/F/TAF, rilpivirine/emtricitabine/tenofovir alafenamide; TAF, tenofovir alafenamide; TDF, tenofovir disoproxil fumarate; ULN, upper limit of normal.

### Efficacy

#### HCV-related efficacy

**Fig 2:**
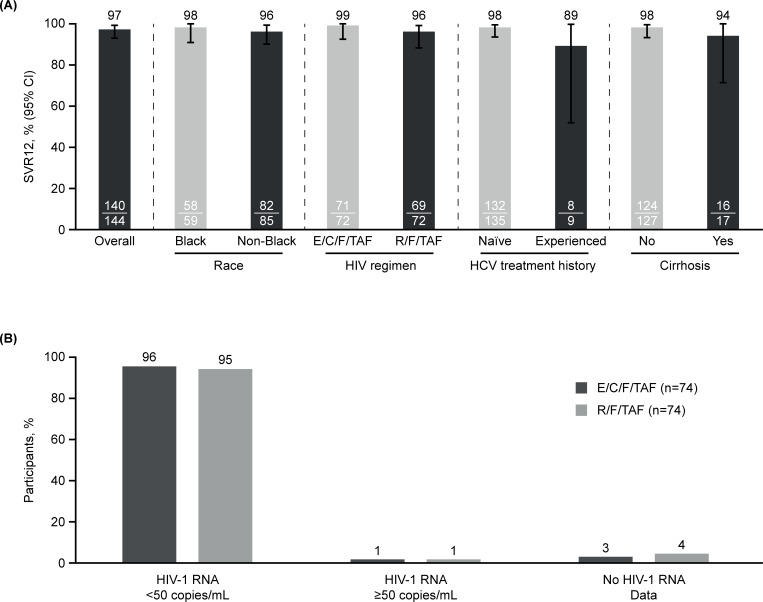
Main HCV and HIV-related efficacy outcomes. (A) SVR12 overall (primary endpoint) and according to race, HIV TAF regimen, HCV treatment history, and cirrhosis status. (B) HIV virologic outcome (HIV RNA ≤50 copies/mL) at Week 24 by FDA-defined snapshot algorithm. CI, confidence interval; E/C/F/TAF, elvitegravir/cobicistat/emtricitabine/tenofovir alafenamide; R/F/TAF, rilpivirine/ emtricitabine/tenofovir alafenamide; SVR12, sustained virologic response 12 weeks post-HCV treatment; TAF, tenofovir alafenamide.

The overall proportion of participants achieving SVR12 (primary endpoint) was 97% (95% CI: 93–99%; Fig 2A), which demonstrated superiority over the performance goal of 88% (p<0.001). SVR12 was observed to be similar across all subgroups of baseline demographic and disease characteristics including race, HCV treatment history, and cirrhosis status (Fig 2A, Table 2). SVR12 rates were comparable between E/C/F/TAF and R/F/TAF treatment groups, 99% and 96%, respectively (Fig 2A). The overall SVR4 rate was 99% (95% CI: 95–100%), which exceeded the 88% performance goal (p<0.001).

**Table 2:** HCV virologic response (SVR12) following 12 weeks of ledipasvir/sofosbuvir treatment overall and by subgroup.

Participants, n (%)	E/C/F/TAF (n = 72)	R/F/TAF (n = 72)	Total (N = 144)
All participants	71/72 (99)	69/72 (96)	140/144 (97)
HCV genotype			
1a	61/62 (98)	55/58 (95)	116/120 (97)
1b	10/10 (100)	13/13 (100)	23/23 (100)
1, sub-type unknown	0	1/1 (100)	1/1 (100)
Age at baseline, years			
<65	64/65 (98)	66/69 (96)	130/134 (97)
≥65	7/7 (100)	3/3 (100)	10/10 (100)
Race			
Black	29/30 (97)	29/29 (100)	58/59 (98)
Non-black	42/42 (100)	40/43 (93)	82/85 (96)
Baseline HCV RNA (IU/mL)^a^			
<800,000	19/19 (100)	21/21 (100)	40/40 (100)
≥800,000	52/53 (98)	48/51 (94)	100/104 (96)
Cirrhosis			
Yes	8/8 (100)	8/9 (89)	16/17 (94)
No	63/64 (98)	61/63 (97)	124/127 (98)
Prior HCV treatment experience^b^			
Treatment- naïve	65/66 (98)	67/69 (97)	132/135 (98)
Treatment- experienced	6/6 (100)	2/3 (67)	8/9 (89)

^a^Baseline refers to Part 2 baseline, determined at the Week 8 study visit.

^b^Prior HCV treatment constituted interferon + ribavirin ± HCV protease inhibitor (boceprevir, telaprevir, or simeprevir only).

E/C/F/TAF, elvitegravir/cobicistat/emtricitabine/tenofovir alafenamide; R/F/TAF, rilpivirine/ emtricitabine/tenofovir alafenamide; SVR12, sustained virologic response 12 weeks post-HCV treatment.

Four participants did not achieve SVR12, one receiving E/C/F/TAF and three receiving R/F/TAF. The participant receiving E/C/F/TAF was a 52-year-old black female, who experienced HCV virologic non-response (HCV RNA 82 IU/mL and 1.6 million IU/mL after 4 and 8 weeks of LDV/SOF treatment, respectively). Virologic failure was determined at W8 of LDV/SOF treatment, and the resistance-associated substitutions Q30R and H58D in NS5A were identified, which were not present at baseline. Although the participant had 93% LDV/SOF adherence by pill count, the participant had undetectable levels of LDV at W8 (and level below the 95% percentile of population pharmacokinetic values at W4) and undetectable GS-331007 at W4 and W8 of LDV/SOF therapy, suggesting suboptimal adherence. Of three R/F/TAF participants without SVR12, one participant had HCV relapse (achieved SVR4, but had HCV RNA 4.4 million IU/mL at Post-HCV W12). The relapsing participant was cirrhotic and had no evidence of resistance-associated substitutions, either at baseline or at relapse. Deep sequence analysis of baseline and relapse isolates did not suggest HCV re-infection; both NS5A and NS5B genes have 98.7% homology at the nucleotide level between baseline and relapse time periods. Two participants missed the Post-HCV W12 visit and did not attain SVR12; one due to death from metastatic carcinoma of unknown primary site between treatment completion and the Post-HCV W4 visit (the participant had HCV RNA below LLOQ at completion of LDV/SOF treatment), and the other due to withdrawal of consent after achieving SVR4.

#### HIV-related efficacy

Overall, 96% (71/74) and 95% (70/74) of participants randomized to E/C/F/TAF and R/F/TAF, respectively, maintained HIV-1 RNA <50 copies/mL at W24 as determined by the FDA-defined snapshot algorithm (Fig 2B). A total of seven participants were considered failures by the algorithm. Two participants (one [1%] in each group) had HIV-1 RNA ≥50 copies/mL at W24. One R/F/TAF participant discontinued study medication due to an AE (worsening of pre-existing hypercholesterolemia; HIV RNA <50 copies/mL) and was counted as a snapshot failure. This participant subsequently received F/TAF plus dolutegravir. Four participants discontinued HIV study drug prior to W8 (Fig 1: lack of efficacy [R/F/TAF n = 1], investigator’s discretion [E/C/F/TAF n = 2], lost to follow-up [R/F/TAF n = 1]). No participant developed HIV drug resistance mutations after switching to either F/TAF-based regimen. Mean change (standard deviation) from baseline in CD4 cell count at W24 was +25 (149.7) cells/μL for E/C/F/TAF and +59 (232) cells/μL for R/F/TAF (p = 0.30).

### Safety

For the entire study duration, AEs of any grade, Grade 3 or 4, and serious AEs were reported in 82%, 12%, and 13% of participants, respectively (Table 3). The most common AEs observed throughout the study were cough (11%), upper respiratory tract infection (9%), and headache, arthralgia, and urinary tract infection (all 8%). AEs ≥Grade 3 observed in more than one participant were increased alanine aminotransferase (n = 2) and increased aspartate aminotransferase (n = 2); all received E/C/F/TAF. Three serious AEs occurred during Part 1 and 12 during Part 2, but none was considered study drug-related. Overall, the majority of laboratory abnormalities were Grade 1 (n = 49; 33.3%) or Grade 2 (n = 56; 38.1%) in severity. Generally, the incidence of Grade 3 or 4 laboratory abnormalities by analyte was comparable between Part 1 and the co-administration period. Low-density lipoprotein elevation was the most common Grade 3 laboratory abnormality (no Grade 4) and occurred during the co-administration period, but since low-density lipoprotein was not measured until W8, the effect of HIV or HCV regimen could not be assessed (Table 3). The incidence of AEs in either part of the study or during the whole study was similar between randomized HIV treatment groups. Overall 63% and 69% of participants receiving E/C/F/TAF and R/F/TAF, respectively, experienced AEs during the co-administration period and there were no treatment discontinuations due to clinical AEs during co-administration. One participant discontinued R/F/TAF during the Post-HCV period due to worsening of pre-existing hypercholesterolemia. One participant in the R/F/TAF group died due to metastatic carcinoma (unknown primary site) after completion of LDV/SOF but prior to the Post-HCV W4 visit.

**Table 3:** Most common AEs (≥5%) and Grade 3–4 laboratory abnormalities (≥3%) by study period and antiretroviral regimen.

Participants, n (%)	Part 1; Day 1–W8 ARVs only			Part 2; W8–W20 ARVs + ledipasvir/sofosbuvir			Whole study; Day 1–end		
	E/C/F/ TAF (n = 74)	R/F/ TAF (n = 74)	Total (N = 148)	E/C/F/ TAF (n = 72)	R/F/ TAF (n = 72)	Total (N = 144)	E/C/F/ TAF (n = 74)	R/F/ TAF (n = 74)	Total, (N = 148)
**AEs**									
Any AE	38 (51)	39 (53)	77 (52)	45 (63)	50 (69)	95 (66)	62 (84)	59 (80)	121 (82)
Grade 2, 3, or 4 AE	11 (15)	13 (18)	24 (16)	20 (28)	22 (31)	42 (29)	33 (45)	31 (42)	64 (43)
Grade 3 or 4 AE	3 (4)	2 (3)	5 (3)	5 (7)	5 (7)	10 (7)	9 (12)	8 (11)	17 (11)
Study drug-related AE	5 (7)	5 (7)	10 (7)	7 (10)	7 (10)	14 (10)	13 (18)	9 (12)	22 (15)
Any SAE	1 (1)	2 (3)	3 (2)	4 (6)	8 (11)	12 (8)	8 (10)	12 (16)	19 (13)
Study drug-related SAE	0	0	0	0	0	0	0	0	0
AE leading to DC of HIV study drug	0	1 (1)^a^	1 (1)^a^	0	1 (1)^a^	1 (1)^a^	0	1 (1)^a^	1 (1)^a^
AE leading to DC of HCV study drug	0	0	0	0	0	0	0	0	0
Death	0	0	0	0	0	0	0	1 (1)^b^	1 (1)^b^
**Common AEs (≥5%)**									
Cough	2 (3)	5 (7)	7 (5)	3 (4)	4 (6)	7 (5)	6 (8)	10 (14)	16 (11)
Upper respiratory tract infection	5 (7)	0	5 (3)	4 (6)	4 (6)	8 (6)	9 (12)	5 (7)	14 (9)
Headache	1 (1)	2 (3)	3 (2)	3 (4)	8 (11)	11 (8)	4 (5)	8 (11)	12 (8)
Arthralgia	1 (1)	3 (4)	4 (3)	5 (7)	3 (4)	8 (6)	7 (9)	5 (7)	12 (8)
Urinary tract infection	3 (4)	1 (1)	4 (3)	3 (4)	1 (1)	4 (3)	10 (14)	2 (3)	12 (8)
Nausea	2 (3)	3 (4)	5 (3)	1 (1)	4 (6)	5 (3)	4 (5)	6 (8)	10 (7)
Fatigue	0	2 (3)	2 (1)	4 (6)	3 (4)	7 (5)	4 (5)	5 (7)	9 (6)
Diarrhea	1 (1)	2 (3)	3 (2)	4 (6)	1 (1)	5 (3)	6 (8)	3 (4)	9 (6)
Abdominal pain	1 (1)	0	1 (1)	1 (1)	4 (6)	5 (3)	4 (5)	4 (5)	8 (5)
**Grade 3–4 laboratory abnormalities**									
Any Grade 3 or 4 laboratory abnormality	8/74 (11)	10/73 (14)	18/147 (12)	10/72 (14)	5/72 (7)	15/144 (10)	18/74 (24)	16/73 (22)	34/147 (23)
Serum glucose elevation (fasting)	2/74 (3)	2/73 (3)	4/147 (3)	0/72	2/72 (3)	2/144 (1)	2/74 (3)	4/73 (5)	6/147 (4)
LDL elevation^c^ (evaluated Day 1, W8, W20, and SVR12 visits)	0/72	0/71	0/143	6/72 (8)	1/72 (1)	7/144 (5)	7/72 (10)	3/72 (4)	10/144 (7)
Hematuria (quantitative)	1/26 (4)	2/35 (6)	3/61 (5)	2/44 (5)	1/56 (2)	3/100 (3)	3/57 (5)	3/65 (5)	6/122 (5)
Glycosuria (urine dipstick)	1/74 (1)	2/73 (3)	3/147 (2)	0/72	2/72 (3)	2/144 (1)	1/74 (1)	4/73 (5)	5/147 (3)
Elevated prothrombin time	1/72 (1)	1/73 (1)	2/145 (1)	1/72 (1)	1/72 (1)	2/144 (1)	3/73 (4)	2/73 (3)	5/146 (3)

AEs are displayed by study period to evaluate the impact of co-administration on tolerability.

^a^One participant had an AE leading to discontinuation of HIV study drug, worsening of hypercholesterolemia, which began in Part 1, continued in Part 2, and resulted in discontinuation of HIV study drug at Post-HCV W4.

^b^Death from metastatic carcinoma of unknown primary site between treatment completion and the Post-HCV W4 visit.

^c^LDL was not evaluated in Part 1 (Day 1–W8), so no events could be found in this period.

AE, adverse event; ARV, antiretroviral; DC, discontinuation; E/C/F/TAF, elvitegravir/cobicistat/emtricitabine/tenofovir alafenamide; LDL, low-density lipoprotein; R/F/TAF, rilpivirine/emtricitabine/tenofovir alafenamide; SAE, serious adverse event; SVR12, sustained virologic response 12 weeks post-HCV treatment; W, week.

**Fig 3:**
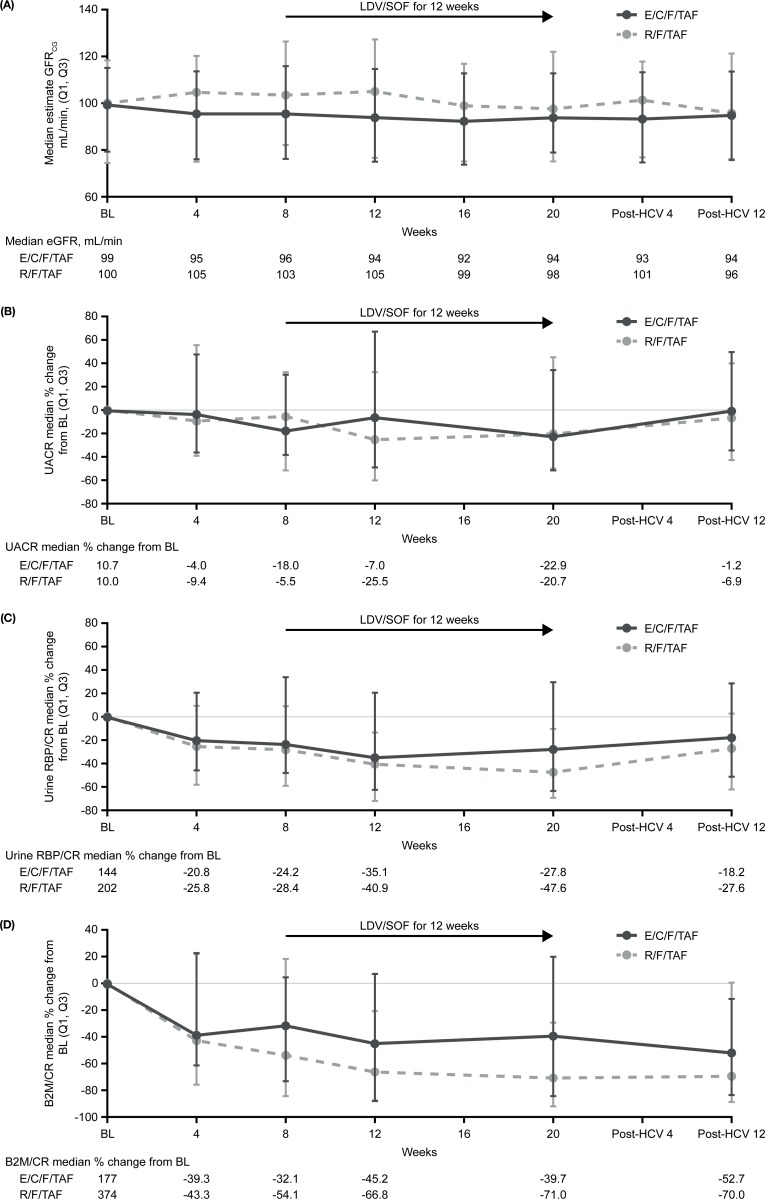
**Renal safety as measured by eGFR**_**CG**_^**a**^ **(A), urine albumin to creatinine ratio**^**b**^ **(B), urine RBP to creatinine ratio**^**b**^ **(C), and urine beta-2-microglobulin to creatinine ratio**^**c**^ **(D) plotted as % change from baseline over time by TAF regimen.** ^a^For eGFR_CG_, measurements were taken from 74 versus 74 patients at baseline and 72 versus 69 patients at the Post-HCV W12 visit in the E/C/F/TAF group versus R/F/TAF groups, respectively. ^b^For UACR and for urine RBP to creatinine ratio, measurements were taken from 73 versus 74 patients at baseline and 71 versus 69 patients at the Post-HCV W12 visit in the E/C/F/TAF group versus R/F/TAF groups, respectively. ^c^For urine B2M to creatinine ratio, measurements were taken from 73 versus 72 patients at baseline and 72 versus 68 patients at the Post-HCV W12 visit in the E/C/F/TAF group versus R/F/TAF groups, respectively. BL, baseline; B2M, beta-2-microglobulin; CR, creatinine ratio; E/C/F/TAF, elvitegravir/cobicistat/emtricitabine/tenofovir alafenamide; eGFR, estimated glomerular filtration rate; eGFR_CG_, estimated glomerular filtration rate calculated using the Cockcroft–Gault equation; LDV/SOF, ledipasvir/sofosbuvir; Q, quartile; RBP, retinol binding protein; R/F/TAF, rilpivirine/emtricitabine/tenofovir alafenamide; TAF, tenofovir alafenamide; UACR, urine albumin to creatinine ratio; W, week.

Minimal changes were seen in median estimated glomerular filtration calculated using the Cockcroft–Gault equation (eGFR_CG_) for both F/TAF-based regimens throughout the study (Fig 3A). Overall, median baseline eGFR_CG_ was 99.8 mL/min, with the median change 2.2 mL/min at W8, ^™^0.9 mL/min at Post-HCV W4, and ^™^0.1 mL/min at Post-HCV W12. Two participants experienced a serum creatinine (SCr) rise of >0.4 mg/dL. One 54-year-old white male with cirrhosis, who switched from efavirenz/abacavir/lamivudine to R/F/TAF, experienced a 0.5 mg/dL increase in SCr from baseline (1.28 mg/dL) to 1.79 mg/dL at W14, which remained elevated throughout study follow-up. There was no increase in his pre-existing trace proteinuria and no glycosuria. Despite SCr increase, there were marked reductions from baseline in urine ratios of albumin, retinol binding protein, and beta-2-microglobulin to creatinine compared with baseline (findings not consistent with TFV-related tubulopathy). HIV and HCV drug regimens were unchanged. A 50-year-old black male with hypertension (on four antihypertensive medications) and cirrhosis randomized to R/F/TAF had elevated SCr at screening of 2.4 mg/dL; baseline SCr was 1.25 mg/dL. The participant developed elevated SCr of 3.25 mg/dL at W8 (just prior to HCV treatment). No action was taken with R/F/TAF, but LDV/SOF doses were interrupted for 1 week. After intravenous saline, SCr returned to baseline levels. Both participants with elevated SCr had undetectable HIV RNA and achieved HCV SVR12.

Quantitative measures of urine protein (urine ratios of albumin, retinol binding protein, and beta-2-microglobulin to creatinine) were reduced after switch to an F/TAF-based regimen; these reductions were maintained after the addition of LDV/SOF and for the duration of the study (Fig 3B–3D). For example, the overall median urine albumin to creatinine ratio for both regimens was 10 mg/g at Day1, with a percent change of –11% at W8, –23% at W20 (end of LDV/SOF), and –5% at the SVR12 visit. Both F/TAF-based regimens had similar changes in renal protein markers.

## Discussion

This study is the first to evaluate LDV/SOF co-administration with both boosted and unboosted TAF-based regimens in HIV/HCV co-infected patients. The findings demonstrate that a once-daily STR of LDV/SOF is highly effective for the treatment of HCV-GT1 in HIV/HCV co-infected individuals receiving E/C/F/TAF or R/F/TAF, with an SVR12 rate of 97%. Race, HIV regimen, HCV treatment history, and cirrhosis status had no impact on HCV treatment outcome. HCV virologic failure rates were low; only two participants (1%) had true virologic failure (one HCV relapse, one HCV virologic non-response). The participant with HCV virologic non-response was non-adherent to LDV/SOF, based on pill counts and low or undetectable LDV and SOF metabolite levels, and subsequently developed NS5A resistance mutations at positions Q30R and H58D (mutations previously associated with LDV resistance) [23]. The two other participants who did not attain SVR12 were imputed as failure, as they did not attend the Post-HCV W12 visit, although they had HCV RNA below LLOQ at the end of treatment and the SVR4 visit, respectively.

The overall SVR12 rate is in line with other evaluations of LDV/SOF efficacy in HIV/HCV co-infection, both in the ION-4 clinical trial [10] and in clinical cohort studies [13–17]. However, ION-4 reported a significantly lower SVR12 for black compared with non-black participants (90% and 99%, respectively). Racial differences were not observed in this study; SVR12 rates were comparable between black and non-black participants (98% and 96%, respectively). Clinical cohort data from HIV/HCV co-infected cohorts (mostly GT1) and predominantly treated with LDV/SOF also demonstrate high SVR12 rates in black individuals [14], indicating that race may not be a barrier to successful HCV treatment with LDV/SOF in HCV, particularly in GT1 infection.

Current guidelines indicate that HCV-GT1 infection should be treated the same in HIV/HCV co-infected individuals as in mono-infected individuals: 12 weeks of LDV/SOF is recommended [1–3], with the caveat that the potential for DDIs with ARVs should be considered to avoid AEs [2, 3, 19, 24]. In the current study, most clinical AEs were mild in severity, and there were no HIV or HCV treatment discontinuations due to clinical AEs. The frequencies of AEs were generally comparable (<5% difference) during the initial 8 weeks of study (ARV alone) and during the co-administration period, indicating that the introduction of LDV/SOF to an F/TAF-based regimen did not reduce tolerability. Minimal differences in clinical AEs and laboratory abnormalities observed during LDV/SOF co-administration suggest that any minor differences in HIV and HCV drug exposures are not clinically meaningful when LDV/SOF is combined with either E/C/F/TAF or R/F/TAF regimens. Furthermore, this study confirmed pharmacokinetic data in healthy volunteers showing no clinical risk of renal toxicity when either boosted (E/C/F/TAF) or unboosted (R/F/TAF) TAF-based regimens were co-administered with 12 weeks of LDV/SOF [21]; there were minimal changes seen in eGFR_CG_ and improvements in urinary protein markers for participants receiving both regimens.

HIV treatment guidelines state that ART regimens may need to be switched or modified prior to initiation of HCV treatment to reduce the potential for DDIs, but HIV suppression must be maintained [2, 25]. In this study, maintenance of HIV suppression was achieved in 95% of participants following a switch of ART to E/C/F/TAF or R/F/TAF. HIV virologic failure was rare (1% in each arm) and no participant developed HIV drug resistance. This high rate of maintained viral suppression is consistent with results from other switch studies [26–31]. Importantly, co-administration of LDV/SOF had no effect on ART efficacy, indicating that LDV/SOF can be co-administered with E/C/F/TAF or R/F/TAF to treat HCV without threatening HIV suppression.

Only two of the recommended HCV DAA regimens (LDV/SOF and SOF/velpatasvir) can be used with ‘most’ ARVs according to American Association for the Study of Liver Diseases guidelines [2]. Therefore, using these regimens may facilitate HCV treatment in HIV/HCV co-infected individuals without requiring a switch in ARVs to avoid DDIs. However, convenience and adherence are also important clinical considerations. Our study utilized once-daily STRs for both HIV and HCV treatment, which could be beneficial for adherence and consequent outcomes. The simplicity of the combination of once-daily LDV/SOF STR and an F/TAF-based STR in this study is expected to aid adherence. In support of this, a small observational study has reported comparably high adherence to LDV/SOF between HCV mono-infected and HIV/HCV co-infected participants [32].

Limitations of the current study include lack of blinding, restriction to participants eligible for either of the ART regimens, and restriction to HCV-GT1 (and consequent necessity for HCV genotyping) with no or prior interferon-based regimens ± first-generation HCV protease inhibitors. The number of HCV treatment-experienced (n = 9, 6%) or cirrhotic (n = 11, 12%) participants was small. Finally, LDV/SOF therapy may be shortened to 8 weeks in treatment-naïve individuals without cirrhosis and with baseline HCV RNA <6 million IU/mL according to some international guidelines [1, 3], but not others [2]. This study did not examine an 8-week LDV/SOF regimen.

In conclusion, switching HIV ART regimen to E/C/F/TAF or R/F/TAF followed by treatment of HCV with 12 weeks of LDV/SOF was well tolerated, HIV suppression was maintained, and high rates of SVR12 were achieved in individuals co-infected with HIV/HCV-GT1.

## Supporting information

S1 Table Institutional review board or independent ethics committee at each participating site. (DOCX)

S1 Text Full inclusion criteria. (DOCX)

S2 Text Full exclusion criteria. (DOCX)

S3 Text Assessments performed at each study visit. (DOCX)

S1 File Completed CONSORT checklist. (DOC)

S2 File Original study protocol. (PDF)

S3 File Amended study protocol. (PDF)
