# Supplementary material for: HIV/HCV therapy with ledipasvir/sofosbuvir after randomized switch to emtricitabine-tenofovir alafenamide-based single-tablet regimens
Source: PLoS One. 2020 Jan 29;15(1):e0224875. doi: 10.1371/journal.pone.0224875 (PMC6988963; doi:10.1371/journal.pone.0224875)
Supplement: S1 Table — (DOCX) [file pone.0224875.s001.docx]

**Supplementary Material**

Supplementary Material has been provided by the authors to give readers additional information about their work.

Supplement to: **HIV/HCV therapy with ledipasvir/sofosbuvir after randomized switch to emtricitabine-tenofovir alafenamide-based single-tablet regimens**

Gregory D. Huhn, Moti Ramgopal, Mamta K. Jain, Federico Hinestrosa, David M. Asmuth, Jihad Slim, Deborah Goldstein, Shauna Applin, Julie H. Ryu, Shuping Jiang, Stephanie Cox, Moupali Das, Thai Nguyen-Cleary, David Piontkowsky, Bill Guyer, Lorenzo Rossaro, and Richard H. Haubrich

# S1 Table. Institutional review board or independent ethics committee at each participating site

| **Geographic Region/ Country**  **Principal Investigator (Site Number)** | **IRB or IEC** |
| --- | --- |
| **North America / Puerto Rico**  Morales Ramirez, Javier, MD (0661) | Chesapeake Institutional Review Board  6940 Columbia Gateway Drive  Suite 110  Columbia, Maryland 21046  USA |
|  |  |
| **North America / United States**  Applin, Shauna, MD (12019)  Bartczak, Jennifer, MD (3379)  Benson, Paul, MD (1236)  Brinson, Cynthia, MD (1624)  Coulston, Daniel, MD (0524)  Crofoot, Gordon, MD (2475)  Gathe, Joseph, MK (031)  Goldstein, Deborah, MD (1198)  Hagins, Debbie, MD (4838)  Hassanein, Tarek, MD (530)  Hinestrosa, Frederico, MD (5586)  Kinder, Clifford, MD (2675)  LaMarca, Anthony, MD (0566)  Lucasti, Christopher, MD (0637)  Mayer, Cynthia, MD (2843)  Mills, Anthony, MD (2728)  Osiyemi, Olayemi, MD (2106)  Prelutsky, David, MD (1966)  Prieto, Juan, MD (12942)  Ramgopal, Moti, MD (1950)  Richmond, Gary, MD (1598)  Rodriguez, Benigno , MD (1989)  Ruane, Peter, MD (0407)  Shalit, Peter, MD (0364)  Tebas-Medrano, Pablo, MD (0729)  Thedinger, Blair, MD (12793)  Vanig, Thanes, MD (0754)  Vega, Vilma, MD (898)  Voskuhl, Gene, MD (6046)  Wheeler, David, MD (0550)  Wohl, David, MD (0994)  Wohlfeiler, Michael, MD (2480) | Chesapeake Institutional Review Board  6940 Columbia Gateway Drive  Suite 110  Columbia, Maryland 21046  USA |
| Asmuth, David, MD (843) | UC Davis IRB Administration  2921 Stockton Blvd., CTSC Building, Suite 1400, Room 1429  Sacramento, California 95817  USA |
| Benson, Constance, MD (1601)  Brau, Norbert, MD (5663)  Campo, Rafael, MD (652) | UCSD Human Research Protections Program (HRPP)  Altman Clinical & Translational Institute, Level 2  9452 Medical Center Drive  La Jolla, California 92093  USA  Institutional Review Board – Bronx VA Medical Center Research & Development Program  130 West Kingsbridge Road  1F-01  Bronx, New York 10468  USA  Western Institutional Review Board  1019 39^th^ Avenue SE  Suite 120  Puyallup, Washington 98374-2115  USA |
| Cook, Paul, MD (1808) | University and Medical Center Institutional Review Board; East Carolina University Office for Human Research Integrity  Brody School of Medicine 4N-70 Mail Stop 682  600 Moye Boulevard  Greenville, North Carolina 27834  USA |
|  |  |
| Fessel, Walford, MD (1605) | Kaiser Permanente Norther California Institutional Review Board  1800 Harrison Street  16^th^ Floor  Oakland, California 94612-3431  USA |
| Grant, Philip, MD (8865) | Administrative Panel on Human Subjects in Medical Research  3000 El Camino Real  Five Palo Alto Square, 4^th^ Floor  Palo Alto, California 94306  USA |
|  |  |
|  |  |
| Huhn, Gregory, MD (3612)  Jain, Mamta, MD (1691)  Jayaweera, Dushyantha, MD (1692)  Luketic, Velimir, MD (5751) | Cook County Bureau of Health Services Institutional Review Board  627 South Wood Street  Chicago, Illinois 60612  USA  University of Texas Southwestern Medical Center Institutional Review Board of Dallas  5323 Harry Hines Boulevard, BL9, 100  Dallas, Texas 75390  USA  University Of Miami  1400 NW 10th Avenue  Dominion Towers  Suite 120  Miami, Florida 33136  USA  McGuire Institutional Review Board  1201 Broad Rock Boulevard  Room 3C-126  Richmond, Virginia 23249  USA |
|  |  |
| Marks, Kristen, MD (8056) | Weill Cornell Medical College Institutional Review Board  1300 York Avenue  Box-89  New York, New York 10065  USA |
| Mounzer, Karam, MD (1961)  Naggie, Susanna, MD (6613)  Saag, Michael, MD (446)  Siegel, Marc, MD (8889) | Philadelphia FIGHT Institutional Review Board  1233 Locust Street  Fifth Floor  Philadelphia, Pennsylvania 19107  USA  Duke University Health System Institutional Review Board  Hock Plaza, Suite 405  2424 Erwin Road  Durham, North Carolina 27705  USA  Western Institutional Review Board  1019 39^th^ Avenue SE  Puyallup, Washington 98374  USA  Western Institutional Review Board  1019 39^th^ Avenue SE  Suite 120  Puyallup, Washington 98374-2115  USA |
|  |  |
|  |  |
| Slim, Jihad, MD (0310) | Saint Michael's Medical Center IRB  111 Central Avenue  Newark, New Jersey 07102  USA |
| Sulkowski, Mark, MD (1516) | John Hopkins Medicine Institutional Review Board  Reed Hall B-130  1620 McElderry Street  Baltimore, Maryland 21205-1911  USA |
|  |  |
| Thompson, Melanie, MD (0255), | Aids Research Consortium of Atlanta Institutional Review Board  440 Ralph McGill Boulevard  Atlanta, Georgia 30312  USA |
| Workowski, Kimberly, MD (2140)  Yozviak, Joseph, MD (12200) | Western Institutional Review Board  1019 39^th^ Avenue SE  Suite 120  Puyallup, Washington 98374-2115  USA  Lehigh Valley Health Network/Institutional Review Board/Research Participation Office  707 Hamilton Street  Allentown, Pennsylvania 18101  USA |
