# Supplementary material for: HIV/HCV therapy with ledipasvir/sofosbuvir after randomized switch to emtricitabine-tenofovir alafenamide-based single-tablet regimens
Source: PLoS One. 2020 Jan 29;15(1):e0224875. doi: 10.1371/journal.pone.0224875 (PMC6988963; doi:10.1371/journal.pone.0224875)
Supplement: S1 Text — (DOCX) [file pone.0224875.s002.docx]

**Supplementary Material**

Supplementary Material has been provided by the authors to give readers additional information about their work.

Supplement to: **HIV/HCV therapy with ledipasvir/sofosbuvir after randomized switch to emtricitabine-tenofovir alafenamide-based single-tablet regimens**

Gregory D. Huhn, Moti Ramgopal, Mamta K. Jain, Federico Hinestrosa, David M. Asmuth, Jihad Slim, Deborah Goldstein, Shauna Applin, Julie H. Ryu, Shuping Jiang, Stephanie Cox, Moupali Das, Thai Nguyen-Cleary, David Piontkowsky, Bill Guyer, Lorenzo Rossaro, and Richard H. Haubrich

# S1 Text. Full inclusion criteria.

***Inclusion criteria (all must be met for inclusion in study)***

1. The ability to understand and sign a written informed consent form, obtained prior to study procedure initiation.
2. Age ≥18 years.
3. Current antiretroviral (ARV) regimen comprising two nucleoside reverse transcriptase inhibitors (NRTI) and a third agent (Table), unchanged for 6 months prior to screening. For participants with three or more prior ARV regimens, regimen histories were approved individually. If the current regimen is a protease inhibitor (PI)/ritonavir regimen, the previous regimen cannot contain a non-nucleoside reverse transcriptase inhibitor (NNRTI) or integrase strand transfer inhibitor (INSTI). Participants who changed from tenofovir disoproxil fumarate (TDF) to tenofovir alafenamide (TAF) fewer than 6 months prior to screening were eligible if the TDF/TAF change was the only change to the regimen.

Table. Allowable ARV agents of pre-existing HIV regimen.

| **Antiretroviral class** | **Agents** |
| --- | --- |
| Boosted PI | ATV+COBI (or ATV/COBI FDC), DRV+COBI (or DRV/COBI FDC), DRV+RTV, lopinavir/RTV, ATV+RTV, FPV+RTV, FPV+COBI, SQV+RTV, SQV+COBI, IDV+RTV, IDV+COBI, ATV (no booster) |
| NNRTI | EFV, etravirine, nevirapine, rilpivirine |
| NRTI | Emtricitabine, TDF, TAF, abacavir, lamivudine, zidovudine |
| INSTI | Dolutegravir, raltegravir, EVG/COBI, EVG+RTV |

ATV, atazanavir; COBI, cobicistat; DRV, darunavir; EFV, efavirenz; EVG, elvitegravir; FDC, fixed-dose combination; FPV, fosamprenavir; INSTI, integrase strand transfer inhibitor; IDV, indinavir; NNRTI, non-nucleoside reverse transcriptase inhibitor; NRTI, nucleoside reverse transcriptase inhibitor; PI, protease inhibitor; RTV, ritonavir; SQV, saquinavir; TAF, tenofovir alafenamide; TDF, tenofovir disoproxil fumarate.

1. Documented plasma HIV-1 RNA levels <50 copies/mL (or undetectable HIV-1 RNA level if the limit of detection is ≥50 copies/mL in the local assay) for ≥6 months preceding the Screening visit. After reaching HIV-1 RNA <50 copies/mL, single values (“blips”) of HIV-1 RNA ≥50 copies/mL followed by re-suppression were allowed.
2. No history of HIV virologic failure.
3. Plasma HIV-1 RNA level <50 copies/mL at the Screening visit.
4. No documented resistance to any of the HIV study agents at any time in the past, including but not limited to the reverse transcriptase resistance mutations K65R, K70E, K101E/P, E138A/G/K/R/Q, V179L, Y181C/I/V, M184V/I, Y188L, H221Y, F227C, M230I/L, the combination of K103N+L100I, or three or more thymidine analog-associated mutations (TAMs) that include M41L or L210W (TAMs are M41L, D67N, K70R, L210W, T215Y/F, K219Q/E/N/R). If a historical genotype prior to first ARV is not available or participant has three or more prior ARV regimens, participant will have proviral genotype analysis for archived resistance prior to Day 1, and proviral reports approved.
5. Chronic HCV infection (≥6 months) documented by prior medical history.
6. HCV genotype 1 at screening as determined by the Central Laboratory. Any non-definitive genotype results will exclude the participant from study participation.
7. Classification as one of the following criteria:
   1. Without cirrhosis and no prior HCV treatment (treatment-naïve, defined as having never been exposed to approved or investigational HCV-specific direct-acting antiviral agents or prior treatment of HCV with interferon [IFN] and ribavirin [RBV])
   2. Without cirrhosis and have received HCV treatment only with IFN+/-RBV+/-HCV PI (treatment-experienced, defined as prior treatment failure to a regimen containing IFN either with or without RBV that was completed at least 8 weeks prior to Day 1. The participant’s medical records must include sufficient detail of prior treatment experience for categorization, as either:
      1. Non-Responder: Participant did not achieve undetectable HCV RNA levels while on treatment, or
      2. Relapse/Breakthrough: Participant achieved undetectable HCV RNA levels during treatment or within 4 weeks of the end of treatment but did not achieve sustained virologic response (SVR), or
      3. HCV Treatment-Intolerant: Participants who discontinued HCV treatment of a regimen containing IFN (either with or without RBV) due to toxicity.
   3. Compensated cirrhosis and no prior HCV treatment (treatment-naïve).
8. Cirrhosis determination
   1. Presence of cirrhosis is defined as any one of the following:
      1. FibroTest® score >0.75 AND an aspartate aminotransferase (AST):platelet ratio index (APRI) >2 during screening
      2. Transient elastography (FibroScan®) with a result of >12.5 kPa
      3. Liver biopsy showing cirrhosis (e.g., Metavir score 4 or Ishak score ≥5).
   2. Absence of cirrhosis is defined as any one of the following:
      1. FibroTest® score ≤0.48 AND APRI ≤1 performed during screening
      2. FibroScan® with a result of ≤12.5 kPa within 6 months of Day 1
      3. Liver biopsy within 2 years of screening showing absence of cirrhosis.

In the absence of a definitive diagnosis of presence or absence of cirrhosis by Fibrotest®/APRI, a liver biopsy or FibroScan® is required. Liver biopsy results are considered definitive.

1. Liver imaging ≤6 months prior to Day 1 is required in cirrhotic participants to exclude hepatocellular carcinoma.
2. HCV RNA ≥104 IU/mL at the Screening visit.
3. Normal electrocardiogram (ECG) (or if abnormal, determined by the Investigator to be not clinically significant).
4. Adequate renal function: estimated glomerular filtration rate (eGFR) ≥30 mL/min according to the Cockcroft–Gault formula (eGFR_CG_) for creatinine clearance (CL_cr_) [1].
   1. Male: (140 – age in years) × (weight in kg) = CL_cr_ (mL/min)

72 × (serum creatinine in mg/dL)

- 1. Female: (140 – age in years) × (weight in kg) × 0.85 = CL_cr_ (mL/min)

72 × (serum creatinine in mg/dL)

1. Hepatic transaminases (AST and alanine aminotransferase [ALT]) ≤5 × upper limit of normal (ULN).
2. Total bilirubin ≤1.5 mg/dL, and direct bilirubin ≤1.5 × ULN (participants with documented Gilbert’s syndrome or with atazanavir-associated hyperbilirubinemia may have total bilirubin <5 × ULN as long as direct bilirubin is ≤1.5 × ULN).
3. Adequate hematologic function:

• Absolute neutrophil count ≥1000/mm^3^

• Platelets ≥50,000/mm^3^

• Hemoglobin ≥8.5 g/dL.

1. Albumin ≥3.0 g/dL.
2. International Normalized Ratio (INR) ≤1.5 × ULN unless participant has known hemophilia or is stable on an anticoagulant regimen affecting INR.
3. A female participant is eligible if it is confirmed that she is:
   1. Not pregnant or nursing
   2. Of non-childbearing potential (i.e., women who have had a hysterectomy, have had both ovaries removed or medically documented ovarian failure, or are postmenopausal women ≥54 years of age with cessation for ≥12 months of previously occurring menses), or
   3. Of childbearing potential (following the initiation of puberty [Tanner stage 2] until post-menopausal, unless permanently sterile or with medically documented ovarian failure) and agrees to either
      1. utilize protocol-specified contraceptive methods (consistent, correct use of one of intrauterine device, tubal sterilization, essure micro-insert system, diaphragm with spermicide and a male condom without spermicide, cervical cap with spermicide and male condom without spermicide, or vasectomy in the sole male partner; or continue hormonal contraceptive method and use a single barrier method) or
      2. be non-heterosexually active or
      3. practice sexual abstinence from screening throughout the duration of study treatment and for 30 days following the last study drug dose.
4. Female participants must refrain from egg donation and *in vitro* fertilization during treatment and for 30 days following the last study drug dose.
5. Male participants must agree to utilize a protocol-approved method of contraception (condoms without spermicide) during heterosexual intercourse of reproductive potential or be non-heterosexually active or practice sexual abstinence from first dose throughout the study period and for 30 days following the last study drug dose.
6. Male participants must agree to refrain from sperm donation from first dose until at least 30 days after the last study drug dose.

**Reference**

1. Cockcroft DW, Gault MH. Prediction of creatinine clearance from serum creatinine. Nephron. 1976; 16(1): 31-41.
