# Supplementary material for: HIV/HCV therapy with ledipasvir/sofosbuvir after randomized switch to emtricitabine-tenofovir alafenamide-based single-tablet regimens
Source: PLoS One. 2020 Jan 29;15(1):e0224875. doi: 10.1371/journal.pone.0224875 (PMC6988963; doi:10.1371/journal.pone.0224875)
Supplement: S2 Text — (DOCX) [file pone.0224875.s003.docx]

**Supplementary Material**

Supplementary Material has been provided by the authors to give readers additional information about their work.

Supplement to: **HIV/HCV therapy with ledipasvir/sofosbuvir after randomized switch to emtricitabine-tenofovir alafenamide-based single-tablet regimens**

Gregory D. Huhn, Moti Ramgopal, Mamta K. Jain, Federico Hinestrosa, David M. Asmuth, Jihad Slim, Deborah Goldstein, Shauna Applin, Julie H. Ryu, Shuping Jiang, Stephanie Cox, Moupali Das, Thai Nguyen-Cleary, David Piontkowsky, Bill Guyer, Lorenzo Rossaro, and Richard H. Haubrich

# S2 Text. Full exclusion criteria.

1. A new AIDS-defining condition diagnosed <30 days prior to the Screening visit (as defined by [1] except CD4 cell count and/or percentage criteria).
2. Prior HCV treatments with direct-acting antivirals except boceprevir, telaprevir, and simeprevir in combination with IFN +/- RBV.
3. Chronic liver disease of a non-HCV etiology (e.g., hemochromatosis, Wilson’s disease, α-1 antitrypsin deficiency, cholangitis).
4. History or current decompensation of liver disease; varices at low risk of bleeding are allowed.
5. Positive hepatitis B surface antigen or hepatitis B virus DNA.
6. Chronic use of systemically administered immunosuppressive agents such as prednisone equivalent to >10 mg/day.
7. Females who are breastfeeding.
8. Positive serum pregnancy test.
9. Have an implanted defibrillator or pacemaker.
10. Current alcohol or substance use judged to potentially interfere with study compliance.
11. A history of malignancy within the 5 years prior to screening, or ongoing malignancy other than cutaneous Kaposi's sarcoma (KS), basal cell carcinoma, or resected, non-invasive cutaneous squamous carcinoma. Participants with cutaneous KS are eligible but must not have received any systemic therapy for KS within 42 days of Day 1 visit and must not be anticipated to require systemic therapy during the study.
12. Active, serious infections (other than HIV-1 and HCV infections) requiring parenteral antibiotic or antifungal therapy within 42 days prior to Day 1.
13. Gastrointestinal disorder or post-operative condition that could interfere with the absorption of study drugs.
14. Solid organ transplantation.
15. Significant pulmonary disease or significant cardiac disease.
16. Any other clinical condition or prior therapy that, in the opinion of the Investigator, would make the participant unsuitable for the study or unable to comply with dosing requirements.
17. Participation in any other clinical trial (including observational trials) without prior approval from the Sponsor.
18. Known hypersensitivity to the study drugs, the metabolites, or formulation excipients.
19. Ongoing therapy with any of the medications in the Table below, including drugs not to be used due to the potential for interaction with HIV or HCV study drugs.

Table. Disallowed agents.

| **Drug class** | **Agents disallowed^a^** |
| --- | --- |
| Acid-reducing agents | Proton pump inhibitors, such as esomeprazole, lansoprazole, omeprazole, pantoprazole, rabeprazole |
| Alpha-adrenergic receptor antagonists | Alfuzosin |
| Antiarrhythmics | Amiodarone^c^, quinidine |
| Anticonvulsants^b^ | Phenobarbital, phenytoin, carbamazepine, oxcarbazepine |
| Antimycobacterials^b^ | Rifampin, rifapentine, rifabutin |
| Calcium channel blockers | Bepridil |
| Corticosteroids: systemic | Dexamethasone (more than a single dose) |
| Ergot derivatives | Ergotamine, ergonovine, dihydroergotamine, methylergotamine, ergometrine |
| Gastrointestinal motility agents | Cisapride |
| Herbal/natural supplements^b^ | St. John’s Wort, Echinacea, milk thistle (silymarin), Chinese herb sho-saiko-to (or Xiao-Shai-Hu-Tang) |
| HMG-CoA reductase inhibitors | Simvastatin, lovastatin, rosuvastatin^d^ |
| Inhaled beta-agonist | Salmeterol |
| Neuroleptics | Pimozide |
| Phosphodiesterase-5 inhibitors | Sildenafil (for pulmonary arterial hypertension) |
| Sedatives/hypnotics | Orally-administered midazolam, triazolam |

^a^Administration of any disallowed agents must be discontinued at least 42 days prior to the Day 1 visit and for the duration of the study, with the exception of amiodarone (see footnote c).

^b^May result in a decrease in the concentrations of all HIV and HCV study drugs.

^c^May result in symptomatic bradycardia (mechanism unknown). The use of amiodarone is prohibited from 60 days prior to the Day 1 visit and for the duration of the study.

^d^Use with ledipasvir/sofosbuvir may result in an increase in the concentration of rosuvastatin, which is associated with increased risk of myopathy including rhabdomyolysis.

HMG-CoA, 3-hydroxy-3-methyl-glutaryl-cS2oenzyme A.

**Reference**

1. Selik RM, Mokotoff ED, Branson B, et al. Revised surveillance case definition for HIV infection--United States, 2014. MMWR Recomm Rep. 2014; 63(Rr-03): 1-10.
