# Supplementary material for: HIV/HCV therapy with ledipasvir/sofosbuvir after randomized switch to emtricitabine-tenofovir alafenamide-based single-tablet regimens
Source: PLoS One. 2020 Jan 29;15(1):e0224875. doi: 10.1371/journal.pone.0224875 (PMC6988963; doi:10.1371/journal.pone.0224875)
Supplement: S3 Text — (DOCX) [file pone.0224875.s004.docx]

**Supplementary Material**

Supplementary Material has been provided by the authors to give readers additional information about their work.

Supplement to: **HIV/HCV therapy with ledipasvir/sofosbuvir after randomized switch to emtricitabine-tenofovir alafenamide-based single-tablet regimens**

Gregory D. Huhn, Moti Ramgopal, Mamta K. Jain, Federico Hinestrosa, David M. Asmuth, Jihad Slim, Deborah Goldstein, Shauna Applin, Julie H. Ryu, Shuping Jiang, Stephanie Cox, Moupali Das, Thai Nguyen-Cleary, David Piontkowsky, Bill Guyer, Lorenzo Rossaro, and Richard H. Haubrich

# S3 Text. Assessments performed at each study visit.

Full details of assessments performed at each study visit are summarized in the table below. Plasma HIV-1 RNA levels and CD4+ T-cell counts were measured at every study visit (Screening, Day 1, Week [W] 4, W6 [not CD4+ T-cell count], W8, W12, W16, and W20, Post-HCV W4 and W12, 30-Day Follow-Up and Early Study Drug Discontinuation [ESDD]). HIV-1 RNA was quantified by COBAS® TaqMan® HIV RNA Test, v2.0 (Roche Molecular Diagnostics, Pleasanton, CA). Plasma HCV RNA was evaluated at screening, baseline, W8, and all visits after study Part 1 by COBAS® TaqMan® HCV Quantitative Test, v2.0 (Roche Molecular Diagnostics, Pleasanton, CA). Further HCV analyses (genotype and subtype, IL28B genotype, anti-HCV antibody serology, and cirrhosis determination) were conducted at the Screening visit. Safety and tolerability were assessed by serum chemistries, hematology, coagulation, and fasting metabolic assessments, blood analysis for bone safety and inflammation markers, urinalysis and eGFR_CG_ for renal safety, and adverse event (AE) monitoring throughout the study (except W6 laboratory-only visit). AEs were coded using the Medical Dictionary for Regulatory Activities, v20.0. All blood and plasma determinations were done at a central laboratory.

| **Procedure** | **Screening^a^** | **Day 1^b^** | **End of Week^c^**  **Part 1**  **On Treatment** | | **End of Week^c^ – Part 2** | | | | | | **30-Day Follow-Up^c^** | **ESDD^d^** |
| --- | --- | --- | --- | --- | --- | --- | --- | --- | --- | --- | --- | --- |
|  |  |  |  |  | **On Treatment** | | | | **Post-HCV Treatment** | |  |  |
|  |  |  | **4** | **6** | **8** | **12** | **16** | **20** | **4** | **12** |  |  |
| Written informed consent | X |  |  |  |  |  |  |  |  |  |  |  |
| Medical history | X |  |  |  |  |  |  |  |  |  |  |  |
| Liver cirrhosis determination | X |  |  |  |  |  |  |  |  |  |  |  |
| Concomitant medications | X | X | X |  | X | X | X | X | X | X | X | X |
| Adverse events | X | X | X |  | X | X | X | X | X | X | X | X |
| Complete physical examination | X | X |  |  |  |  |  |  |  | X |  | X |
| Symptom-directed physical examination^e^ |  |  | X |  | X | X | X | X | X |  | X |  |
| Vital signs & weight | X | X | X |  | X | X | X | X | X | X | X | X |
| Height | X |  |  |  |  |  |  |  |  |  |  |  |
| 12-lead ECG (performed supine) | X |  |  |  |  |  |  |  |  |  |  | X |
| Urinalysis & urine chemistry | X | X | X |  | X | X | X | X | X | X | X | X |
| Urine storage sample |  | X | X |  | X | X | X | X | X | X |  | X |
| Urine pregnancy test^f^ |  | X | X |  | X | X | X | X | X | X | X | X |
| Serum pregnancy test^f^ | X |  |  |  |  |  |  |  |  |  |  |  |
| Chemistry profile^g^ | X | X | X |  | X | X | X | X | X | X | X | X |
| Hematology profile^h^ | X | X | X |  | X | X | X | X | X | X | X | X |
| Coagulation assessments^i^ | X | X | X |  | X | X | X | X | X | X |  |  |
| Metabolic assessments^j^ |  | X |  |  | X |  |  | X |  | X |  |  |
| CD4+ T-cell count | X | X | X |  | X | X | X | X | X | X | X | X |
| Plasma HIV-1 RNA | X | X | X | X | X | X | X | X | X | X | X | X |
| HIV-1 proviral genotype^k^ | X |  |  |  |  |  |  |  |  |  |  |  |
| HIV-1 genotype/phenotype^l^ |  |  |  |  |  |  |  |  |  |  |  | X |
| Plasma HCV RNA | X | X |  |  | X | X | X | X | X | X | X | X |
| HCV genotype & subtype | X |  |  |  |  |  |  |  |  |  |  |  |
| HCV IL28B genotype | X |  |  |  |  |  |  |  |  |  |  |  |
| HIV, HBV, & HCV serologies^m^ | X |  |  |  |  |  |  |  |  |  |  |  |
| Evaluations of bone & renal safety, inflammation, and platelet & coagulation function^n^ |  | X | X |  | X | X |  | X |  | X |  | X |
| Plasma storage sample^o^ |  | X | X | X | X | X | X | X | X | X |  | X |
| Serum storage sample^o^ |  | X | X |  | X | X | X | X | X | X |  | X |
| Optional plasma and serum storage sample^p^ |  | X | X |  | X |  |  | X |  | X |  | X |
| Estimated GFR_CG_^q^ | X | X | X |  | X | X | X | X | X | X |  | X |
| Population pharmacokinetics^r^ |  |  | X |  | X | X | X | X |  |  |  |  |
| Health-related questionnaires^s^ |  | X |  |  | X |  |  | X |  | X |  | X |
| Randomization |  | X |  |  |  |  |  |  |  |  |  |  |
| Study drug dispensation & accountability^t^ |  | X | X |  | X | X | X | X | X | X |  | X^u^ |

^a^Screening evaluations to be completed within 42 days prior to the Day 1 visit.

^b^Participants dispensed HIV study drugs at the Day 1 visit; initiation of treatment with study drugs must take place within 24 hours after Day 1 visit.

^c^All study visits are to be scheduled relative to the Day 1 visit date. Visit windows are ± 2 days of the protocol-specified date until Post-HCV treatment Week 4 visit, ± 4 days of the protocol-specified date at Post-HCV treatment Week 12, and ± 6 days of the protocol-specified date for the 30-Day Follow-Up visit.

^d^ESDD visit to occur within 72 hours of last dose of study drug. Participants discontinuing during Part 1 do not continue to Part 2 of the study but attend the ESDD and 30-Day Follow-Up visit. During Part 2, participants discontinuing HCV study drugs only attend the ESDD visit, continue to receive HIV study drugs, and remain in the study for Post-HCV Week 4 and 12 visits. During Part 2, participants discontinuing HIV study drug only attend the ESDD visit, may change HIV regimen, and continue receiving HCV study drugs (if co-administration is possible with new HIV regimen) and remain in the study for Post-HCV Week 4 and 12 visits. Participants discontinuing both HIV and HCV study drugs attend the ESDD visit and remain in the study for Post-HCV Week 4 and 12 visits. Any evaluations showing abnormal results with a possible or probable causal relationship with the study drugs to be repeated weekly (or as often as deemed prudent by the Investigator) until the abnormality is resolved, returns to baseline, or is otherwise explained.

^e^Symptom-directed physical examination performed as needed.

^f^For females of childbearing potential only (defined in the inclusion criteria, Supplemental Digital Content 1). Positive urine pregnancy tests confirmed with a serum test.

^g^Chemistry profile: alkaline phosphatase, aspartate aminotransferase, alanine aminotransferase, total bilirubin, total protein, albumin, bicarbonate, blood urea nitrogen, calcium, chloride, creatinine, glucose, phosphorus, magnesium, potassium, sodium, and uric acid. At visits in which metabolic assessments are performed, analyses of glucose will be done as part of the fasting metabolic assessments and not as part of the chemistry profile.

^h^Complete blood count with differential and platelet count.

^i^Coagulation assessments: International Normalized Ratio, prothrombin time, and activated partial thromboplastin time.

^j^Metabolic assessments (collected fasted, no food or drink, except water, at least 8 hours prior to blood collection): glucose and lipid panel (total cholesterol, high-density lipoprotein, direct low-density lipoprotein, and triglycerides).

^k^If a historical genotype report prior to first antiretroviral (ARV) treatment is not available, or participant has three or more prior ARV regimens, whole blood sample for proviral genotype analysis of archived resistance.

^l^HIV-1 genotype/phenotype resistance testing for participants with unconfirmed virologic rebound with HIV-1 RNA value ≥400 copies/mL.

^m^Serology testing includes HIV and HCV antibody, and hepatitis B virus (HBV) core antibody (HBcAb), surface antibody (HBsAb), surface antigen (HBsAg), e-antibody (HBeAg), and HBV DNA.

^n^Blood for bone safety, parathyroid, and serum OH-25 vitamin D. Inflammation may include cystatin-C, IL-6, hs-CRP, sCD14, sCD163, sTNF-1R, and Lp-PLA2. Platelet and coagulation function may include soluble glycoprotein VI, P-selectin, soluble CD40 ligand, and D-dimer will be collected. Urine for renal safety, including retinol binding protein, beta-2-microglobulin, urine albumin, and urine protein will be collected. Samples will be collected fasted. If the participant has not fasted prior to the visit, the visit may proceed, but the participant must return within 72 hours in a fasted state.

^o^Plasma storage samples for safety, virology, and/or pharmacokinetics testing. Serum storage samples for possible additional clinical testing.

^p^Optional plasma and serum samples for exploratory assessments (for participants who provide additional consent).

^q^Estimated GFR_CG_ for creatinine clearance (described in inclusion criteria, Supplemental Digital Content 1).

^r^Two blood samples will be collected: 1) the pre-dose sample should be drawn prior to observed study drug dosing; 2) the post-dose sample should be drawn between 15 mins and 4 hours post-dose.

^s^Health-related questionnaires: Adherence for HIV (visual analog scale), HIV Treatment Satisfaction Questionnaire – Status version (HIVTSQs, at Day 1 only), HIVTSQc – Change version (at all other visits), Medical Outcomes Short Form-36 (SF-36), Functional Assessment of Chronic Illness Therapy-Fatigue scale (FACIT-F), Chronic Liver Disease Questionnaire (CLDQ-HCV), and Work Productivity and Activity Impairment Questionnaire: Hepatitis C v2.0 (WPAI: Hepatitis C).

^t^Part 1: HIV study drugs are dispensed after all Day 1 assessments and randomization are completed, and through the duration of the study. Part 2: After determination of HIV suppression (<50 copies/mL) based on the Week 6 HIV-1 RNA value and tolerability of HIV study drugs, ledipasvir/sofosbuvir will be dispensed at Weeks 8, 12, and 16 only. Post-HCV Treatment Week 12: drug accountability only; study drug will not be dispensed at this visit.

^u^If participants discontinue during Part 1, only drug accountability will be performed; study drug will not be dispensed at this visit. In Part 2, if participant discontinues ledipasvir/sofosbuvir but continues with HIV study drug, HIV study drug will be dispensed until the last study visit. If participant discontinues HIV study drug but continues with ledipasvir/sofosbuvir, ledipasvir/sofosbuvir will be dispensed. If participant discontinues both HIV and HCV study drugs, only drug accountability will be performed; no drug will be dispensed at this visit.

ECG, electrocardiogram; ESDD, Early Study Drug Discontinuation; GRF_CG_, glomerular filtration rate calculated using the Cockcroft–Gault equation
