# Supplementary material for: HIV/HCV therapy with ledipasvir/sofosbuvir after randomized switch to emtricitabine-tenofovir alafenamide-based single-tablet regimens
Source: PLoS One. 2020 Jan 29;15(1):e0224875. doi: 10.1371/journal.pone.0224875 (PMC6988963; doi:10.1371/journal.pone.0224875)
Supplement: S2 File — (PDF) [file pone.0224875.s006.pdf]

CLINICAL STUDY PROTOCOL

**Study Title:**	A Phase 3b Randomized, Open-label, Controlled Study of the Efficacy, Safety and Tolerability of 12 Weeks of Ledipasvir/Sofosbuvir (LDV/SOF) Treatment for HIV/HCV Co-infected Subjects who Switch to Elvitegravir/Cobicistat/Emtricitabine/Tenofovir Alafenamide (E/C/F/TAF) or Emtricitabine/Rilpivirine/Tenofovir Alafenamide (F/R/TAF) prior to LDV/SOF HCV Treatment, the HIV/HCV Co-STARs study (Co-infection treatment with Single Tablet Antiviral Regimens)	
**Sponsor:**	Gilead Sciences, Inc.<br>333 Lakeside Drive<br>Foster City, CA 94404	
**IND Number:**<br>**EudraCT Number:**<br>**Clinical Trials.gov Identifier:**	123098<br>2014-004545-27<br>NCT02707601	
**Indication:**	HIV-1 Infection	
**Protocol ID:**	GS-US-366-1992	
**Gilead Clinical Program Manager:**	Name:<br>Telephone:<br>Fax:	PPD<br>PPD<br>PPD
**Gilead Co-Medical Monitor:**	Name:<br>Telephone:<br>Mobile:	Richard Haubrich, MD<br>PPD<br>PPD
**Gilead Co-Medical Monitor:**	Name:<br>Telephone:<br>Mobile:	Thai Nguyen-Cleary, MD<br>PPD<br>PPD
**Protocol Version/Date:**	Original:<br>Amendment 1:	18 December 2015<br>27 May 2016

CCI

TABLE OF CONTENTS

TABLE OF CONTENTS 2
LIST OF IN-TEXT TABLES 5
LIST OF IN-TEXT FIGURES 5
PROTOCOL SYNOPSIS 6
GLOSSARY OF ABBREVIATIONS AND DEFINITION OF TERMS 13
1. INTRODUCTION 17
1.1. Background 17
1.2. Tenofovir Alafenamide (TAF, GS-7340) 19
1.2.1. General Information 19
1.2.2. Clinical Trials of Elvitegravir/Cobicistat/Emtricitabine/Tenofovir Alafenamide (E/C/F/TAF, Genvoya®) 19
1.2.3. Clinical Trials of Emtricitabine/Rilpivirine/Tenofovir Alafenamide (F/R/TAF, Odefsey®) Fixed Dose Combination 21
1.3. Ledipasvir/Sofosbuvir (LDV/SOF, Harvoni®) Fixed Dose Combination 22
1.3.1. General Information 22
1.3.2. Clinical Trials of Ledipasvir/Sofosbuvir (LDV/SOF, Harvoni®) Relevant to the Proposed Study 22
1.4. Rationale for This Study 27
1.5. Risk/Benefit Assessment for the Study 28
1.6. Compliance 29
2. OBJECTIVES 30
3. STUDY DESIGN 31
3.1. Endpoints 31
3.2. Study Design 31
3.3. Study Treatments 31
3.4. Duration of Treatment 32
3.5. Samples for Optional Exploratory Assessments 33
4. SUBJECT POPULATION 34
4.1. Number of Subjects and Subject Selection 34
4.2. Inclusion Criteria 34
4.3. Exclusion Criteria 37
5. INVESTIGATIONAL MEDICINAL PRODUCTS 40
5.1. Randomization 40
5.2. Description and Handling of E/C/F/TAF, F/R/TAF, and LDV/SOF 40
5.2.1. Formulation 40
5.2.2. Packaging and Labeling 41
5.2.3. Storage and Handling 42
5.3. Dosage and Administration of E/C/F/TAF, F/R/TAF, and LDV/SOF 42
5.4. Prior and Concomitant Medications 42
5.5. Accountability for Investigational Medicinal Product (IMP) 48
5.5.1. Investigational Medicinal Product Return or Disposal 48
6. STUDY PROCEDURES 49
6.1. Subject Enrollment and Treatment Assignment 49
6.2. Pretreatment Assessments 49

6.2.1. Screening Visit 49
6.2.2. Day 1 Assessments 51
6.3. Randomization 53
6.4. Treatment Assessments 53
6.4.1. HIV and HCV Treatment Visits (Weeks 4, 6, 8, 12, 16, and 20) 53
6.4.2. HIV Treatment and Post-HCV Treatment Assessments (Post-HCV Treatment Weeks 4 and 12) 56
6.5. Post-treatment Assessments 58
6.5.1. Early Study Drug Discontinuation (ESDD) Visit 58
6.5.2. 30-Day Follow-Up Visit 60
6.6. Assessments for Premature Discontinuation from Study 61
6.7. Criteria for Discontinuation of Study Treatment 61
6.8. Other Assessments 62
6.8.1. Blood and Urine Storage 62
6.8.2. Bone Safety and Inflammation 62
6.8.3. Markers of Platelet Function and Coagulation 62
6.8.4. Markers of Renal Tubular Function 62
6.9. HIV Virologic Failure 63
6.9.1. Management of HIV Virologic Rebound 63
6.9.2. Subjects with ≥ 400 copies/mL of HIV-1 at the Last Study Visit 65
6.10. HCV Virologic Response-Based Treatment Stopping Criteria 66
6.11. End of Study 66
6.12. Post Study Care 66

7. ADVERSE EVENTS AND TOXICITY MANAGEMENT 67

7.1. Definitions of Adverse Events, Adverse Reactions, and Serious Adverse Events 67
7.1.1. Adverse Events 67
7.1.2. Serious Adverse Events 67
7.1.3. Clinical Laboratory Abnormalities and Other Abnormal Assessments as Adverse Events or Serious Adverse Events 68
7.2. Assessment of Adverse Events and Serious Adverse Events 68
7.2.1. Assessment of Causality for Study Drugs and Procedures 68
7.2.2. Assessment of Severity 69
7.3. Investigator Requirements and Instructions for Reporting Adverse Events and Serious Adverse Events to Gilead 69
7.4. Gilead Reporting Requirements 70
7.5. Toxicity Management 71
7.5.1. Grades 1 and 2 Laboratory Abnormality or Clinical Event 71
7.5.2. Grade 3 Laboratory Abnormality or Clinical Event 72
7.5.3. Grade 4 Laboratory Abnormality or Clinical Event 72
7.5.4. Management of Potential Nephrotoxicity 72
7.6. Stopping Rules for HCV Treatment 73
7.7. Special Situations Reports 73
7.7.1. Definitions of Special Situations 73
7.7.2. Instructions for Reporting Special Situations 74

8. STATISTICAL CONSIDERATIONS 76

8.1. Analysis Objectives and Endpoints 76
8.1.1. Analysis Objectives 76
8.1.2. Primary Endpoint 76
8.1.3. Secondary Endpoint 76
8.1.4. Other Endpoints of Interest 77
8.2. Analysis Conventions 77
8.2.1. Analysis Sets 77

8.3. Demographic Data and Baseline Characteristics 77
8.4. Efficacy Analysis 78
8.4.1. Primary Analysis 78
8.4.2. Secondary Analyses 78
8.5. Safety Analysis 79
8.5.1. Extent of Exposure 79
8.5.2. Adverse Events 79
8.5.3. Laboratory Evaluations 80
8.5.4. Other Safety Evaluations 80
8.6. Pharmacokinetic Analysis 80
8.7. Biomarker Analysis 80
8.8. Sample Size 80

9. RESPONSIBILITIES 81

9.1. Investigator Responsibilities 81
9.1.1. Good Clinical Practice 81
9.1.2. Institutional Review Board (IRB)/Independent Ethics Committee (IEC) Review and Approval 81
9.1.3. Informed Consent 81
9.1.4. Confidentiality 82
9.1.5. Study Files and Retention of Records 82
9.1.6. Case Report Forms 83
9.1.7. Investigational Medicinal Product Accountability and Return 84
9.1.8. Inspections 84
9.1.9. Protocol Compliance 84
9.2. Sponsor Responsibilities 84
9.2.1. Protocol Modifications 84
9.2.2. Study Report and Publications 85
9.3. Joint Investigator/Sponsor Responsibilities 85
9.3.1. Payment Reporting 85
9.3.2. Access to Information for Monitoring 85
9.3.3. Access to Information for Auditing or Inspections 86
9.3.4. Study Discontinuation 86

10. REFERENCES 87

11. APPENDICES 91

Appendix 1. Investigator Signature Page 92
Appendix 2. Study Procedures Table 93
Appendix 3. Management of Clinical and Laboratory Adverse Events (HIV ARV Agents only) 96
Appendix 4. GSI Grading Scale for Severity of Adverse Events and Laboratory Abnormalities 97
Appendix 5. Pregnancy Precautions, Definition for Female of Childbearing Potential, and Contraceptive Requirements 120
Appendix 6. CDC Classification Systems, AIDS-Indicator Conditions 123

LIST OF IN-TEXT TABLES

Table 1-1. Combined Analysis from Phase III ION Studies 24
Table 1-2. Study 1: F/R/TAF and LDV/SOF 25
Table 1-3. Study 2: E/C/F/TAF and LDV/SOF PK Parameter 26
Table 4-1. Allowable ARV Agents of Pre-Existing HIV Regimen 34
Table 4-2. Disallowed Agents[a] 38
Table 5-1. Prior and Concomitant Medications – E/C/F/TAF + LDV/SOF 43
Table 5-2. Prior and Concomitant Medications – F/R/TAF + LDV/SOF 47

LIST OF IN-TEXT FIGURES

Figure 3-1. Study Schema 32
Figure 6-1. HIV Virologic Rebound Schema 65

PROTOCOL SYNOPSIS

Gilead Sciences, Inc.
333 Lakeside Dr.
Foster City, CA 94404

**Study Title:**	A Phase 3b Randomized, Open-label, Controlled Study of the Efficacy, Safety and Tolerability of 12 Weeks of Ledipasvir/Sofosbuvir (LDV/SOF) Treatment for HIV/HCV Co-infected Subjects who Switch to Elvitegravir/Cobicistat/Emtricitabine/Tenofovir Alafenamide (E/C/F/TAF) or Emtricitabine/Rilpivirine/Tenofovir Alafenamide (F/R/TAF) prior to LDV/SOF HCV Treatment, the HIV/HCV Co-STARs study (Co-infection treatment with Single Tablet Antiviral Regimens)
**IND Number:**	
**EudraCT Number:**	123098
**Clinical Trials.gov Identifier:**	2014-004545-27<br>TBD
**Study Centers Planned:**	Approximately 50 centers in North America
**Objectives:**	The primary objective of this study is as follows:<br>• To evaluate efficacy of LDV/SOF as measured by the proportion of subjects achieving HCV RNA below the lower limit of quantification (LLOQ) 12 weeks after the last dose of LDV/SOF (sustained virologic response [SVR12]).<br>The secondary objectives of this study are:<br>• To determine the proportion of subjects achieving HCV RNA below LLOQ 4 weeks after the last dose of LDV/SOF (SVR 4);<br>• To evaluate maintenance of HIV-1 RNA suppression after switching to E/C/F/TAF or F/R/TAF 24 weeks from the start of the F/TAF-based regimen;<br>• To evaluate the safety and tolerability of switching to E/C/F/TAF or F/R/TAF from the current antiretroviral (ARV) therapy in virologically-suppressed, HIV-1/HCV co-infected subjects;<br>• To evaluate the safety and tolerability of 12 weeks of treatment for HCV with LDV/SOF in virologically-suppressed, HIV-1/HCV co-infected subjects who switched to E/C/F/TAF or F/R/TAF.

**Study Design:**	Randomized, multicenter, open-label, 2-part study in adult male and female subjects with HIV who are co-infected with chronic genotype (GT) 1 HCV infection. Subjects will have one of the following:<br>1) no cirrhosis and no prior HCV treatment (treatment-naïve);<br>2) no cirrhosis and have received HCV treatment only with interferon (IFN) +/- ribavirin (RBV) or IFN + RBV + an HCV protease inhibitor (PI) (treatment-experienced); or<br>3) compensated cirrhosis and no prior HCV therapy (treatment-naïve).<br>Subjects will be on stable ARV therapy for 6 months prior to screening and with HIV RNA < 50 copies/mL.<br>**Part 1:** Approximately 240 subjects will be randomized 1:1 to switch from stable ARV therapy to one of the following treatments. Randomization will be stratified by race (black vs. non-black).<br>• **Treatment Group 1:** Switch from 2 nucleoside/nucleotide reverse transcriptase inhibitor (NRTI) plus a third ARV agent to E/C/F/TAF fixed dose combination (FDC) (n = 120)<br>• **Treatment Group 2:** Switch from 2 NRTI plus a third ARV agent to F/R/TAF FDC (n = 120)<br>All subjects will receive 8 weeks of treatment and will have an assessment of safety, tolerability and HIV-1 RNA values at the conclusion of 8 weeks. Plasma samples collected at Week 6 (laboratory visit only) will be included in assessing virologic suppression of HIV-1 RNA at Week 8. Subjects who tolerate the switch to E/C/F/TAF FDC or F/R/TAF FDC and maintain virologic suppression of HIV-1 RNA < 50 copies/mL will continue to Part 2. HIV treatment will be continued until their SVR12 assessment.<br>**Part 2:** After 8 weeks on the randomized E/C/F/TAF FDC or F/R/TAF FDC treatment, subjects will continue their HIV treatment and initiate 12 weeks of LDV/SOF HCV therapy.<br>• LDV/SOF FDC tablet once daily for 12 weeks<br>After LDV/SOF therapy, subjects will continue their HIV treatment and be followed for safety and efficacy for an additional 12 weeks to determine the HCV SVR12 endpoint and HIV safety and efficacy endpoints.

Number of Subjects Planned:

240 subjects (approximately 120 subjects randomized to each of E/C/F/TAF FDC or F/R/TAF FDC).

Target Population:

Chronic GT 1 HCV, HIV-coinfected adults who do not have cirrhosis and are HCV treatment-naïve or treatment-experienced with IFN +/- RBV +/- PI. Compensated cirrhotic subjects must be treatment-naïve. Subjects are on a stable HIV regimen with 2 NRTI + 3rd agent with suppressed HIV RNA.

Duration of Treatment:

Part 1: Subjects will be randomized to receive E/C/F/TAF or F/R/TAF for 8 weeks.

Part 2: Subjects will receive 12 weeks of LDV/SOF and continue their randomized F/TAF-based regimen for 12 weeks after completion of LDV/SOF (total approximately 24 weeks in Part 2).

Diagnosis and Main Eligibility Criteria:

HIV/HCV co-infected adult subjects who meet the following criteria will be given the option to participate in the study:

- Chronic GT 1, HCV infected, male and non-pregnant/non-lactating female subjects, without cirrhosis, treatment-naïve or treatment-experienced with IFN +/- RBV +/- HCV PI. Compensated cirrhotic subjects must be HCV treatment-naïve. No prior treatments with NS5A and NS5B or any HCV direct acting antivirals, except boceprevir, telaprevir and simeprevir, in combination with IFN and RBV.
- Currently on an ARV regimen (2 NRTI + a third agent) without change for 6 months prior to screening. Documented plasma HIV-1 RNA levels < 50 copies/mL (or undetectable HIV-1 RNA level according to the local assay being used if the limit of detection is ≥ 50 copies/mL) for ≥ 6 months preceding the Screening visit. After reaching HIV-1 RNA < 50 copies/mL, single values (“blips”) of HIV-1 RNA ≥ 50 copies/mL followed by resuppression is allowed. For subjects with 3 or more prior ARV regimens, a regimen history should be provided for approval by the Sponsor.
- Plasma HIV-1 RNA level < 50 copies/mL at the Screening visit.

- Have no documented resistance to any of the HIV study agents at any time in the past, including but not limited to the reverse transcriptase resistance mutations K65R, K70E, K101E/P, E138A/G/K/R/Q, V179L, Y181C/I/V, M184V/I, Y188L, H221Y, F227C, M230I/L, the combination of K103N+L100I, or 3 or more thymidine analog associated mutations (TAMs) that include M41L or L210W (TAMs are M41L, D67N, K70R, L210W, T215Y/F, K219Q/E/N/R). If a historical genotype prior to first ARV is not available or subject had 3 or more prior ARV regimens, subject will have proviral genotype analysis for archived resistance prior to Day 1.
- No history of HIV virologic failure.
- No evidence of Hepatitis B infection.
- Estimated glomerular filtration rate (eGFR) ≥ 30 mL/min as estimated by Cockcroft-Gault formula.

Study Procedures/ Frequency:

Screening assessments will be completed within 42 days of the Day 1 visit.

After consent is obtained, screening assessments will include physical examination, medical history, historical HIV resistance test collection (if available; HIV DNA archive genotype if no historical HIV genotype available), height, weight, vital signs, 12-lead electrocardiogram (ECG), screening procedure related adverse events (AEs), concomitant medications (CM), safety laboratory tests (including hematology, chemistry, and coagulation), HCV and HIV RNA, serology (HIV, HCV, HBV), HCV genotyping, assessment of the absence of cirrhosis, serum β-hCG (females of child bearing potential only), IL28B genotyping, and urinalysis.

On treatment assessment:

- **Part 1:** F/TAF-based ARV regimen switch:
  - Day 1 and Weeks 4, 6 (lab only), and 8
- **Part 2:** LDV/SOF therapy and follow-up
  - First dose of LDV/SOF at Week 8, then Weeks 12, 16, and 20 (4, 8, and 12 weeks after HCV start) and post LDV/SOF follow up at 4 and 12 weeks posttreatment (SVR 4 and 12).

On treatment assessments will include AEs, CMs, study drug dosing adherence (including pill count), physical examination, weight, vital signs, safety laboratory tests, HCV and HIV RNA, urine pregnancy tests (females of child bearing potential only).

	Fasting lipid profile measurements and fasting glucose will be performed at Day 1, Weeks 8, 20, and at Post-HCV Treatment Week 12. Lipids will include total cholesterol (TC), high density and low density lipoproteins (HDL, LDL), non-HDL cholesterol, triglycerides (TG) and cholesterol ratios.<br><br>Post treatment assessments will include AEs and CMs collected through 30 days after the last dose of HIV study drug, HCV and HIV RNA, safety laboratory tests (including hematology and chemistry), and urine pregnancy tests (females of childbearing potential only).<br><br>Plasma samples for viral HIV RNA will be collected at Day 1 and every visit thereafter (except HCV RNA at Week 4). Stored samples for HCV RNA sequencing/phenotyping and HIV RNA genotyping/phenotyping will be collected at Day 1 and every visit thereafter. Blood will be collected at Screening for analysis of HIV proviral DNA genotype, as needed.<br><br>Calculated creatinine clearance, hematology, serum chemistry, and urinalysis tests will be performed at all visits.<br><br>Health Related Quality of Life (HRQoL) Surveys will be conducted at Day 1, Week 8, 20, and Post-HCV Treatment Week 12 visits.<br><br>PPD [redacted].<br><br>Pharmacokinetic (PK) blood samples will be collected at specified time points pre- and/or postdose at post-Day 1 visits.
**Test Product, Dose, and Mode of Administration:**	Elvitegravir 150 mg/cobicistat 150 mg/emtricitabine 200 mg/ tenofovir alafenamide 10 mg (E/C/F/TAF) FDC administered once daily with food.<br><br>Emtricitabine 200 mg/rilpivirine 25 mg/tenofovir alafenamide 25 mg (F/R/TAF) FDC administered once daily with food.<br><br>Ledipasvir 90 mg/sofosbuvir 400 mg (LDV/SOF) FDC administered once daily with or without food.
**Reference Therapy, Dose, and Mode of Administration:**	None.

Criteria for Evaluation:

Safety:

Adverse events will be collected at all visits. Clinical laboratory tests will be performed at all visits.

The safety and tolerability of the treatment regimens will be assessed with adverse event and clinical laboratory tests (including selected evaluations of renal function and PPD

Efficacy:

Efficacy will be evaluated using scheduled assessments of HCV RNA performed using COBAS® Ampliprep / COBAS® TaqMan® HCV Quantitative Test, v2.0 and COBAS® TaqMan® HIV RNA Test, v2.0.

Primary endpoint

- HCV RNA < lower limit of quantification (LLOQ) 12 weeks after completion of LDV/SOF treatment (SVR12)

Secondary endpoints

Efficacy:

- HCV RNA < LLOQ 4 weeks after completion of LDV/SOF treatment (SVR4)
- HIV-1 RNA ≥ 50 copies/mL (virologic failure) 24 weeks after start of the F/TAF-based regimen using modified FDA snapshot algorithm

Safety:

- Grades 1 through 4 adverse events after switching to E/C/F/TAF or F/R/TAF throughout the study and during coadministration with LDV/SOF treatment.

Pharmacokinetics:

Samples collected for potential population PK assessment.

Health Related Questionnaires:

Health related questionnaires will be administered, including the Adherence Visual Analogue Scale (VAS), HIV Treatment Satisfaction (HIVTSQ), Medical Outcome Study Short Form-36 (SF-36), Functional Assessment of Chronic Illness Therapy-Fatigue scale (FACIT-F), chronic liver disease questionnaire (CLDQ-HCV) and work productivity and activity impairment questionnaire (WPAI).

Statistical Methods:

This is a two part, randomized (for HIV therapy) multicenter study to evaluate the efficacy and safety of switching to a F/TAF-based regimen in HCV co-infected subjects on a current suppressive ARV regimen.

The primary efficacy endpoint is SVR12 (HCV RNA < LLOQ 12 weeks post HCV treatment) in the HCV Full Analysis Set. The primary analysis will use a two-sided exact one-sample binomial test at the 0.05 significance level and construct an associated two-sided exact 95% confidence interval (CI).

Secondary efficacy analyses will evaluate the proportion of subjects with HCV RNA < LLOQ at 4 weeks post coadministration of LDV/SOF treatment. Assessment of HIV-1 RNA ≥ 50 copies/mL (virologic failure) will be done at 24 weeks after start of the F/TAF-based regimen using modified FDA snapshot algorithm. Safety analyses will evaluate adverse events (clinical and laboratory) during switch to F/TAF-based regimen and during coadministration with LDV/SOF treatment. Additional safety analyses will evaluate the proportion of subjects who discontinued from study treatment (HIV or HCV) for an AE.

Additional endpoints to be studied will include the effect of therapy on health-related quality of life.

Assuming that at least 94% of subjects achieve HCV SVR12, the 95% CI around the SVR12 rate will be ± 3.2% using exact methods.

The assumed SVR12 rate will have at least 85% power to be greater than 6 percentage points from a performance goal of 88% by using a two-sided exact one-sample binomial test at significance level of 0.05.

This study will be conducted in accordance with the guidelines of Good Clinical Practice (GCP) including archiving of essential documents.

GLOSSARY OF ABBREVIATIONS AND DEFINITION OF TERMS

° C	degrees Celsius
° F	degrees Fahrenheit
3TC	lamivudine
AASLD/IDSA	American Association for The Study of Liver Diseases/Infectious Diseases Society of America
ABC	abacavir
AE	adverse event
AIDS	acquired immunodeficiency syndrome
ALT (SGPT)	alanine aminotransferase
APTT	activated partial thromboplastin time
ART	antiretroviral therapy
ARV	antiretroviral
AST (SGOT)	aspartate aminotransferase
APRI	AST: platelet ratio index
ATR	Atripla®
ATV	atazanavir
AUC	area under the concentration vs. time curve
AUC_{inf}	area under the concentration verses time curve extrapolated to infinite time, calculated as AUC0-last + (Clast/λz)
AUC_{last}	area under the concentration curve verses time curve from time zero to the last quantifiable concentration
AZT	zidovudine
BMD	bone mineral density
C_{last}	last observed quantifiable plasma concentration of drug
C_{max}	maximum observed plasma concentration of drug
C_{tau}	observed drug concentration at the end of the dosing interval
C_x	minimum observed quantifiable plasma concentration x hours after the last dose of drug
CBC	complete blood count
CFR	Code of Federal Regulations
CI	confidence interval
CK	creatinine kinase
CL_{cr}	creatinine clearance
CLDQ-HCV	chronic liver disease questionnaire
CM	concomitant medications
CMH	Cochran-Mantel-Haenszel
COBI	cobicistat, C
CRO	Contract Research Organization
CSR	clinical study report
DAA	direct acting antiviral(s)
DDI	drug-drug interaction

DF	disoproxil fumarate
dL	deciliter
DNA	deoxyribonucleic acid
DP	diphosphate
DRV	darunavir
DSPH	Drug Safety and Public Health
DTG	dolutegravir, Tivicay®
ECG	electrocardiogram
eCRF	electronic case report forms(s)
EDC	electronic data capture
EFV	efavirenz, Sustiva®
eGFR	estimated glomerular filtration rate
eGFRcg	estimated glomerular filtration rate, Cockcroft-Gault
ESA	erythropoiesis-stimulating agents
eSAE	electronic serious adverse event
ESDD	Early Study Drug Discontinuation
ETR	etravirine
EVG	elvitegravir, E
EU	European Union
E/C/F/TAF	elvitegravir/cobicistat/emtricitabine/tenofovir alafenamide, Genvoya®
FACIT-F	functional assessment of chronic illness therapy-fatigue scale
FAS	full analysis set
FDA	(U.S.) Food and Drug Administration
FDC	fixed dose combination
FPV	fosamprenavir
FTC	emtricitabine, F
F/R/TAF	emtricitabine/rilpivirine/tenofovir alafenamide, Odefsey®
FTC/RPV/TDF	emtricitabine/rilpivirine/tenofovir disoproxil fumarate, Complera®
FTC/TAF	emtricitabine/tenofovir alafenamide, Descovy®
FTC/TDF	emtricitabine/tenofovir disoproxil fumarate, Truvada®
g	gram(s)
GCP	Good Clinical Practice (Guidelines)
GCSF	granulocyte colony stimulating factor
GLSM/GMR	geometric least squares mean ratio
GGT	gamma glutamyl transferase
GSI	Gilead Sciences, Inc.
GT	genotype
h	hour(s)
HAART	highly active antiretroviral therapy
HBV	hepatitis B virus
HBsAg	hepatitis B surface antigen

HCC	Hepatocellular carcinoma
HCV	hepatitis C virus
HDL	high density lipoprotein
HDPE	high-density polyethylene
HIV-1	human immunodeficiency virus type 1
HIV-2	human immunodeficiency virus type 2
HIVTSQc	HIV Treatment Satisfaction Questionnaire – Change version
HIVTSQs	HIV Treatment Satisfaction Questionnaire – Status version
HLGT	high-level group term
HLT	high-level term
HRQoL	Health Related Quality of Life
IB	investigator’s brochure
ICH	International Conference on Harmonisation
IFN	interferon
IEC	Independent Ethics Committee
IMP	investigational medicinal product
INR	International Normalized Ratio
INSTI	integrase strand transfer inhibitor
IRB	Institutional Review Board
IUD	intrauterine device
IWRS	Interactive Web Response System
kg	kilogram
kPa	kilopascal
KS	Kaposi's sarcoma
LDL	low density lipoprotein
LDV/SOF	ledipasvir/sofosbuvir, Harvoni®
LLN	lower limit of the normal range
LLOQ	lower limit of quantification
LLT	lower–level term
LPV	lopinavir
MedDRA	Medical Dictionary for Regulatory Activities
mg	milligram
min	minute
mL	milliliter
m^2	square meter
mm^3	cubic millimeter(s)
µg	microgram
µm	micrometer
ng	nanogram
NNRTI	non-nucleoside reverse transcriptase inhibitor
NRTI	nucleoside reverse transcriptase inhibitor

NVP	nevirapine, Viramune®
PEG-IFN	pegylated interferon
P-gp	P-glycoprotein
PEP	post-exposure prophylaxis
PI	protease inhibitor
PK	pharmacokinetic
PT	preferred term
PT	prothrombin time
QA	Quality Assurance
QD	once daily
RAL	raltegravir, Isentress®
RBV	ribavirin
RNA	ribonucleic acid
RT	reverse transcriptase
RTV	ritonavir
RPV	rilpivirine, R, Edurant®
SAE	serious adverse event
SADR	serious adverse drug reactions
SF-36	medical outcome study short form-36
sGPVI	soluble glycoprotein VI
SOC	system organ class
SOF	sofosbuvir, Sovaldi®
SOP	standard operation procedure
STB	Stribild®
STR	Single Tablet Regimen
SUSAR	Suspected Unexpected Serious Adverse Reaction
SVR	sustained virologic response
T_{last}	time (observed time point) of Clast
T_{max}	time (observed time point) of Cmax
TAF	tenofovir alafenamide
TAF fumarate	tenofovir alafenamide fumarate
TAM	thymidine analog associated mutations
TDF	tenofovir disoproxil fumarate, Viread®
TFV	tenofovir
TPO	thrombopoietin
TVD	Truvada®
ULN	upper limit of the normal range
US	United States
VAS	visual analogue scale
VR	virologic rebound
wt	Weight
WPAI	work productivity and activity impairment questionnaire

1. INTRODUCTION

1.1. Background

HIV Infection

Human Immunodeficiency Virus type 1 (HIV-1) infection is a life-threatening and serious disease that is of major public health interest around the world. There are 35 million people worldwide and approximately 1.1 million people in the US living with HIV-1 {Campsmith et al 2010}, {World Health Organization (WHO) 2011}. Within Western and Central Europe, it is estimated that there are over 900,000 individuals living with HIV and 131,000 new infections in 2012 {The Joint United Nations Programme on HIV/AIDS (UNAIDS) 2012}.

If left untreated or suboptimally treated, HIV infection is characterized by deterioration in immune function, the subsequent occurrence of opportunistic infections and malignancies, ultimately resulting in death. Therapeutic strategies for the treatment of HIV-1 disease have been significantly advanced by the availability of highly active antiretroviral therapy (HAART); the introduction of HAART was associated with a dramatic decrease in acquired immune deficiency syndrome (AIDS)-related morbidity and mortality {Palella et al 1998}, {Mocroft et al 1998}, {Sterne et al 2005}.

The primary goals of ARV therapy for HIV-1 infection are to reduce HIV-associated morbidity and prolong the duration and quality of life, restore and preserve immunologic function, maximally and durably suppress plasma HIV viral load, and prevent HIV transmission. The DHHS guidelines list emtricitabine/tenofovir DF (FTC/TDF) as a preferred nucleos(t)ide reverse transcriptase inhibitor NRTI/NtRTI backbone in combination with either cobicistat-boosted elvitegravir [EVG/COBI] administered as EVG/COBI/FTC/TDF, raltegravir, dolutegravir or darunavir/ritonavir as an initial ARV regimen {Department for Health and Human Services (DHHS) 2015}. Although HAART has dramatically improved the prognosis of patients infected with HIV-1, eradication of the virus is not possible with currently available therapies. Long-term viral suppression and prevention of drug resistance are goals of successful therapy. In regimens of comparable efficacy, the total pill burden, dosing frequency, and concerns about safety and side effects are generally the most significant obstacles to achieving high adherence {Stone VE 2002}, {Chesney 2000}, {Department for Health and Human Services (DHHS) 2015}.

Tenofovir disoproxil fumarate (TDF) is a preferred NRTI among recommended regimens for treatment-naïve HIV-positive patients, but is associated with nephrotoxicity and reduced bone mineral density (BMD) {Panel on Antiretroviral Guidelines for Adults and Adolescents 2012}. Lifelong antiretroviral treatment and the increasing comorbidities being recognized and treated in HIV-positive patients creates an urgent need to improve the safety profile of regimens that most effectively suppress HIV replication. Tenofovir alafenamide (TAF) is novel oral prodrug of tenofovir (TFV), a nucleotide analog that inhibits HIV-1 reverse transcription. Gilead has coformulated TAF with the integrase strand transfer inhibitor elvitegravir (EVG), cobicistat (COBI), and emtricitabine (FTC) into a fixed-dose combination (FDC). Compared to TDF, the

use of TAF in the E/C/F/TAF FDC provides enhanced lymphatic delivery of tenofovir, resulting in higher intracellular levels of the active phosphorylated moiety tenofovir-diphosphate, and lower systemic circulating levels of tenofovir. These features translate into an improved tolerability and safety profile, especially with respect to renal and bone safety {Sax et al 2015}.

HCV and HIV/HCV Co-Infection

Hepatitis C virus (HCV) infection is a global health challenge with an estimated 150 million individuals infected worldwide {Gay et al 2011}. Currently there are 6 major HCV genotypes recognized {Nakano et al 2012}. Genotypes 1, 2, and 3 are common throughout North America and Europe and when combined account for approximately 75% of the chronic HCV infections globally. Genotype 4, which is common in the Middle East and Africa, accounts for approximately 20% of the global HCV population. Genotypes 5 and 6 are the least prevalent and are generally found in South Africa and South East Asia, respectively {Lavanchy 2011}.

In the United States (US), approximately 2.7 million people have chronic HCV infection {Denniston et al 2014} and HCV infection causes over 15,000 deaths each year {Ly et al 2012}, although under-reporting of HCV infection on death certificates may contribute to as much as a 5-fold underestimation of the actual number of deaths {Mahajan et al 2014}. Of the HCV related deaths that are reported, almost three quarters occurred in the baby-boomer generation with a median age of death of 57 years, which is approximately 20 years less than the average lifespan {Smith et al 2012}. Successful treatment of chronic HCV infection reduces the need for liver transplant, the incidence of hepatocellular carcinoma (HCC) and overall mortality {Backus et al 2011}. Thus, the public health benefit of safe and effective HCV treatment regimens is high.

HIV-infected patients are frequently co-infected with HCV because of the shared routes of infection. HIV/HCV co-infection is estimated to occur in one quarter of HIV-infected persons in Europe, Australia, and the United States, {Thomas 2008}, and in a study from the Veterans Affairs Medical Center in Atlanta, Georgia, HCV infection occurred as many as 33% of the patients with HIV infection {Staples et al 1999}. The use of antiretroviral therapy (ART) has markedly reduced opportunistic infections and HIV-infected persons are living longer. However, liver disease has emerged as the main cause of morbidity and mortality in the HIV positive patient {Salmon-Ceron et al 2005}, and the development of end stage liver disease seems to be unrelated to the CD4 count and the immune virological status of the patient {Salmon-Ceron et al 2005}. In two meta-analyses published 8 years apart, co-infected patients were 5 to 6 times more likely to develop decompensated liver failure and 1.5 to 2 times more likely to develop histological cirrhosis {Graham et al 2001}, {Deng et al 2009}. In the latter analysis, the risk of death in the co-infected patients was nearly 4 times greater compared with that in mono-infected individuals {Myers et al 2008}. As might be expected, hospitalization rates due to liver disease in the co-infected patient have increased {Laguno et al 2004}. Furthermore, the previous gold standard therapy for the treatment of HCV infection, pegylated nterferon alfa (PEG-IFN) plus ribavirin (RBV), appears to be less effective in patients with co-infection than in patients with HCV mono-infection {Torriani et al 2004}, {Carrat et al 2004}.

The development of Sovaldi® (sofosbuvir, SOF), a nucleotide analog HCV NS5B polymerase inhibitor, represents a major advance in the treatment of HCV as SOF-based regimens are shorter in duration, better tolerated and result in higher sustained virologic response (SVR) rates than prior therapies. SOF in combination with ribavirin (RBV) ± pegylated interferon (PEG-IFN) for varying durations was approved in the US and Canada in December 2013 for the treatment of genotype 1, 2, 3 and 4 HCV infection and was granted marketing authorization by the European Commission in January 2014 for the treatment of genotypes 1-6 HCV infection {Gilead Sciences Inc 2013}, {Gilead Sciences Canada Inc. 2013}, {Gilead Sciences Limited 2014}. LDV/SOF is the first IFN-free regimen with high SVR rates following 8-24 weeks of treatment across treatment-naïve and treatment-experienced patients, including patients who failed to achieve SVR with PEG + RBV ± NS3/4A protease inhibitors, irrespective of cirrhosis {Gilead Sciences Inc. 2014}.

1.2. Tenofovir Alafenamide (TAF, GS-7340)

1.2.1. General Information

Tenofovir alafenamide (GS-7340, TAF, or L-Alanine, *N*-[(*S*)-[[(1*R*)-2-(6-amino-9*H*-purin-9-yl)-1-methylethoxy]methyl]/phenoxyphosphinyl]-, 1-methylethyl ester) is a second generation oral prodrug of TFV, a nucleotide analog that inhibits HIV-1 reverse transcription. TFV is metabolized intracellularly to the active metabolite, TFV-DP, a competitive inhibitor of HIV-1 reverse transcriptase (RT) that terminates the elongation of the viral DNA chain. In the development of TAF, three forms of the drug substance have been used in various studies: GS-7340, synonym for GS-7340 as the free base; GS-7340-02, synonym for TAF monofumarate (1:1) molar ratio of TAF to fumaric acid; and GS-7340-03 as the hemifumarate (2:1) molar ratio of TAF to fumaric acid. GS-7340-03, also known as TAF fumarate, is considered comparable based on physical/chemical properties to GS-7340-02 that has been used in previous studies. GS-7340-02 and GS-7340-03 exist as the free base, TAF (GS-7340), in blood and biological fluids.

For further information on the preclinical pharmacology and toxicology of TAF, refer to the current Investigator's Brochure (IB).

1.2.2. Clinical Trials of Elvitegravir/Cobicistat/Emtricitabine/Tenofovir Alafenamide (E/C/F/TAF, Genvoya®)

Elvitegravir, an HIV-1 integrase strand transfer inhibitor (INSTI), cobicistat, a CYP3A inhibitor, and emtricitabine and tenofovir alafenamide (TAF), both HIV-1 nucleoside analog reverse transcriptase inhibitors (NRTIs) are co-formulated into a single-tablet regimen, E/C/F/TAF, or Genvoya®. E/C/F/TAF is indicated as a complete regimen for the treatment of HIV-1 infection in adults and pediatric patients 12 years of age and older who have no antiretroviral treatment history or to replace the current antiretroviral regimen in those who are virologically suppressed (HIV-1 RNA less than 50 copies per mL) on a stable antiretroviral regimen for at least 6 months with no history of treatment failure and no known substitutions associated with resistance to the individual components of the regimen. E/C/F/TAF is not recommended in patients with estimated creatinine clearance below 30 mL per minute. The efficacy and safety of E/C/F/TAF continue to be evaluated in a broad clinical development program. Available data from key studies are described in the most current version of the E/C/F/TAF IB. The details of the key clinical studies are provided below.

- Study GS-US-292-0102 is a Phase 2, randomized, double-blind, double-dummy, multicenter, active-controlled study which evaluated the safety and efficacy of the novel tenofovir prodrug, tenofovir alafenamide (TAF), as part of a single-tablet regimen for the initial treatment of HIV-1 infection. Antiretroviral naive adults with HIV-1 RNA ≥ 5000 copies per milliliter and a CD4 count ≥ 50 cells per microliter were randomized 2:1 to receive an STR of elvitegravir/cobicistat/emtricitabine/tenofovir alafenamide (E/C/F/TAF) or elvitegravir/cobicistat/emtricitabine/tenofovir disoproxil fumarate (E/C/F/TDF), plus placebo for 48 weeks. Patients on both E/C/F/TAF (n = 112) and E/C/F/TDF (n = 58) had high rates of virologic suppression (< 50 HIV copies per milliliter) at Week 24 (86.6%; 89.7%) and at Week 48 (88.4%; 87.9%), and had similar improvements in CD4 at Week 48 (177; 204), respectively. Both treatments were well tolerated, and most adverse events were self-limiting and of mild to moderate severity. Compared with patients on E/C/F/TDF, patients on E/C/F/TAF had smaller reductions in estimated creatinine clearance (-5.5 vs. -10.1 mL/min, p = 0.041), significantly less renal tubular proteinuria, and smaller changes in bone mineral density for hip (-0.62% vs. -2.39%, p < 0.001) and spine (-1.00% vs. -3.37%, p < 0.001). Patients on E/C/F/TAF had higher increases in total cholesterol, low density lipoprotein, and high-density lipoprotein, but the total cholesterol/high-density lipoprotein ratio was unchanged for both. Treatment-naive patients given the STR that contained either TAF or TDF achieved a high rate of virologic success. Compared with those receiving TDF, patients on E/C/F/TAF experienced significantly smaller changes in estimated creatinine clearance, renal tubular proteinuria, and bone mineral density. The efficacy data from this Phase 2 study supported the Phase 3 studies and provided initial evidence of durable efficacy; 87.5% of subjects who received E/C/F/TAF achieved and maintained HIV-1 RNA < 50 copies/mL through Week 96 (M = F).
- GS-US-292-0104 and GS-US-292-0111 are two Phase 3, randomized double-blind, double-dummy, multicenter, active-controlled studies which evaluated the efficacy and safety of two single-tablet regimens, E/C/F/TAF vs E/C/F/TDF. Key inclusion criteria were estimated creatinine clearance of at least 50 mL per min and genotypic sensitivity to study drugs. The main outcomes were the proportion of patients with plasma HIV-1 RNA less than 50 copies per mL at Week 48 as defined by the US Food and Drug Administration (FDA) snapshot algorithm (pre-specified noninferiority margin of 12%) and pre-specified renal and bone endpoints at 48 weeks. Secondary endpoints were at Weeks 96 and 144. 1733 patients (866 E/C/F/TAF and 867 E/C/F/TDF) were enrolled (or completed the study). E/C/F/TAF was non-inferior to E/C/F/TDF, with 92% of (800/866) patients in the TAF group and 90% of (784/867) patients in the TDF group having plasma HIV-1 RNA less than 50 copies per mL (adjusted difference 2.0%, 95% CI –0.7 to 4.7). Patients given E/C/F/TAF had significantly smaller mean serum creatinine increases than those given E/C/F/TDF (0.08 vs 0.12 mg/dL; p < 0.001), significantly less proteinuria (median % change –3 vs 20; p < 0.001), and a significantly smaller decrease in bone mineral density at spine (mean % change –1.30 vs –2.86; p < 0.001) and hip (–0.66 vs –2.95; p < 0.001) at 48 weeks. At 96 weeks, 86.6% in the E/C/F/TAF arm and 85.2% in the E/C/F/TDF arm had HIV-1 RNA < 50 c/mL (difference 1.5%; 95%CI [-1.8%, 4.8%]). With E/C/F/TAF, decreases in lumbar spine and hip bone mineral density and proteinuria were significantly less and creatinine increases were significantly smaller. These longer-term data support the use of E/C/F/TAF as a safe, well-tolerated and durable regimen for initial HIV-1 treatment.

- Study GS-US-292-0109 is a Phase 3, randomized, active-controlled, multicenter, open-label, non-inferiority trial evaluating efficacy, safety, and tolerability of switching to E/C/F/TAF versus continuing a TDF-containing regimen (ie, Atripla, Stribild, or COBI- or RTV-boosted ATV with FTC/TDF). Key inclusion criteria included virological suppression (HIV-1 RNA < 50 copies per mL) for at least 6 months on Atripla, Stribild, or COBI- or RTV-boosted ATV with FTC/TDF and an estimated creatinine clearance of at least 50 mL per min. The primary endpoint was the proportion of patients who received at least one dose of study drug who had undetectable viral load (HIV-1 RNA < 50 copies per mL) at Week 48. 959 patients were randomly assigned to E/C/F/TAF and 477 continued Atripla, Stribild, or COBI- or RTV-boosted ATV with FTC/TDF. Viral suppression at Week 48 was noted in 97% (932/959) of patients switched to E/C/F/TAF and 93% (444/477) of patients who continued a TDF-containing regimen (adjusted difference 4.1%, 95% CI 1.6–6.7), with virological failure noted in ten and six patients, respectively. The number of adverse events was similar between the two groups, but study drug-related adverse events were more common in the tenofovir alafenamide group (204 patients [21%] vs 76 [16%]). Hip and spine bone mineral density and glomerular filtration were each significantly improved in patients in the E/C/F/TAF group compared with those in the TDF-containing regimen group.

- GS-US-292-0112 is a Phase 3, single-arm, open-label study which evaluated the efficacy and safety of E/C/F/TAF in virologically suppressed, HIV-infected subjects with estimated creatinine clearance of 30-69 mL/min over 144 weeks. The primary endpoint was the change from baseline in glomerular filtration rate estimated using various formula. A total of 242 virologically suppressed patients enrolled and switched treatment to E/C/F/TAF. Subject baseline characteristics were mean age 58 years, 18% black, 39% hypertension, 14% diabetes. Through Week 48, no significant change in estimated CrCl was observed. Two patients (0.8%) discontinued study drug for decreased creatinine clearance, neither had evidence of renal tubulopathy and both had uncontrolled hypertension. Subjects had significant improvements in proteinuria, albuminuria, and tubular proteinuria ($p < 0.001$ for all). Hip and spine bone mineral density significantly increased from baseline to Week 48 (mean percent change +1.47 and +2.29, respectively, $p < 0.05$). 92% (222 patients) maintained HIV-1 RNA < 50 copies/mL at Week 48. The findings of this study support the use of E/C/F/TAF in patients with renal impairment down to an $eGFR_{CG}$ of 30 mL/min.

1.2.3. Clinical Trials of Emtricitabine/Rilpivirine/Tenofovir Alafenamide (F/R/TAF, Odefsey®) Fixed Dose Combination

The details of the key clinical studies are provided below. Please refer to the IB for additional information on the F/R/TAF FDC.

- GS-US-366-1159 was a randomized, open-label, single-dose, 3-way, 6-sequence, crossover study to determine the bioequivalence of FTC and TAF, administered as E/C/F/TAF FDC tablet or as F/R/TAF (Odefsey®) FDC tablet, and the bioequivalence of RPV administered as RPV single tablet or as F/R/TAF FDC tablet. The FTC and TAF components of the F/R/TAF (200/25/25 mg) FDC were found to be bioequivalent to the E/C/F/TAF (150/150/200/10 mg) FDC. The RPV component of the F/R/TAF (200/25/25 mg) FDC was found to be

bioequivalent to RPV 25 mg tablet (Edurant®). FTC, RPV, and TAF administered as F/R/TAF (200/25/25 mg) FDC, RPV (25 mg), and E/C/F/TAF (150/150/200/10 mg) FDC were generally safe and well-tolerated in healthy volunteers.

- GS-US-366-1651 was a randomized, open-label, single-dose, 2-period, crossover, food-effect study. Two cohorts of subjects were enrolled to assess the effect of moderate-fat food and the effect of high-calorie, high-fat food, on the PK of a single-dose F/R/TAF FDC tablet. Overall TAF exposure (AUC_{last}) increased by approximately 45% and 54%, FTC exposure (AUC_{inf}) decreased by approximately 9% and 12%, and RPV exposure (AUC_{inf}) increased by approximately 13% and 75% (moderate-fat and high-calorie, high-fat conditions, respectively) when the RPV/FTC/TAF FDC tablet (200/25/25 mg) was administered under fed compared with fasted conditions.

1.3. Ledipasvir/Sofosbuvir (LDV/SOF, Harvoni®) Fixed Dose Combination

1.3.1. General Information

Ledipasvir/sofosbuvir fixed-dose combination (LDV/SOF FDC, or Harvoni®) combines two HCV specific DAA agents into a single tablet for the treatment of chronic HCV infection. Sofosbuvir is a nucleotide analog that is a potent and selective inhibitor of NS5B-directed HCV replication, irrespective of HCV genotype. Ledipasvir is a novel HCV NS5A inhibitor that has demonstrated potent anti-HCV activity against genotypes 1 and 4 HCV infection.

Please refer to the Investigator’s Brochure (IB) for additional information on the LDV/SOF FDC, and the individual components, including:

- In Vitro Anti-Hepatitis C Virus Activity
- Nonclinical Pharmacokinetics and In Vitro Metabolism
- Nonclinical Pharmacology and Toxicology

1.3.2. Clinical Trials of Ledipasvir/Sofosbuvir (LDV/SOF, Harvoni®) Relevant to the Proposed Study

- Study GS-US-337-0102 (ION-1) was a Phase 3, multicenter, randomized, open-label study designed to investigate the efficacy and safety of LDV/SOF FDC + RBV for 12 and 24 weeks. Subjects were HCV treatment-naive with chronic genotype 1 HCV infection. ION-1 was based on data from Phase 2 studies, which demonstrated high SVR rates in subjects with HCV genotype 1 infection. Subjects were randomized 1:1:1:1 to receive LDV/SOF once daily for 12 weeks, LDV/SOF + RBV for 12 weeks, LDV/SOF for 24 weeks, or LDV/SOF + RBV for 24 weeks. The primary end point was SVR12.

  Baseline characteristics of the 865 randomized and treated subjects included 16% with cirrhosis, 12% black, and 67% with HCV genotype 1a infection. The rates of SVR12 were 99% (95% confidence interval [CI], 96 to 100) in the group that received 12 weeks of

LDV/SOF; 97% (95% CI, 94 to 99) in the group that received 12 weeks of LDV/SOF plus ribavirin; 98% (95% CI, 95 to 99) in the group that received 24 weeks of LDV/SOF; and 99% (95% CI, 97 to 100) in the group that received 24 weeks of LDV/SOF plus ribavirin. No subject in either 12-week group discontinued LDV/SOF owing to an adverse event. The most common adverse events were fatigue, headache, insomnia, and nausea. The study demonstrated that once-daily LDV/SOF+/- RBV for 12 or 24 weeks was highly effective in previously untreated patients with HCV genotype 1 infection.

- Study GS-US-337-0109 (ION-2) was a Phase 3, randomized, open-label study of HCV genotype 1 subjects with prior virologic failure of peg-interferon and ribavirin +/- an HCV protease inhibitor. Subjects were randomized to i) LDV/SOF for 12 weeks, ii) LDV/SOF + RBV for 12 weeks, iii) LDV/SOF for 24 weeks, or iv) LDV/SOF + RBV for 24 weeks. The primary end point was SVR12. Baseline characteristics of the 440 randomized and treated subject included: 20% with cirrhosis and 79% with HCV genotype 1a infection. SVR12 rates were: 94% (95% confidence interval [CI], 87 to 97) for 12 weeks of LDV/SOF; 96% (95% CI, 91 to 99) for 12 weeks of LDV/SOF + RBV; 99% (95% CI, 95 to 100) for 24 weeks of LDV/SOF; and 99% (95% CI, 95 to 100) for 24 weeks of LDV/SOF + RBV. No patient discontinued treatment owing to an adverse event. The most common adverse events were fatigue, headache, and nausea. The study demonstrated that treatment with daily LDV/SOF resulted in high rates of SVR12 in HCV-infected subjects who had not had a sustained virologic response to prior interferon based treatment.

- Study GS-US-337-0108 (ION-3) was a Phase 3, open-label study, which randomly assigned 647 previously untreated subjects with HCV genotype 1 infection without cirrhosis to receive LDV/SOF +/- RBV for 8 weeks or LDV/SOF for 12 weeks. The primary end point was SVR12. The rate of sustained virologic response was 94% (95% confidence interval [CI], 90 to 97) with 8 weeks of LDV/SOF, 93% (95% CI, 89 to 96) with 8 weeks of LDV/SOF + RBV, and 95% (95% CI, 92 to 98) with 12 weeks of LDV/SOF. As compared with the SVR12 rate in the group that received 8 weeks of LDV/SOF, the rate in the 12-week group was 1 percentage point higher (97.5% CI, −4 to 6) and the rate in the group that received 8 weeks of LDV/SOF + RBV was 1 percentage point lower (95% CI, −6 to 4) demonstrating noninferiority of the 8-week LDV/SOF regimen (noninferiority margin of 12%). Adverse events were more common in the group that received ribavirin than in the other two groups. No patient who received 8 weeks of only LDV/SOF discontinued treatment owing to adverse events. Overall, previously untreated subjects treated with 8 weeks of LDV/SOF achieved high rates of SVR. No additional benefit was associated with the inclusion of ribavirin in the regimen or with extension of the duration of treatment to 12 weeks.

- GS-US-337-0115 (ION-4) was a multicenter, single-arm, open-label study that enrolled HIV/HCV (genotype 1 or 4) coinfected subjects to receive 12 weeks of LDV/SOF. Subjects were stable on antiretroviral regimens of F/TDF with EFV, RPV or RAL. The primary end point was SVR12. Of the 335 patients enrolled, 34% were black, 55% had been previously treated for HCV, and 20% had cirrhosis. Overall, 322 patients (96%) had SVR12 (95% confidence interval [CI], 93 to 98), including rates of 96% (95% CI, 93 to 98) in patients with HCV genotype 1a, 96% (95% CI, 89 to 99) in those with HCV genotype 1b, and 100% (95%

CI, 63 to 100) in those with HCV genotype 4. Rates of sustained virologic response were similar regardless of previous treatment or the presence of cirrhosis. Of the 13 subjects who did not have a sustained virologic response, 10 had a relapse after the end of treatment. No subject had confirmed HIV-1 virologic rebound. The most common adverse events were headache (25%), fatigue (21%), and diarrhea (11%). No subejct discontinued treatment because of adverse events. The study concluded that LDV/SOF for 12 weeks provided high rates of sustained virologic response in patients coinfected with HIV-1 and HCV genotype 1 or 4.

Combined Analyses of LDV/SOF Data {Bhagani et al 2015}

A combined analysis compared the safety and efficacy of the single tablet regimen of LDV/SOF in HCV genotype (GT) 1 patients co-infected with HIV-1 in the Phase 3 ION-4 study with HCV mono-infected GT1 patients in the Phase III ION 1-3 studies.

In the ION-4 study, 327 GT1 HCV/HIV co-infected patients (126 treatment-naïve, non-cirrhotic, 134 treatment-experienced, non-cirrhotic, 20 treatment-naïve, cirrhotic, 47 treatment-experienced, cirrhotic) received LDV/SOF for 12 weeks. In the ION 1-3 studies, 538 GT1 HCV mono-infected patients (395 treatment-naïve, non-cirrhotic, 87 treatment-experienced, non-cirrhotic, 34 treatment-naïve, cirrhotic, 22 treatment-experienced, cirrhotic) received LDV/SOF for 12 weeks. The pooled analysis assessed safety and sustained virologic response at week 12 (SVR12).

Overall, 865 patients were treated for 12 weeks. In ION 1-3, the majority of patients were male (329, 61%), non-black (449, 83%), and treatment-naïve (430, 80%) while 56 (10%) patients had cirrhosis. In ION-4, the majority of patients were male (276, 82%), non-black (220, 66%), and treatment-experienced (185, 55%). Sixty-seven (67, 20%) patients had cirrhosis. SVR12 and relapse data are reported in the table. Treatment was well tolerated in both mono-infected and co-infected patients. Most common adverse events (> 10% reported in any arm) were fatigue, headache, diarrhea, and nausea. Only two patients discontinued treatment due to an adverse event. The investigators concluded that the once daily, single tablet regimen of LDV/SOF for 12 weeks provided high rates of SVR regardless of presence of HIV infection and was a safe, well-tolerated option for patients with both HCV mono-infection and HIV/HCV co-infection.

Table 1-1. Combined Analysis from Phase III ION Studies

Study	ION 1	ION 2	ION 3	ION 1-3 Combined	ION 4
N (GT 1)	213	109	216	538	327
SVR12 (%)	99	94	96	97	96
Relapse rates (%)	<1	6	1	2	3
Discontinuation rates (%)	0	0	1	<1	0

LDV/SOF Drug Interactions with E/C/F/TAF and F/R/TAF {Custodio et al 2015}

Use of anti-HCV agents such as LDV, a P-glycoprotein (P-gp) and BCRP inhibitor, with HIV ARV such as TAF, a P-gp and BCRP substrate, may be complicated by drug-drug interactions (DDIs). Two Phase 1 studies evaluated DDIs between TAF-based regimens F/R/TAF or E/C/F/TAF and the fixed-dose combination anti-HCV regimen of LDV/SOF. In two multiple-dose, randomized, crossover studies, healthy volunteers received R/F/TAF (Study 1) or E/C/F/TAF (Study 2), alone or in combination with LDV/SOF, daily with food for 11 [Study 1] or 10 [Study 2] days. Plasma concentrations of RPV, EVG, COBI, FTC, TAF, TFV (TAF metabolite), LDV, SOF, and GS-331007 (predominant circulating metabolite of SOF) were analyzed and PK parameters calculated via noncompartmental analysis. Geometric least squares mean ratio (GMR; combination vs. alone) and 90% confidence intervals (CI) for analytes' AUC_{tau}, C_{max} and C_{tau} were estimated by linear mixed effect modeling and compared to lack of PK alteration bounds (70-143% except RPV: 80-125%). Safety was assessed throughout the studies.

Forty (40) of 42 subjects (Study 1) and 30/30 subjects (Study 2) completed the study. Treatments were generally well tolerated.

The GMR and 90% CI for all analytes’ PK parameters are:

Table 1-2. Study 1: F/R/TAF and LDV/SOF

PK Parameter	Mean (%CV) F/R/TAF (Treatment B) (N = 42)	Mean (%CV) LDV/SOF+F/R/TAF (Treatment C) (N = 42)	%GLSM Ratio (90% CI)
**FTC**			
AUC_{tau} (h*ng/mL)	10,764.1 (14.3)	10,805.1 (15.3)	100.29 (98.43,102.19)
C_{max} (ng/mL)	1707.6 (20.2)	1650.3 (17.6)	97.02 (92.72,101.53)
C_{tau} (ng/mL)	87.9 (28.2)	88.7 (25.1)	101.59 (98.47,104.81)
**RPV**			
AUC_{tau} (h*ng/mL)	3040.1 (27.3)	2857.6 (25.6)	94.59 (91.20,98.10)
C_{max} (ng/mL)	203.3 (25.4)	197.1 (28.3)	96.65 (91.73,101.84)
C_{tau} (ng/mL)	109.1 (31.6)	100.0 (26.0)	93.33 (89.38,97.45)
**TAF**			
AUC_{last} (h*ng/mL)	277.2 (37.5)	362.3 (34.4)	132.39 (124.99,140.22)
C_{max} (ng/mL)	200.0 (43.5)	204.5 (45.7)	103.12 (93.58,113.63)
**TFV**			
AUC_{tau} (h*ng/mL)	268.4 (22.6)	467.2 (21.0)	174.72 (168.78,180.86)
C_{max} (ng/mL)	15.8 (21.7)	25.4 (20.0)	161.50 (155.60,167.62)
C_{tau} (ng/mL)	9.0 (24.8)	16.7 (22.0)	184.86 (177.57,192.46)

PK Parameter	Mean (%CV)		%GLSM Ratio (90% CI)
	LDV/SOF (Treatment A) (N = 41)	LDV/SOF+F/R/TAF (Treatment C) (N = 42)	
**SOF**			
AUC_{tau} (h*ng/mL)	2909.4 (32.8)	3068.9 (30.5)	104.69 (100.51,109.04)
C_{max} (ng/mL)	1469.5 (35.4)	1390.6 (32.3)	95.99 (88.80,103.76)
**GS-331007**			
AUC_{tau} (h*ng/mL)	11,766.4 (12.8)	12,883.3 (16.1)	107.98 (106.20,109.79)
C_{max} (ng/mL)	884.4 (13.7)	960.4 (14.8)	108.09 (105.05,111.20)
C_{tau} (ng/mL)	339.8 (16.4)	378.1 (18.9)	109.92 (107.46,112.44)
**LDV**			
AUC_{tau} (h*ng/mL)	11,590.4 (40.3)	11,944.8 (42.7)	101.53 (97.36, 105.88)
C_{max} (ng/mL)	647.4 (35.8)	658.4 (37.7)	100.62 (96.76, 104.63)
C_{tau} (ng/mL)	419.7 (45.9)	434.3 (47.4)	102.40 (98.02, 106.99)

Table 1-3. Study 2: E/C/F/TAF and LDV/SOF PK Parameter

	Mean (%CV)		%GLSM Ratio (90% CI)
	E/C/F/TAF (N = 30)	LDV/SOF + E/C/F/TAF (N = 30)	
**EVG**			
AUC_{tau} (h*ng/mL)	26,888.6 (22.74)	29,750.7 (23.26)	110.62 (102.40, 119.50)
C_{max} (ng/mL)	2184.3 (23.29)	2155.3 (27.49)	97.85 (89.57, 106.91)
C_{tau} (ng/mL)	513.3 (40.06)	731.3 (35.57)	145.70 (127.99, 165.86)
**COBI**			
AUC_{tau} (h*ng/mL)	11,354.2 (36.34)	17,046.0 (30.36)	153.17 (144.87, 161.95)
C_{max} (ng/mL)	1381.4 (25.98)	1668.0 (17.14)	123.35 (115.35, 131.91)
C_{tau} (ng/mL)	56.1 (99.59)	175.2 (90.42)	325.08 (287.62, 367.43)
**FTC**			
AUC_{tau} (h*ng/mL)	12,035.7 (15.39)	11,622.9 (15.53)	96.59 (93.22, 100.08)
C_{max} (ng/mL)	1777.0 (18.40)	1840.3 (22.12)	103.04 (95.93, 110.67)
C_{tau} (ng/mL)	103.5 (26.84)	98.5 (27.24)	95.02 (90.82, 99.42)
**TAF**			
AUC_{last} (h*ng/mL)	238.6 (45.87)	194.8 (29.21)	85.67 (77.63, 94.54)
C_{max} (ng/mL)	165.9 (50.79)	148.2 (48.24)	90.45 (73.41, 111.45)
**TFV**			
AUC_{tau} (h*ng/mL)	314.8 (18.68)	396.8 (15.90)	126.61 (122.55, 130.81)
C_{max} (ng/mL)	17.8 (20.43)	20.7 (16.14)	116.85 (111.71, 122.23)
C_{tau} (ng/mL)	11.7 (20.05)	15.5 (16.62)	132.89 (127.74, 138.25)

PK Parameter	Mean (%CV)		%GLSM Ratio (90% CI)
	LDV/SOF FDC (N = 30)	LDV/SOF + E/C/F/TAF (N = 30)	
**LDV**			
AUC_{tau} (h*ng/mL)	12,657.2 (36.26)	22,939.3 (35.71)	178.99 (163.62, 195.81)
C_{max} (ng/mL)	684.2 (32.25)	1136.0 (32.14)	164.74 (152.60, 177.86)
C_{tau} (ng/mL)	459.2 (40.34)	896.1 (37.74)	193.16 (173.91, 214.55)
**SOF**			
AUC_{tau} (h*ng/mL)	2336.2 (37.40)	3308.4 (24.84)	146.53 (135.44, 158.53)
C_{max} (ng/mL)	1220.3 (37.87)	1573.2 (34.88)	128.48 (112.51, 146.72)
**GS-331007**			
AUC_{tau} (h*ng/mL)	12,611.4 (20.91)	18,739.0 (22.60)	148.15 (143.54, 152.91)
C_{max} (ng/mL)	950.9 (18.74)	1228.7 (18.57)	129.36 (123.92, 135.04)
C_{tau} (ng/mL)	358.7 (24.66)	599.5 (27.40)	165.96 (159.61, 172.57)

GLSM and 90% CIs for all analytes were contained within the prespecified bounds except: i) TFV when RPV/FTC/TAF was administered with LDV/SOF; ii) COBI, when E/C/F/TAF was coadministered with LDV/SOF; and iii) LDV, GS-331007 and SOF and EVG when administered with E/C/F/TAF.

Despite an increase in TFV exposure upon coadministration of R/F/TAF + LDV/SOF, the mean TFV AUC_{tau} is ~ 5 times lower than TFV from TDF, and within the range of TFV that did not lead to renal AEs or bone loss (E/C/F/TAF Phase 3). There is no association between higher COBI exposure and incidence of AEs, renal function parameters (E/C/F/TAF Phase 2/3 data). Higher plasma exposures of LDV, SOF and GS-331007 in the presence of E/C/F/TAF are not considered to be clinically important based on the established exposure-safety analyses for LDV/SOF. Thus, F/R/TAF or E/C/F/TAF may be coadministered with LDV/SOF without dose modification.

1.4. Rationale for This Study

Currently, LDV/SOF is approved by the US FDA for treatment of HCV genotype 1- infected HCV mono and HIV co-infected patients. This Phase 3 open-label study is designed to extend this data to examine the efficacy and safety of LDV/SOF treatment regimen for chronic genotype 1 HCV infection in HIV-infected patients who are receiving HIV therapy with E/C/F/TAF or F/R/TAF. Current guidelines for treatment of HCV in HIV co-infected patients emphasize the added need to treat co-infected patients due to higher rates of liver disease progression even if the HIV infection is treated with antiretroviral agents. The complex drug interactions that can occur between HIV ARV and HCV DAA further the need for safe, simple regimens that cure HCV in HIV-infected subjects without threatening the underlying HIV suppression. Current

IDSA/AASLD guidelines recommend that “Antiretroviral drug switches may be performed to allow compatibility of DAAs, with the goal of maintaining HIV suppression without compromising future options.” {HCV Guidelines 2015} Prior HIV treatment history, underlying HIV drug resistance and tolerability of regimens needs to be considered.

LDV/SOF has been used successfully to treat HIV/HCV co-infected patients and the cure rates are 96%, comparable to HCV mono-infected patients {Naggie et al 2015}, which has led to a recent update to the LDV/SOF package insert that now includes HIV/HCV co-infection in the list of indications {Gilead Sciences Inc 2015}. However, few ARV regimens were evaluated in clinical studies of HCV treatment of HIV co-infected subjects. Only RAL, EFV and RPV were used in the ION-4 study with FTC/TDF as the dual NRTI component of the regimen. In the presence of a boosting agent, such as RTV or COBI (which increase plasma TFV concentrations), further increases in plasma TFV concentrations are noted when coadministered with LDV/SOF + TDF. Guidelines recommend avoiding RTV or COBI-boosted ARV regimens with TDF when treating HCV in HIV-infected patients, mainly due to lack of data. The availability of TAF-based regimens will obviate the problem of excessive TFV concentrations due to the 91% lower plasma TFV levels when TAF is used in place of TDF. The PK drug interaction studies (GS-US-366-1689 and Study 2) further justify the ability to use LDV/SOF in HIV-infected patients who are receiving TAF-based regimens (including COBI).

Drug-drug interactions of LDV/SOF coadministered with E/C/F/TAF and F/R/TAF were evaluated in healthy volunteers; the studies found no significant interactions and concluded that LDV/SOF and TAF-regimens can be coadministered without dose modification. The duration of the healthy volunteer drug-interaction studies were 10 and 11 days. Although, the US package insert for Genvoya® (E/C/F/TAF) suggests there should be no clinically significant drug interaction with LDV/SOF, there is no study to date that has evaluated the safety or efficacy of LDV/SOF administered with E/C/F/TAF or F/R/TAF for treatment of HCV in suppressed, HIV co-infected population. Careful assessment of safety/tolerability of LDV/SOF with TAF regimens for 12 weeks is needed to ensure that the safety data, noted in healthy volunteers PK studies, is replicated during 12 weeks of coadministration in HIV/HCV co-infected subjects. The goal of this study is to evaluate E/C/F/TAF FDC or F/R/TAF FDC with LDV/SOF by determining the efficacy of HCV therapy (SVR 12 weeks after completion of LDV/SOF) and safety and tolerability of coadministration of dual STRs.

1.5. Risk/Benefit Assessment for the Study

The risk/benefit for this study is acceptable due to the following considerations: i) treatment of HCV is indicated and important in all HIV co-infected patients due to accelerated progression of liver disease; ii) 12 weeks of LDV/SOF provides high HCV SVR rates in HIV/HCV co-infected subjects; iii) changing ARV regimens is an accepted strategy for management of patients prior to initiation of HCV therapy (AASLD/ IDSA Guidelines); iv) changing to E/C/F/TAF from other ART has been shown to be safe, effective and have superior treatment outcome than maintaining a current suppressive regimen {Mills et al 2015}; v) F/R/TAF is bioequivalent to approved HIV regimens and achieves plasma concentrations of FTC, RPV and TAF that have proven to be safe, well tolerated and effective in maintaining virologic suppression in HIV-infected patients. Thus, for individual subjects there is benefit to treating their HCV and for all co-infected patients, finding safe, tolerable, efficacious and simple regimens will be a major advance in treatment.

1.6. Compliance

This study will be conducted in compliance with this protocol, Good Clinical Practice (GCP), and all applicable regulatory requirements.

2. OBJECTIVES

The primary objective of this study is:

- To evaluate efficacy of LDV/SOF as measured by the proportion of subjects achieving HCV RNA below the lower limit of quantification (LLOQ) 12 weeks after the last dose of LDV/SOF (SVR 12).

The secondary objectives of this study are:

- To determine the proportion of subjects achieving HCV RNA below LLOQ 4 weeks after the last dose of LDV/SOF (SVR 4);
- To evaluate maintenance of HIV-1 RNA suppression after switching to E/C/F/TAF or F/R/TAF 24 weeks from the start of the F/TAF-based regimen;
- To evaluate the safety and tolerability of switching to E/C/F/TAF or F/R/TAF from the current ARV therapy in virologically-suppressed, HIV-1/HCV co-infected subjects;
- To evaluate the safety and tolerability of 12 weeks of treatment for HCV with LDV/SOF in virologically-suppressed, HIV-1/HCV co-infected subjects who switched to E/C/F/TAF or F/R/TAF.

3. STUDY DESIGN

3.1. Endpoints

The primary endpoint of this study is:

- The primary efficacy endpoint is SVR12 (HCV RNA < LLOQ 12 weeks after completion of LDV/SOF treatment)

The secondary endpoints of this study are:

- SVR4 (HCV RNA < LLOQ 4 weeks after completion of LDV/SOF treatment);
- The proportion of subjects with HIV-1 RNA ≥ 50 copies/mL (virologic failure) 24 weeks after start of the F/TAF-based regimen using modified FDA snapshot algorithm;
- Grades 1 through 4 adverse events after switching to E/C/F/TAF or F/R/TAF throughout the study and during coadministration with LDV/SOF treatment.

3.2. Study Design

This protocol describes a randomized, multicenter, open-label, 2-part study to evaluate the efficacy, safety, and tolerability of switching to E/C/F/TAF FDC or F/R/TAF FDC HIV treatments and adding LDV/SOF HCV treatment for 12 weeks in adult male and female subjects with HIV who are co-infected with chronic GT 1 HCV infection.

3.3. Study Treatments

Subjects will participate in 2 parts of the study. In Part 1, subjects will be randomized in a 1:1 ratio to one of the following 2 HIV treatment groups:

Part 1: Approximately 240 subjects will be randomized 1:1 to switch from stable ARV therapy to one of the following treatments. Randomization will be stratified by race (black vs. non-black) at screening.

- **Treatment Group 1:** Switch from 2 NRTI plus a third ARV agent to E/C/F/TAF FDC (n = 120)
- **Treatment Group 2:** Switch from 2 NRTI plus a third ARV agent to F/R/TAF FDC (n = 120)

Subjects maintaining HIV-1 RNA < 50 copies/mL and tolerating E/C/F/TAF or F/R/TAF through Part 1 will continue to Part 2 where they will receive the HCV treatment. Subjects with confirmed virologic failure or who discontinue the HIV study drugs due to toxicity in Part 1 will be discontinued from the study.

Part 2: After 8 weeks on the randomized E/C/F/TAF FDC or F/R/TAF FDC treatment, subjects will maintain their HIV treatment and will initiate 12 weeks of LDV/SOF HCV treatment.

At the end of their LDV/SOF HCV treatment, subjects will continue to maintain their HIV treatment and be followed for safety and efficacy for an additional 12 weeks to determine the SVR12 endpoint and HIV safety and efficacy endpoints.

3.4. Duration of Treatment

The HIV treatment duration is from the Day 1 visit until the last study visit at the Post-HCV Treatment Week 12. The HCV treatment duration is 12 weeks. Screening assessments will be completed within 42 days of the Day 1 visit.

All subjects will complete the following study visits: Screening, Day 1, Weeks 4, 6 (laboratory visit only), 8 (start of HCV treatment), 12, 16, and 20 (end of HCV treatment), and post-HCV treatment visits 4 and 12 weeks after the last LDV/SOF dose.

Subjects who complete the study through the last study visit at the Post-HCV Treatment Week 12 visit will be required to return to the clinic 30 days after the completion of the study treatment for a 30-Day Follow-Up visit.

Figure 3-1. Study Schema

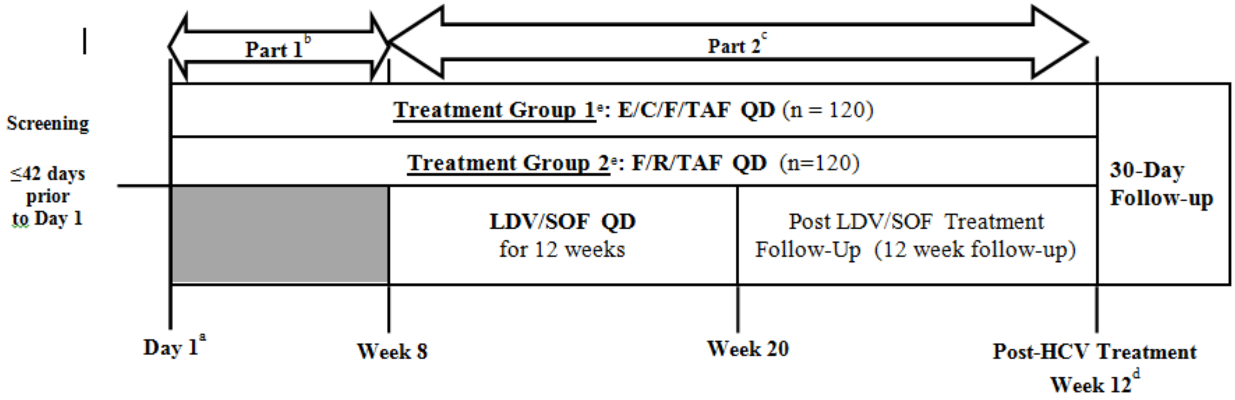

a Following the Day 1 visit, subjects will return for study visits at Weeks 4, 6 (laboratory visit only), 8, 12, 16, 20, and Post-HCV Treatment 4 and 12 weeks after the last LDV/SOF dose.
b Part 1: subjects are randomized to either E/C/F/TAF FDC or F/R/TAF FDC on Day 1 and maintain HIV treatment to the Week 8 visit.
c Part 2: At Week 8, once HIV-1 RNA suppression (< 50 copies/mL) is confirmed (based on the Week 6 HIV-1 RNA value), subjects will start on LDV/SOF FDC for 12 weeks (until Week 20) while maintaining their HIV treatment. At Week 20, LDV/SOF FDC will be discontinued while continuing HIV treatment. Subjects will continue in the study for another 12 weeks to determine SVR4 (4 weeks post-HCV treatment) and SVR12 (12 weeks post-HCV treatment).
d Subjects who complete the study through the Post-HCV Treatment Week 12 visit will be required to return to the clinic 30 days after the completion of the study treatment for a 30-Day Follow-Up Visit.
e At the Day 1 visit, subjects will switch to either E/C/F/TAF FDC or F/R/TAF FDC and continue on HIV treatment until the last study visit at the Post-HCV Treatment Week 12 visit.

3.5. Samples for Optional Exploratory Assessments

PPD [redacted]

[redacted]

[redacted]

[redacted]

4. SUBJECT POPULATION

4.1. Number of Subjects and Subject Selection

Approximately 240 subjects with HIV-1 who are coinfected with chronic HCV infection and who meet the eligibility criteria will be enrolled. In order to manage the total study enrollment, Gilead Sciences, Inc., at its sole discretion, may suspend screening and/or enrollment at any site or study-wide at any time.

4.2. Inclusion Criteria

Subjects must meet *all* of the following inclusion criteria to be eligible for participation in this study.

1) The ability to understand and sign a written informed consent form, which must be obtained prior to initiation of any study procedures.

2) Age ≥ 18 years.

3) Currently on an ARV regimen (2 NRTI + a third agent, see Table 4-1) without change for 6 months prior to screening. Documented plasma HIV-1 RNA levels < 50 copies/mL (or undetectable HIV-1 RNA level according to the local assay being used if the limit of detection is ≥ 50 copies/mL) for ≥ 6 months preceding the Screening visit. After reaching HIV-1 RNA < 50 copies/mL, single values (“blips”) of HIV-1 RNA ≥ 50 copies/mL followed by resuppression are allowed. For subjects with 3 or more prior ARV regimens, a regimen history should be provided for approval by the Sponsor.

   Note: Subjects that changed from TDF to TAF less than 6 months ago will be eligible as long as the TDF/ TAF change was the only change to the regimen

Table 4-1. Allowable ARV Agents of Pre-Existing HIV Regimen

Antiretroviral Class	Agents
Boosted PI	ATV+COBI (or ATV/COBI FDC), darunavir (DRV)+COBI (or DRV/COBI FDC), DRV+RTV, lopinavir (LPV)/RTV, ATV+RTV, fosamprenavir (FPV)+RTV, FPV+COBI, SQV+RTV, SQV+COBI, IDV+RTV, IDV+COBI, ATV (no booster)
NNRTI	EFV, etravirine (ETR), nevirapine (NVP), RPV
NRTI	FTC, TDF, TAF, abacavir (ABC), lamivudine (3TC), zidovudine (AZT)
INSTI	dolutegravir (DTG), RAL, EVG/COBI or EVG+RTV

4) No history of HIV virologic failure

5) If the current regimen is a PI/r, the previous regimen cannot contain an NNRTI or INSTI

6) Plasma HIV-1 RNA level < 50 copies/mL at the Screening visit.

7) Have no documented resistance to any of the HIV study agents at any time in the past, including but not limited to the reverse transcriptase resistance mutations K65R, K70E, K101E/P, E138A/G/K/R/Q, V179L, Y181C/I/V, M184V/I, Y188L, H221Y, F227C, M230I/L, the combination of K103N+L100I, or 3 or more TAMs that include M41L or L210W (TAMs are M41L, D67N, K70R, L210W, T215Y/F, K219Q/E/N/R). If a historical genotype prior to first ARV is not available or subject has 3 or more prior ARV regimens, subject will have proviral genotype analysis for archived resistance prior to Day 1 (all proviral genotype reports must be reviewed and approved by Sponsor).

8) Chronic HCV infection (≥ 6 months) documented by prior medical history.

9) HCV genotype 1 at screening as determined by the Central Laboratory. Any non-definitive genotype results will exclude the subject from study participation.

10) Classification as one of the following criteria:

    a) Without cirrhosis and no prior HCV treatment (**treatment-naïve**, defined as having never been exposed to approved or investigational HCV-specific direct-acting antiviral agents or prior treatment of HCV with IFN and RBV)

    b) Without cirrhosis and have received HCV treatment only with IFN +/- RBV +/- HCV PI (**treatment-experienced**, defined as prior treatment failure to a regimen containing IFN either with or without RBV that was completed at least 8 weeks prior to Day 1. The subject's medical records must include sufficient detail of prior treatment experience for categorization, as either:

        i. Non-Responder: Subject did not achieve undetectable HCV RNA levels while on treatment, or

        ii. Relapse/Breakthrough: Subject achieved undetectable HCV RNA levels during treatment or within 4 weeks of the end of treatment but did not achieve SVR, or

        iii. HCV Treatment-Intolerant: Subjects who discontinued HCV treatment of a regimen containing IFN (either with or without RBV) due to toxicity

    c) Compensated cirrhosis and no prior HCV treatment (treatment-naïve)

11) Cirrhosis Determination

    a) Presence of cirrhosis is defined as any one of the following:

        i. FibroTest® score > 0.75 AND an AST:platelet ratio index (APRI) > 2 during screening

        ii. Fibroscan with a result of > 12.5 kPa

        iii. Liver biopsy showing cirrhosis (eg, Metavir score = 4 or Ishak score ≥ 5)

    b) Absence of cirrhosis is defined as any one of the following:

        i. FibroTest® score ≤ 0.48 AND APRI ≤ 1 performed during screening

ii. Fibroscan with a result of ≤ 12.5 kPa within ≤ 6 months of Day 1

iii. Liver biopsy within 2 years of screening showing absence of cirrhosis

In the absence of a definitive diagnosis of presence or absence of cirrhosis by Fibrotest® /APRI using the above criteria, a liver biopsy or fibroscan is required. Liver biopsy results will supersede Fibrotest® /APRI or fibroscan results and be considered definitive.

12) Liver imaging within 6 months of Day 1 is required in cirrhotic subjects only to exclude hepatocellular carcinoma (HCC)

13) HCV RNA ≥ 10^4 IU/mL at the Screening visit

14) Normal ECG (or if abnormal, determined by the Investigator to be not clinically significant)

15) Adequate renal function: estimated GFR ≥ 30 mL/min according to the Cockcroft-Gault formula ($eGFR_{CG}$) for creatinine clearance {Cockcroft et al 1976}:

Male: $$\frac{(140 - \text{age in years}) \times (\text{wt in kg})}{72 \times (\text{serum creatinine in mg/dL})} = CL_{cr} \text{ (mL/min)}$$

Female: $$\frac{(140 - \text{age in years}) \times (\text{wt in kg})}{72 \times (\text{serum creatinine in mg/dL})} \times 0.85 = CL_{cr} \text{ (mL/min)}$$

16) Hepatic transaminases (AST and ALT) ≤ 5 × upper limit of normal (ULN)

17) Total bilirubin ≤ 1.5 mg/dL, **and** direct bilirubin ≤ 1.5 x ULN (subjects with documented Gilbert's syndrome or with atazanavir-associated hyperbilirubinemia may have total bilirubin up to 5 × ULN as long as direct bilirubin is ≤ 1.5 x ULN.)

18) Adequate hematologic function:

- Absolute neutrophil count ≥ 1,000/mm^3
- Platelets ≥ 50,000/mm^3
- Hemoglobin ≥ 8.5 g/dL

19) Albumin ≥ 3.0 g/dL

20) INR ≤ 1.5 x ULN unless subject has known hemophilia or is stable on an anticoagulant regimen affecting INR

21) A female subject is eligible to enter the study if it is confirmed that she is:

a) Not pregnant or nursing

b) Of non-childbearing potential (ie, women who have had a hysterectomy, have had both ovaries removed or medically documented ovarian failure, or are postmenopausal women ≥ 54 years of age with cessation [for ≥ 12 months] of previously occurring menses), or

c) Of childbearing potential and agrees to utilize protocol-specified contraceptive methods or be non-heterosexually active or practice sexual abstinence from screening throughout the duration of study treatment and for 30 days following the last study drug dose (see Appendix 5 for definitions and protocol-acceptable contraceptive methods)

22) Male subjects must agree to utilize a protocol-approved method of contraception (as defined in Appendix 5) during heterosexual intercourse or be non-heterosexually active, or practice sexual abstinence from first dose throughout the study period and for 30 days following the last study drug dose.

23) Male subjects must agree to refrain from sperm donation from first dose until at least 30 days after the last study drug dose.

4.3. Exclusion Criteria

Subjects who meet *any* of the following exclusion criteria are not to be enrolled in this study.

1) A new AIDS-defining condition diagnosed within the 30 days prior to the Screening visit (except CD4+ cell count and/or percentage criteria) (refer to Appendix 6)

2) Prior HCV treatments with NS5A and NS5B or any HCV directly acting antivirals except boceprevir, telaprevir and simeprevir in combination with IFN +/- RBV.

3) Chronic liver disease of a non-HCV etiology (e.g., hemochromatosis, Wilson’s disease, alfa-1 antitrypsin deficiency, cholangitis).

4) History or current decompensation of liver disease; varices at low risk of bleeding are allowed.

5) Positive Hepatitis B surface antigen (HBsAg) or Hepatitis B virus (HBV) DNA

6) Chronic use of systemically administered immunosuppressive agents (eg, prednisone equivalent > 10 mg/day).

7) Females who are breastfeeding

8) Positive serum pregnancy test

9) Have an implanted defibrillator or pacemaker

10) Current alcohol or substance use judged by the Investigator to potentially interfere with subject study compliance

11) A history of malignancy within the past 5 years (prior to screening) or ongoing malignancy other than cutaneous Kaposi's sarcoma (KS), basal cell carcinoma, or resected, non-invasive cutaneous squamous carcinoma. Subjects with cutaneous KS are eligible, but must not have received any systemic therapy for KS within 42 days of Day 1visit and must not be anticipated to require systemic therapy during the study.

12) Active, serious infections (other than HIV-1 and HCV infections) requiring parenteral antibiotic or antifungal therapy within 42 days prior to Day 1

13) Gastrointestinal disorder or post-operative condition that could interfere with the absorption of the study drugs

14) Solid organ transplantation

15) Significant pulmonary disease or significant cardiac disease

16) Any other clinical condition or prior therapy that, in the opinion of the Investigator, would make the subject unsuitable for the study or unable to comply with dosing requirements

17) Participation in any other clinical trial (including observational trials) without prior approval from the sponsor is prohibited while participating in this trial

18) Known hypersensitivity to the study drugs, the metabolites, or formulation excipients

19) Subjects receiving ongoing therapy with any of the medications in Table 4-2, including drugs not to be used due to the potential for interaction with HIV study drugs (COBI, EVG, FTC, RPV, or TAF. For COBI, EVG, FTC, or RPV, refer to the individual agents' Prescribing Information; for TAF, refer to the E/C/F/TAF FDC or F/R/TAF FDC Investigator's Brochures) or HCV study drugs (LDV or SOF); or subjects with any known allergies to the excipients of E/C/F/TAF, F/R/TAF, or LDV/SOF tablets.

Table 4-2. Disallowed Agents[a]

Drug Class	Agents Disallowed[b]
Acid Reducing Agents	Proton pump inhibitors, such as Esomeprazole, Lansoprazole, Omeprazole, Pantoprazole, Rabeprazole
Alpha Adrenergic Receptor Antagonists	Alfuzosin
Antiarrythmics	Amiodarone[d], Quinadine
Anticonvulsants[c]	Phenobarbital, Phenytoin, Carbamazepine, Oxcarbazepine
Antimycobacterials[c]	Rifampin, Rifapentine, Rifabutin
Calcium Channel Blockers	Bepridil
Corticosteroids: Systemic	Dexamethasone (more than a single dose)

**Drug Class**	**Agents Disallowed**[b]
Ergot Derivatives	Ergotamine, Ergonovine<br>Dihydroergotamine<br>Methylergonovine<br>Ergometrine
GI Motility Agents	Cisapride
Herbal/Natural Supplements[c]	St. John's Wort, Echinacea, Milk thistle (ie, silymarin), Chinese herb sho-saiko-to (or Xiao-Shai-Hu-Tang)
HMG-CoA Reductase Inhibitors	Simvastatin, Lovastatin, Rosuvastatin[e]
Inhaled Beta Agonist	Salmeterol
Neuroleptics	Pimozide
Phosphodiesterase-5 Inhibitors	Sildenafil (for PAH)
Sedatives/Hypnotics	Orally administered Midazolam, Triazolam

a. Disallowed agents are inclusive of all HIV and HCV study drugs (E/C/F/TAF, F/R/TAF, and LDV/SOF).
b. Administration of any of the following medications must be discontinued at least 42 days prior to the Day 1 visit and for the duration of the study, with the exception of amiodarone (see footnote d).
c. May result in a decrease in the concentrations of all HIV and HCV study drugs.
d. May result in symptomatic bradycardia. Mechanism is currently unknown. The use of amiodarone is prohibited from 60 days prior to the Day 1 visit and for the duration of the study.
e. Use with LDV/SOF may result in an increase in the concentration of rosuvastatin, which is associated with increased risk of myopathy including rhabdomyolysis.

5. INVESTIGATIONAL MEDICINAL PRODUCTS

5.1. Randomization

Subjects will be assigned a screening number at the time of consent. Once eligibility has been confirmed, each subject will be assigned a unique subject number. Once a subject number has been assigned to a subject, it will not be reassigned to any other subject. Prior to or during the Day 1 visit, the Investigator or designee will randomize the subject using the Interactive Web Response System (IWRS). The subject number assignment and randomization may be performed up to 7 days prior to the in-clinic Day 1 visit provided that all screening procedures have been completed and subject eligibility has been confirmed.

Subjects will be randomized in a 1:1 ratio to one of the following two treatment arms:

Treatment Arm 1: Switch from 2 NRTI plus a third ARV agent to E/C/F/TAF FDC (n = 120)

Treatment Arm 2: Switch from 2 NRTI plus a third ARV agent to F/R/TAF FDC (n = 120)

Randomization will be stratified by race (black vs. non-black).

The IWRS will assign study drug bottle numbers to be dispensed to the subject at each study visit, for all HIV and HCV study drugs. **All Day 1 assessments and procedures must be completed prior to the administration of the first dose of HIV study drug.** Initiation of treatment with the HIV study drug must take place within 24 hours after the Day 1 visit.

5.2. Description and Handling of E/C/F/TAF, F/R/TAF, and LDV/SOF

5.2.1. Formulation

5.2.1.1. E/C/F/TAF, Genvoya®

E/C/F/TAF tablets are capsule-shaped, film-coated green tablets debossed with "GSI" on one side of the tablet and "510" on the other side. Each E/C/F/TAF tablet contains 150 mg of EVG, 150 mg of COBI, 200 mg of FTC, and 10 mg of TAF (as 11.2 mg of TAF fumarate).

In addition to the active ingredients, the tablets contain silicon dioxide, croscarmellose sodium, hydroxypropyl cellulose, lactose monohydrate, magnesium stearate, microcrystalline cellulose, and sodium lauryl sulfate as inactive ingredients and are film-coated with indigo carmine aluminum lake, polyethylene glycol, polyvinyl alcohol, talc, titanium dioxide, and yellow iron oxide.

5.2.1.2. F/R/TAF, Odefsey®

F/R/TAF tablets are capsule-shaped, film-coated gray tablets debossed with "GSI" on one side and "255" on the other side. Each F/R/TAF tablet contains 200 mg of FTC, 25 mg of RPV (as 27.5 mg RPV hydrochloride), and 25 mg of TAF (as 28 mg of TAF fumarate).

In addition to the active ingredients, the tablets contain lactose monohydrate, microcrystalline cellulose, polysorbate, povidone, croscarmellose sodium and magnesium stearate. The tablet cores are film-coated with polyvinyl alcohol, titanium dioxide, polyethylene glycol, talc, and black iron oxide.

5.2.1.3. LDV/SOF, Harvoni®

LDV/SOF tablets are orange, diamond-shaped, film-coated tablets debossed with “GSI” on one side and “7985” on the other side. Each LDV/SOF FDC tablet contains 90 mg of LDV and 400 mg of SOF.

In addition to the active ingredients, the tablets contain lactose monohydrate, copovidone, microcrystalline cellulose, croscarmellose sodium, colloidal silicon dioxide, magnesium stearate, polyvinyl alcohol, titanium dioxide, talc, polyethylene glycol, and FD&C yellow # 6 /sunset yellow FCF aluminium lake.

5.2.2. Packaging and Labeling

Study drug(s) to be distributed to centers in the US and other participating countries shall be labeled to meet applicable requirements of the United States Food and Drug Administration (FDA) and/or other local regulations.

5.2.2.1. E/C/F/TAF, Genvoya®

E/C/F/TAF tablets are packaged in white, high density polyethylene (HDPE) bottles. Each bottle contains 30 tablets, silica gel desiccant, and polyester packing material. Each bottle is enclosed with a white, continuous thread, child-resistant polypropylene screw cap with an induction-sealed, and aluminum-faced liner. Commercially available product of Genvoya® may also be used for the study.

5.2.2.2. F/R/TAF, Odefsey®

F/R/TAF tablets are packaged in white, high density polyethylene (HDPE) bottles. Each bottle contains 30 tablets, silica gel desiccant, and polyester packing material. Each bottle is enclosed with a white, continuous thread, child-resistant polypropylene screw cap with an induction-sealed, and aluminum-faced liner. Commercially available product of Odefsey® may also be used for the study.

5.2.2.3. LDV/SOF, Harvoni®

LDV/SOF tablets are packaged in white, high density polyethylene (HDPE) bottles. Each bottle contains 28 or 30 tablets, silica gel desiccant and polyester packing material. Each bottle is enclosed with a white, continuous thread, child-resistant polypropylene screw cap with an induction-sealed, and aluminum-faced liner. Commercially available product of Harvoni® may also be used for the study.

5.2.3. Storage and Handling

E/C/F/TAF, F/R/TAF, and LDV/SOF tablets should be stored at controlled room temperature of 25 °C (77 °F); excursions are permitted between 15 °C and 30 °C (59 °F and 86 °F). Storage conditions are specified on the label. Until dispensed to the subjects, all bottles of study drug should be stored in a securely locked area, accessible only to authorized site personnel.

To ensure the stability and proper identification, study drugs should not be stored in a container other than the container in which they were supplied. Consideration should be given to handling, preparation, and disposal through measures that minimize drug contact with the body. Appropriate precautions should be followed by avoiding direct eye contact or exposure when handling.

Commercial product of Genvoya®, Odefsey®, and Harvoni® may be used for the study. Further information regarding storage and handling are available in the Prescribing Information for commercial products.

5.3. Dosage and Administration of E/C/F/TAF, F/R/TAF, and LDV/SOF

E/C/F/TAF FDC, F/R/TAF FDC, and LDV/SOF FDC tablets will be provided by Gilead Sciences.

The two HIV study treatments will be administered orally, once daily with food at approximately the same time each day:

- Elvitegravir 150 mg/cobicistat 150 mg/emtricitabine 200 mg/tenofovir alafenamide 10 mg (E/C/F/TAF) FDC
- Emtricitabine 200 mg/rilpivirine 25 mg/tenofovir alafenamide 25 mg (F/R/TAF) FDC

HCV study treatment (ledipasvir 90 mg/sofosbuvir 400 mg [LDV/SOF] FDC) is to be administered orally, once daily with or without food at approximately the same time each day.

For a missed dose of E/C/F/TAF FDC, F/R/TAF FDC, and/or LDV/SOF FDC tablet, subjects should be instructed to take the missed dose of study drug as soon as possible during the same day. Subjects should be cautioned never to double the next dose with a missed dose of study drug under any circumstances.

5.4. Prior and Concomitant Medications

The use of medications for the treatment of HIV and HCV, other than the study treatments (ie, E/C/F/TAF, F/R/TAF, or LDV/SOF), is prohibited.

Should subjects have a need to initiate treatment with any excluded concomitant medication, the Gilead Medical Monitor must be consulted prior to initiation of the new medication. In instances where an excluded medication is initiated prior to discussion with the Medical Monitor, the Investigator must notify Gilead as soon as he/she is aware of the use of the excluded medication.

Concomitant medications taken within 30 days prior to the Screening visit and up to and including 30 days after the last dose of study drug need to be recorded in the source documents and electronic case report form(s) (eCRFs).

Medications listed in Table 5-1 (E/C/F/TAF + LDV/SOF) and Table 5-2 (F/R/TAF + LDV/SOF) and use of herbal/natural supplements are excluded or should be used with caution while subjects are participating in the study due to potential drug-drug interactions.

Table 5-1. Prior and Concomitant Medications – E/C/F/TAF + LDV/SOF

Drug Class	Agents Disallowed	Use With Caution
Acid Reducing Agents<br>Antacids[a]		Elvitegravir plasma concentrations are lower with antacids due to local complexation in the GI tract and not to changes in gastric pH. It is recommended to separate study drug and antacid administration by at least 2 hours.<br><br>Proton- Pump Inhibitors, H2-Receptor Antagonists, Antacids
Alpha Adrenergic Receptor Antagonists	Alfuzosin	
Analgesics		Tramadol, Propoxyphene:<br>Concentrations may increase with study drug(s); clinical monitoring is recommended.
Antiarrhythmics	Amiodarone[c], Quinadine	Flecainide Quinidine, Propafenone, Systemic Lidocaine, Mexiletine, Disopyramide:<br>Concentrations may increase with study drug(s) resulting in a potential for cardiac arrhythmias; clinical and ECG monitoring is recommended.
Antibacterials		Concentrations of clarithromycin and/or cobicistat may be altered when clarithromycin is coadministered with study drug(s).<br>Patients with CL_{cr} greater than or equal to 60 mL/min:<br>No dose adjustment of clarithromycin is required.<br>Patients with CL_{cr} between 30 mL/min and 60 mL/min:<br>The dose of clarithromycin should be reduced by 50%.<br>Concentrations of telithromycin and/or cobicistat may be increased when telithromycis in coadministered with study drug. Clinical monitoring is recommended upon coadministration with study drug.
Anticoagulants		Warfarin: Concentrations may increase or decrease with study drug(s); appropriate INR (International Normalized Ratio) monitoring is recommended.

Drug Class	Agents Disallowed	Use With Caution
Anticonvulsants[b]	Phenobarbital, Phenytoin, Carbamazepine, Oxcarbazepine	Ethosuximide, Divalproex, Lamotrigine: Concentrations may increase with study drug(s); clinical monitoring is recommended.
Antidepressants		Selective serotonin reuptake inhibitors (SSRIs): A dose reduction may be required for most drugs of this class with the exception of sertraline.<br><br>Tricyclics: Concentrations may increase or decrease with study drug(s). Concentration monitoring is recommended to ensure adequate clinical response.<br><br>Trazodone: Concomitant use with CYP3A inhibitors results in increased concentrations and adverse events; dose reduction should be considered.
Antifungals		Ketoconazole and Itraconazole: Concomitant use with study drug may result in an increase in concentrations. Daily dose of ketoconazole and itraconazole should be restricted to 200 mg. Subjects receiving ketoconazole or itraconazole should be monitored for adequate clinical response.<br><br>Voriconazole: Concomitant use with study drug may result in an increase in concentrations. Clinical monitoring may be needed.
Antigout		Dose reductions of colchicine may be required. Study drug(s) should not be coadministered with colchicine in patients with renal or hepatic impairment.
Antihistamines		Concentrations of astemizole and terfenadine may be increased when coadministered with cobicistat. Clinical monitoring is recommended when these agents are coadministered with study drug(s).
Antimycobacterials[b]	Rifampin, Rifapentine, Rifabutin	
β-Blockers		Metoprolol, Timolol: Clinical and ECG monitoring of subjects is recommended. A dose decrease may be needed.
Calcium Channel Blockers	Bepridil	Felodipine, Nifedipine, Nicardipine, Verapamil, Diltiazem, Amlodipine: Concentrations may increase with study drug(s). Clinical and ECG monitoring of subjects is recommended
Cardiac Medications		Digoxin[d]

Drug Class	Agents Disallowed	Use With Caution
Corticosteroids: Inhaled/Nasal		Concomitant use of inhaled fluticasone and study drug(s) may increase plasma concentrations of fluticasone. Use is not recommended unless the potential benefit to the subject outweighs the risks of corticosteroid side effects. Alternatives should be considered, particularly for long-term use.
Corticosteroids: Systemic		Systemic dexamethasone, a CYP3A inducer, may significantly decrease elvitegravir and cobicistat plasma concentrations, which may result in loss of therapeutic effect and development of resistance. Alternative corticosteroids should be considered.<br>Use of Prednisone as a steroid burst (≤ 1 week of use) should be monitored appropriately.
Endothelin Receptor Antagonists		Bosentan:<br>Coadministration may lead to decreased elvitegravir exposures and loss of therapeutic effect and development of resistance. Alternative endothelin receptor antagonists may be considered.
Ergot Derivatives	Ergotamine, Ergonovine<br>Dihydroergotamine<br>Methylergonovine<br>Ergometrine	
GI Motility Agents	Cisapride	
Herbal/Natural Supplements[b]	St. John's Wort, Echinacea, Milk thistle (ie, silymarin), Chinese herb sho-saiko-to (or Xiao-Shai-Hu-Tang)	
HMG-CoA Reductase Inhibitors	Simvastatin, Lovastatin, Rosuvastatin[e]	Atorvastatin:<br>Concentrations may increase with study drug(s). Start with the lowest dose; gradual increase in dose may be tailored to clinical response. Careful monitoring for signs and symptoms of muscle weakness or myopathy, including rhabdomyolysis.
Immunosuppressants		Cyclosporine, Rapamycin, Sirolimus, Tacrolimus:<br>Concentrations may increase with study drug(s). Therapeutic monitoring should be considered.
Inhaled Beta Agonist	Salmeterol	
Neuroleptics	Pimozide	Perphenazine, Risperidone, Thioridazine:<br>A dose decrease may be needed.

Drug Class	Agents Disallowed	Use With Caution
Opiates		Methadone:<br>Methadone exposures are unaffected upon coadministration with elvitegravir and cobicistat. No dose adjustment of methadone is required upon coadministration with study drug(s).<br><br>Meperidine (Pethidine):<br>Dosage increase and long-term use are not recommended due to increased levels of metabolite normeperidine, which has analgesic and CNS stimulant (eg, seizures) activities.<br><br>Buprenorphine:<br>Concentrations of buprenorphine and norbuprenorphine are modestly increased and concentrations of naloxone are modestly decreased when coadministered with elvitegravir and cobicistat, with no effect on opioid pharmacodynimcs. The concentration changes are not considered clinically relevant and no dose adjustment of buprenorphine/naloxone is required upon coadministration with study drug(s).
Phosphodiesterase-5 Inhibitors	Sildenafil (for PAH)	<u>Pulmonary Arterial Hypertension</u>:<br><br>Tadalafil:<br>Caution should be exercised, including consideration of dose reduction, when coadministered for treatment of pulmonary arterial hypertension.<br><br><u>Erectile Dysfunction</u>:<br><br>Sildenafil, Vardenafil, Tadalafil:<br>It is recommended that a single dose of Sildenafil no more than 25 mg in 48 hours, Vardenafil no more than 2.5 mg in 72 hours, or Tadalafil no more than 10 mg in 72 hours be coadministered.
Sedatives/Hypnotics	Orally administered Midazolam, Triazolam	Buspirone, Clorazepate, Diazepam, Estazolam, Flurazepam, Zolpidem:<br>A dose decrease may be needed for these drugs. Clinical monitoring is recommended.

a It is recommended to separate antacid and LDV/SOF administration by 4 hours. H2-receptor antagonists may be administered simultaneously with or staggered from LDV/SOF at a dose that does not exceed doses comparable to famotidine 40 mg twice daily. Proton-pump inhibitor doses comparable to omeprazole 20 mg can be administered simultaneously with study drugs. Proton-pump inhibitors should not be taken before study drugs.

b May result in a decrease in the concentrations of study drugs.

c May result in symptomatic bradycardia. Mechanism is currently unknown. The use of amiodarone is prohibited from 60 days prior to Day 1 through the end of treatment

d Coadministration of E/C/F/TAF or LDV/SOF with digoxin may increase the concentration of digoxin. Caution is warranted and therapeutic concentration monitoring of digoxin is recommended when coadministered with E/C/F/TAF or LDV/SOF.

e Use with study drug may result in an increase in the concentration of rosuvastatin, which is associated with increased risk of myopathy including rhabdomyolysis

Table 5-2. Prior and Concomitant Medications – F/R/TAF + LDV/SOF

Drug Class	Agents Disallowed	Use With Caution
Acid Reducing Agents[a]	Proton pump inhibitors, such as Esomeprazole, Lansoprazole, Omeprazole, Pantoprazole, Rabeprazole	H2-Receptor Antagonists, Antacids Cimetidine, Famotidine, Nizatidine, Ranitidine<br><br>Coadministration of H2-receptor antagonists (H2RA) may cause significant decreases in RPV plasma concentrations (increase in gastric pH). H2RAs should only be administered at least 12 hours before or at least 4 hours after F/R/TAF
Alkaline antacids: antacids (eg, aluminum or magnesium hydroxide, calcium carbonate)		Antacids should be used with caution as coadministration may cause significant decreases in RPV plasma concentrations (increase in gastric pH). Antacids should only be administered either at least 2 hours before or at least 4 hours after F/R/TAF.
Antiarrhythmics	Amiodarone[c], Quinidine	
Anticonvulsants[b]	Carbamazepine, Oxcarbazepine, Phenobarbital, Phenytoin	
Antifungals		Fluconazole, Itraconazole, Ketoconazole, Posaconazole, Voriconazole: may increase concentration of RPV.
Antimycobacterials[b]	Rifapentine, Rifabutin, Rifampin	
Cardiac Medications		Digoxin[d]
Corticosteroids: Systemic	Dexamethasone (more than a single dose)	
Herbal/Natural Supplements[b]	St. John's Wort, Echinacea, Milk thistle (ie, silymarin), Chinese herb Sho-Saiko-To (or Xiao-Shai-Hu-Tang)	
HMG-CoA Reductase Inhibitors[e]	Rosuvastatin	
Narcotic analgesics		Methadone<br>Clinical monitoring is recommended as methadone maintenance therapy may need to be adjusted in some patients upon coadministration with RPV

a It is recommended to separate antacid and LDV/SOF administration by 4 hours. H2-receptor antagonists may be administered simultaneously with or staggered from LDV/SOF at a dose that does not exceed doses comparable to famotidine 40 mg twice daily. Proton-pump inhibitor doses comparable to omeprazole 20 mg can be administered simultaneously with study drugs. Proton-pump inhibitors should not be taken before study drugs.

b May result in a decrease in the concentrations of study drugs.

c May result in symptomatic bradycardia. Mechanism is currently unknown. The use of amiodarone is prohibited from 60 days prior to Day 1 through the end of treatment

d Coadministration of F/R/TAF or LDV/SOF with digoxin may increase the concentration of digoxin. Caution is warranted and therapeutic concentration monitoring of digoxin is recommended when co-administered with F/R/TAF or LDV/SOF.

e Use with study drug may result in an increase in the concentration of rosuvastatin, which is associated with increased risk of myopathy including rhabdomyolysis.

5.5. Accountability for Investigational Medicinal Product (IMP)

The investigator is responsible for ensuring adequate accountability of all used and unused IMP. This includes acknowledgement of receipt of each shipment of IMP (quantity and condition). All used and unused IMP dispensed to subjects must be returned to the site.

IMP accountability records will be provided by Gilead Sciences (or equivalent documentation maintained by the study site) to:

- Record the date received and quantity of IMP bottles
- Record the date, subject number, subject initials, the IMP bottle number dispensed
- Record the date, quantity of used and unused IMP returned, along with the initials of the person recording the information.

5.5.1. Investigational Medicinal Product Return or Disposal

IMP return and disposal will be performed as outlined in Section 9.1.7.

6. STUDY PROCEDURES

The study procedures to be conducted for each subject enrolled in the study are presented in tabular form in Appendix 2 and described in the text that follows. Additional information is provided in the study procedures manual.

The investigator must document any deviation from protocol procedures and notify Gilead or contract research organization (CRO).

6.1. Subject Enrollment and Treatment Assignment

It is the responsibility of the Investigator to ensure that each subject is eligible for the study before enrollment. Please refer to Section 5.1 for details about randomization and treatment assignment.

6.2. Pretreatment Assessments

6.2.1. Screening Visit

Subjects will be screened within 42 days prior to the Day 1 visit to determine eligibility for participation in the study. The following assessments will be completed and documented at screening:

- Obtain written informed consent
- Determine eligibility including cirrhosis determination (see Section 4.2 for details)
  - If the presence of cirrhosis is determined, then appropriate diagnostic imaging (CT or Ultrasound) should be performed within 6 months of Day 1 to exclude the presence of hepatocellular carcinoma
- Obtain medical history, including:
  - History of HIV-1 disease-related events and prior medications within 30 days of the Screening visit. For subjects with 3 or more prior ARV regimens, a regimen history should be provided for approval by the Sponsor.
  - Collection of historical HIV genotype, if available. If a historical genotype prior to first ARV is not available or subject had 3 or more prior ARV regimens, subject will have proviral genotype analysis for archived resistance prior to Day 1 (all proviral genotype reports must be reviewed and approved by Sponsor).
  - If HCV treatment-experienced, record the duration of the prior treatment and the type of IFN +/- RBV + HCV PI administered. Record whether the subject had a Non-Response or Relapse/Breakthrough during prior treatment or was Treatment Intolerant.
    - Non-Response: Subject did not achieve undetectable HCV RNA while on treatment.

- Relapse/Breakthrough: Subject achieved undetectable HCV RNA during treatment or within 4 weeks of the end of treatment, but did not achieve SVR
- Treatment Intolerant: Subject discontinued HCV treatment of a regimen containing IFN either with or without RBV due to toxicity

- Complete physical examination (urogenital/anorectal exams will be performed at the discretion of the Investigator)
- Obtain vital signs measurement (blood pressure, pulse, respiration rate, and temperature)
- Obtain 12-lead ECG (performed supine)
- Obtain height and weight
- Obtain urine samples for the following laboratory analyses:
  - Urinalysis and urine chemistry
- Obtain blood samples for the following laboratory analyses:
  - Serum pregnancy test (females of childbearing potential only). If the test is positive, the subject will not be enrolled.
  - Chemistry profile: alkaline phosphatase, AST, ALT, total bilirubin, direct and indirect bilirubin, total protein, albumin, bicarbonate, BUN, calcium, chloride, creatinine, glucose, phosphorus, magnesium, potassium, sodium, and uric acid.
  - Hematology profile: complete blood count (CBC) with differential and platelet count
  - Coagulation assessments: International Normalized Ratio (INR), prothrombin time (PT), and activated partial thromboplastin time (APTT)
  - CD4+ cell count
  - Plasma HIV-1 RNA (COBAS® TaqMan® HIV RNA Test, v2.0)
  - Whole blood sample for HIV proviral genotype analysis of archived resistance (if a historical genotype prior to first ARV is <u>not available</u> or subject had 3 or more prior ARV regimens)
  - Plasma HCV RNA (COBAS® TaqMan® HCV Quantitative Test, v2.0)
  - HCV genotype and subtype, and IL28B genotype testing

- Serology for:
  - HIV antibody
  - HBV core antibody (HBcAb), surface antibody (HBsAb), surface antigen (HBsAg), e-antibody (HBeAg), e-antibody (HBeAb), and HBV DNA
  - HCV antibody

- Obtain estimated glomerular filtration rate according to the Cockcroft-Gault formula (see Section 4.2 for details)
- Obtain details of adverse events related to screening procedures
- Review concomitant medications

Subjects meeting all of the inclusion criteria and none of the exclusion criteria will return to the clinic within 42 days after the Screening visit for the Day 1 assessments.

From the time of obtaining informed consent through the first administration of investigational medicinal product, record all serious adverse events (SAEs), as well as any adverse events (AE) related to protocol-mandated procedures on the adverse events electronic case report form (eCRF). All other untoward medical occurrences observed until dosing of study drugs, including exacerbation or changes in medical history are to be captured on the medical history eCRF. See Section 7, Adverse Events and Toxicity Management, for additional details.

6.2.2. Day 1 Assessments

The following assessments are to be completed at the Day 1 visit. The Investigator must have received the results from the screening assessments before proceeding with the visit. Subjects must complete all Day 1 procedures before being dispensed the study drugs. Initiation of treatment with HIV study drugs must take place within 24 hours after the Day 1 visit.

- Review AEs and changes in concomitant medications
- Complete physical examination (urogenital/anorectal exams will be performed at the discretion of the Investigator)
- Obtain vital signs, including weight
- Obtain urine samples for the following laboratory analyses:
  - Urinalysis and urine chemistry
  - Urine pregnancy test (females of childbearing potential only). If the urine pregnancy test is positive at Day 1, study drugs will not be dispensed. The positive result will be confirmed with a serum pregnancy test. If the serum pregnancy test is positive, the subject will not be able to participate.

- Renal tubular function assessments (collected fasted, no food or drink, except water, at least 8 hours prior to blood collection): retinol binding protein, beta-2 microglobulin, urine albumin, and urine protein
- Urine sample storage for possible additional clinical testing

- Obtain blood samples for the following laboratory analyses:
  - Chemistry profile
  - Hematology profile
  - Coagulation assessments
  - Metabolic assessments (collected fasted, no food or drink, except water, at least 8 hours prior to blood collection): glucose and lipid panel (total cholesterol, HDL, direct LDL, and triglycerides).
    - If the subject has not fasted prior to the visit, the visit may proceed, but the subject must return within 72 hours in a fasted state to draw blood for the metabolic assessments.
  - CD4+ cell count
  - Plasma HIV-1 RNA
  - Plasma HCV RNA
  - Serum bone safety assessments: (collected fasted, no food or drink, except water, at least 8 hours prior to blood collection) parathyroid hormone (PTH) and serum 25-OH Vitamin D
    - If the subject has not fasted prior to the visit, the visit may proceed, but the subject must return within 72 hours in a fasted state to draw blood for these assessments.
  - Platelet and coagulation function assessments may include: Soluble glycoprotein VI (sGPVI), P-selectin, soluble CD40 ligand, and D-dimer
  - Inflammation assessments may include: cystatin-C, IL 6, hs CRP, sCD14, sCD163, sTNF-R1, and Lp-PLA2
  - Plasma storage samples for virology, safety, and/or PK testing
  - Serum storage sample for possible additional clinical testing
  - PPD

- Obtain estimated glomerular filtration rate according to the Cockcroft-Gault formula (see Section 4.2 for details)

- Complete questionnaires: subject is to read questionnaire by her/himself and write/mark answers directly onto the questionnaires.

  - Adherence for HIV (VAS)
  - HIV Treatment Satisfaction Questionnaire – Status version (HIVTSQs)
  - Medical Outcomes Short Form-36 (SF-36)
  - Functional Assessment of Chronic Illness Therapy-Fatigue scale (FACIT-F)
  - Chronic Liver Disease Questionnaire (CLDQ-HCV)
  - Work Productivity and Activity Impairment Questionnaire: Hepatitis C, v2.0 (WPAI: Hepatitis C)

6.3. Randomization

- Obtain subject number and randomize the subject via the IWRS. The subject number assignment and randomization may be performed up to 7 days prior to the in-clinic Day 1 visit provided that all screening procedures have been completed and subject eligibility has been confirmed.

- **Dispense HIV study drugs**. Subjects should initiate dosing of HIV study drugs after Day 1 visit assessments. Initiation of treatment with the study drugs must take place within 24 hours after the Day 1 visit.

  - Subjects should also be counseled regarding the importance of adherence and taking their study drug daily with food at approximately the same time each day.

6.4. Treatment Assessments

6.4.1. HIV and HCV Treatment Visits (Weeks 4, 6, 8, 12, 16, and 20)

The following assessments are to be completed at the end of Weeks 4, 8, 12, 16, and 20, unless otherwise specified. The Week 6 visit is a laboratory visit only – please see Section 6.4.1.1 for details.

All study visits are to be scheduled relative to the Day 1 visit date. Study visits are to be completed within ± 2 days of the protocol-specified visit date based on the Day 1 visit through Week 20.

- Review AEs and changes in concomitant medications

- Perform symptom-directed physical examination as needed

- Obtain vital signs, including weight
- Obtain urine samples for the following laboratory analyses:
  - Urinalysis and urine chemistry
  - Urine pregnancy test (females of childbearing potential only); positive urine pregnancy tests will be confirmed with a serum test. If the serum test is positive, the subject will be discontinued.
  - Renal tubular function assessments **(Weeks 4, 8, 12, and 20 only)**
  - Urine sample storage for possible additional clinical testing
- Obtain blood samples for the following laboratory analyses:
  - Chemistry profile
  - Hematology profile
  - Coagulation assessments
  - Metabolic assessments **(Weeks 8 and 20 only)**
  - CD4+ cell count
  - Plasma HIV-1 RNA
  - Plasma HCV RNA **(Weeks 8, 12, 16, and 20 only)**
  - Samples for population PK **(Weeks 4, 8, 12, 16, and 20)**
    - Two (2) blood samples will be collected: 1) the predose sample should be drawn prior to observed study drug dosing; 2) the postdose sample should be drawn between 15 mins and 4 hours postdose.
  - Serum bone safety assessments **(Weeks 4, 8, 12, and 20 only)**
  - Platelet and coagulation function assessments **(Weeks 4, 8, 12, and 20 only)**
  - Inflammation assessments **(Weeks 4, 8, 12, and 20 only)**
  - Plasma storage samples for virology, safety, and/or PK testing
  - Serum storage sample for possible additional clinical testing
  - PPD [redacted] **)**

- Obtain estimated glomerular filtration rate according to the Cockcroft-Gault formula (see Section 4.2 for details)
- Complete questionnaires **(Weeks 8 and 20 only)**: subject is to read questionnaire by her/himself and write/mark answers directly onto the questionnaires.
  - VAS for HIV adherence
  - HIVTSQc – Change version
  - SF-36
  - FACIT-F
  - CLDQ-HCV
  - WPAI: Hepatitis C
- Subjects who meet the criteria for virologic rebound should be managed according to Management of HIV Virologic Failure (Section 6.9)
- Document study drugs dispensation and accountability for all study drugs dispensed. Counsel all subjects regarding the importance of adherence and maintaining consistency to study drug administration.
  - **HIV study drugs are dispensed at every visit (with the exception of Week 6 laboratory visit).**
  - HCV treatment will commence at Week 8, after determination of HIV suppression (< 50 copies/mL) based on the Week 6 HIV-1 RNA value and tolerability of HIV study drugs. Once confirmed, **dispense HCV study drug (LDV/SOF) at Weeks 8, 12, and 16 only**.

6.4.1.1. Week 6 (Laboratory Visit Only)

The Week 6 visit is a laboratory visit only, to confirm HIV suppression prior to initiation of HCV treatment.

The following will be performed and documented at the Week 6 visit:

- Obtain blood samples for plasma HIV-1 RNA testing
- Plasma storage sample for safety and virology testing

6.4.2. HIV Treatment and Post-HCV Treatment Assessments (Post-HCV Treatment Weeks 4 and 12)

The following evaluations are to be completed at the end of 4 and 12 weeks after completion of HCV treatment (Post-HCV Treatment Weeks 4 and 12 visits, respectively) and should be timed from the date of last administration of the HCV treatment. The Post-HCV Treatment Week 4 visit is to be completed ± 2 days, and the Post-HCV Treatment Week 12 visit is to be completed within ± 4 days; however, unless notified by Gilead Sciences, the Post-HCV Treatment Week 12 should be completed within 4 days of the protocol-specified visit date.

All subjects must complete the Post-HCV Treatment Weeks 4 and 12 visits. The end of study will occur at the Post-HCV Treatment Week 12 visit.

The following assessments are to be completed:

- Review AEs and changes in concomitant medications
- Complete physical examination **(Post-HCV Treatment Week 12 only)** (urogenital/anorectal exams will be performed at the discretion of the Investigator)
- Perform symptom-directed physical examination as needed
- Obtain vital signs, including weight
- Obtain urine samples for the following laboratory analyses:
  - Urinalysis and urine chemistry
  - Urine pregnancy test (females of childbearing potential only); positive urine pregnancy tests will be confirmed with a serum test. If the serum test is positive, the subject will be discontinued.
  - Renal tubular function assessments **(Post-HCV Treatment Week 12 only)**
  - Urine sample storage for possible additional clinical testing
- Obtain blood samples for the following laboratory analyses:
  - Chemistry profile
  - Hematology profile
  - Coagulation assessments
  - Metabolic assessments **(Post-HCV Treatment Week 12 only)**
  - CD4+ cell count

- Plasma HIV-1 RNA
- Plasma HCV RNA
- Serum bone safety assessments **(Post-HCV Treatment Week 12 only)**
- Platelet and coagulation function assessments **(Post-HCV Treatment Week 12 only)**
- Inflammation assessments **(Post-HCV Treatment Week 12 only)**
- Plasma storage samples for virology, safety, and/or PK testing
- Serum storage sample for possible additional clinical testing
- PPD [redacted]

- Obtain estimated glomerular filtration rate according to the Cockcroft-Gault formula (see Section 4.2 for details)

- Complete questionnaires **(Post-HCV Treatment Week 12 only)**: subject is to read questionnaire by her/himself and write/mark answers directly onto the questionnaires.
  - VAS for HIV adherence
  - HIVTSQc – Change version
  - SF-36
  - FACIT-F
  - CLDQ-HCV
  - WPAI: Hepatitis C

- Subjects who meet the criteria for virologic rebound should be managed according to Management of HIV Virologic Failure (Section 6.9)

- Document study drugs dispensation and accountability for all **HIV study drugs** dispensed. Counsel all subjects regarding the importance of adherence and maintaining consistency to HIV study drug administration.

6.5. Post-treatment Assessments

6.5.1. Early Study Drug Discontinuation (ESDD) Visit

Early study drug discontinuation during Part 1 (Day 1 through Week 8):

If the subject discontinues HIV study drug dosing in Part 1, the subject will be discontinued from the study and will not continue to Part 2 of the study. The subject will be asked to return to the clinic within 72 hours of stopping HIV study drugs for the ESDD visit, followed by the 30-Day Follow-Up visit.

Early study drug discontinuation during Part 2 (after initiation of HCV treatment):

If the subject discontinues HCV study drug dosing only prior to completing the 12-week HCV treatment, the following will occur:

- Be asked to return to the clinic within 72 hours of stopping HCV study drug for the ESDD visit.
- Remain in the study to return for follow-up visits 4 and 12 weeks after the last dose of LDV/SOF, as described in Section 6.4.2.
- Continue to receive HIV study drugs (see ESDD procedures below).

If the subject discontinues HIV study drug dosing only, the following will occur:

- Be asked to return to the clinic within 72 hours of stopping HIV study drugs for the ESDD visit.
- HIV regimen may be changed, after discussion with the Medical Monitor. The new HIV regimen should be designed to allow continued dosing of LDV/SOF. LDV/SOF should be continued unless an HIV regimen cannot be constructed that would allow coadministration with the new HIV regimen.
- Remain in the study to complete the 12-week HCV treatment (see Section 6.4.1) and follow-up visits 4 and 12 weeks after the last dose of LDV/SOF, as described in Section 6.4.2.

If the subject discontinues both HIV and HCV study drug dosing, the following will occur:

- Be asked to return to the clinic within 72 hours of stopping study drugs for the ESDD visit.
- Remain in the study to return for follow-up visits 4 and 12 weeks after the last dose of LDV/SOF, as described in Section 6.4.2.

At the ESDD Visit, any evaluations showing abnormal results indicating that <u>there is a possible or probable causal relationship with the study drugs</u>, should be repeated weekly (or as often as deemed prudent by the Investigator) until the abnormality is resolved, returns to baseline, or is otherwise explained.

The following evaluations are to be completed at the ESDD Visit:

- Review of AEs and changes in concomitant medications
- Complete physical examination (urogenital/anorectal exams will be performed at the discretion of the Investigator)
- Obtain vital signs, including weight
- Obtain 12-lead ECG (performed supine)
- Obtain urine samples for the following laboratory analyses:
  - Urinalysis and urine chemistry
  - Urine pregnancy test (females of childbearing potential only); positive urine pregnancy tests will be confirmed with a serum test.
  - Urine sample storage for possible additional clinical testing
- Obtain blood samples for the following laboratory analyses:
  - Chemistry profile
  - Hematology profile
  - CD4+ cell count
  - Plasma HIV-1 RNA
  - Plasma HCV RNA
  - Plasma storage samples for virology, safety, and/or PK testing
  - PPD
- Obtain estimated glomerular filtration rate according to the Cockcroft-Gault formula (see Section 4.2 for details)

- Complete questionnaires: subject is to read questionnaire by her/himself and write/mark answers directly onto the questionnaires.
  - VAS for HIV adherence
  - HIVTSQc – Change version
  - SF-36
  - FACIT-F
  - CLDQ-HCV
  - WPAI: Hepatitis C
- HIV-1 genotype/phenotype resistance testing for subjects with unconfirmed virologic rebound with HIV-1 RNA value ≥ 400 copies/mL.
- Perform study drug accountability:
  - For subjects that **discontinue during Part 1 of the study**, perform study drug accountability on HIV study drugs.
  - For subjects that **discontinue during Part 2 of the study**, perform study drug accountability for HIV and HCV study drugs.
    - If the subject discontinues HCV study drug and is staying on HIV study drug, dispense HIV study drug until the last study visit.
    - If the subject discontinues HIV study drug, HCV study drug will be dispensed at the scheduled visits as described in Section 6.4.1

6.5.2. 30-Day Follow-Up Visit

Subjects who complete the study through the Post-HCV Treatment Week 12 visit will be required to return to the clinic 30 days after the completion of study for the 30-Day Follow-Up visit.

Subjects who permanently discontinue HIV study drug in Part 1 of the study will be asked to return to the clinic after the completion of the ESDD visit for a 30-Day Follow-Up visit.

A ± 6 days window may be used to schedule this visit. The following evaluations are to be completed at this visit:

- Review of AEs and changes in concomitant medications
- Perform symptom-directed physical examination

- Obtain vital signs measurement, including weight
- Obtain urine samples for the following laboratory analyses:
  - Urinalysis and urine chemistry
  - Urine pregnancy test (females of childbearing potential only); positive urine pregnancy tests will be confirmed with a serum test.
- Obtain blood samples for the following laboratory analyses:
  - Chemistry profile
  - Hematology profile
  - CD4+ cell count
  - Plasma HIV-1 RNA
  - Plasma HCV RNA

At the 30-Day Follow-Up Visit, any evaluations showing abnormal results indicating that there is a reasonable possibility of a causal relationship with the study drug will be repeated weekly (or as often as deemed prudent by the Investigator) until the abnormality is resolved, returns to baseline, or is otherwise explained.

6.6. Assessments for Premature Discontinuation from Study

If a subject discontinues study dosing (for example, as a result of an AE), every attempt should be made to keep the subject in the study and continue to perform the required study-related follow-up and procedures (refer to Appendix 2). If this is not possible or acceptable to the subject or investigator, the subject may be withdrawn from the study and asked to return for the ESDD Visit followed by the 30-Day Follow-Up Visit.

6.7. Criteria for Discontinuation of Study Treatment

Study medication may be discontinued in the following instances:

- Intercurrent illness that would, in the judgment of the Investigator, affect assessments of clinical status to a significant degree.
- Unacceptable toxicity, as defined in the toxicity management section of the protocol, or toxicity that, in the judgment of the Investigator, compromises the ability to continue study-specific procedures or is considered to not be in the subject's best interest
- Lack of efficacy (virologic failure)

- Subject request to discontinue for any reason
- Subject noncompliance
- Pregnancy during the study; refer to Appendix 5
- Discontinuation of the study at the request of Gilead Sciences, a regulatory agency or an institutional review board or independent ethics committee (IRB/IEC)

6.8. Other Assessments

6.8.1. Blood and Urine Storage

A portion of the blood drawn and urine collected at all visits (except Screening, Week 6, 30-Day Follow-Up, or Unscheduled) will be frozen and stored. From subjects who provide additional consent, a portion of the blood draw at Day 1, Weeks 4, 8, 20, Post-HCV Treatment Week 12, and ESDD visit will be frozen and stored. These stored samples may be used by Gilead or our research partners to help answer questions about the study drug, HIV-1 infection and its associated conditions, or clinical laboratory testing to provide additional safety data. No human genetic testing will be performed without express consent of the study subjects. At the conclusion of this study, these samples may be retained in storage for Gilead for a period of up to 15 years.

6.8.2. Bone Safety and Inflammation

For all subjects, blood will be collected for selected assessments of bone safety, including serum 25-OH Vitamin D and PTH. Assessments for inflammation may include: cystatin-C, IL 6, hs CRP, sCD14, sCD163, sTNF-R1, and Lp-PLA2. Collections will be made at Day 1, Weeks 4, 8, 12, 20, and Post-HCV Treatment Week 12.

6.8.3. Markers of Platelet Function and Coagulation

For all subjects, blood will be collected for selected assessments of platelet function and coagulation that may include: soluble glycoprotein VI (sGPVI), P-selectin, soluble CD40 ligand and D-dimer. Collections will be made at Day 1, Weeks 4, 8, 12, 20, and Post-HCV Treatment Week 12.

6.8.4. Markers of Renal Tubular Function

For all subjects, urine will be collected for selected assessments of renal tubular function that may include retinol binding protein, beta-2 microglobulin, urine albumin, and urine protein. Collections will be made at Day 1, Weeks 4, 8, 12, 20, and Post-HCV Treatment Week 12.

6.9. HIV Virologic Failure

Subjects who experience virologic rebound (VR), as defined below, will be considered to have virologic failure.

Subjects will be considered to have virologic rebound if at any visit has an HIV-1 RNA value ≥ 50 copies/mL confirmed at the next scheduled or unscheduled visit (2 to 4 weeks later).

6.9.1. Management of HIV Virologic Rebound

The management of virologic rebound during Part 1 and Part 2 are different; subjects that have confirmed virologic failure during Part 1 will be discontinued from the study and not begin Part 2, while subjects that have virologic failure in Part 2 will be managed as described below.

<u>Management of HIV-1 RNA ≥ 50 copies/mL during Part 1 (Day 1 through Week 8)</u>

- All HIV-1 RNA values ≥ 50 copies/mL should be discussed with the Medical Monitor.
- If the viral load at any visit is ≥ 50 copies/mL, HIV-1 RNA should be repeated at a scheduled or unscheduled visit 2 to 4 weeks after the date of the original test with HIV-1 RNA ≥ 50 copies/mL.
- Upon confirmation of HIV-1 RNA ≥ 50 copies/mL, potential causes of virologic failure should be documented:
  - Adherence
  - Concomitant medication
  - Comorbidities (eg, active substance abuse, depression, other intercurrent illnesses)
- If virologic rebound is confirmed at the scheduled or unscheduled visit and the HIV-1 RNA value is ≥ 400 copies/mL, the blood samples from that visit will be used for HIV-1 genotype/phenotype testing.
- If virologic failure is confirmed during Part 1 (regardless of detection of HIV drug resistance), the subject should be discontinued from HIV study drug and have the ESDD and 30 Day Follow-Up visit.
- If the viral load at the Week 6 visit is ≥ 50 copies/mL (and this is **not** a confirmation of virologic failure value), the HIV-1 RNA will be repeated at an unscheduled visit in about 2 weeks from the Week 6 visit. This unscheduled visit for confirmation of virologic failure must be completed prior to the Week 8 visit (which subsequently means that the Week 8 visit will be delayed). The subject should not start Part 2 (nor should the subject start LDV/SOF therapy) until the repeat HIV-1 RNA value is available. If the repeat HIV-1 RNA confirms virologic rebound (≥ 50 copies/mL), then the subject will be discontinued from HIV study drugs as above. If the repeat HIV-1 RNA is < 50 copies/mL, then the subject may proceed to Part 2 and maintain the same visit schedule. Initiation of LDV/SOF therapy must occur <u>within 2 to 4 weeks</u> of the original visit window of the Week 8 visit.

<u>Management of HIV-1 RNA ≥ 50 copies/mL during Part 2 (at or after Week 8)</u>

- If the viral load at any visit is ≥ 50 copies/mL, HIV-1 RNA should be repeated at a scheduled or unscheduled visit 2 to 4 weeks after the date of the original test with HIV-1 RNA ≥ 50 copies/mL.
- Upon confirmation of HIV-1 RNA ≥ 50 copies/mL, potential causes of virologic failure should be documented. Assessment should include:
  - Adherence
  - Concomitant medication
  - Comorbidities (eg, active substance abuse, depression, other intercurrent illnesses)
- All subjects with confirmed virologic failure should be discussed with the Medical Monitor.
- If virologic rebound is confirmed at the scheduled or unscheduled visit and the HIV-1 RNA value is ≥ 400 copies/mL, the blood samples from that visit will be used for HIV-1 genotype/phenotype testing.
- If genotypic/phenotypic resistance to study drug is documented, HIV study drugs (E/C/F/TAF or F/R/TAF) should be discontinued.
- If the HIV study drugs (E/C/F/TAF or F/R/TAF) are discontinued, a new HIV regimen should be constructed, after discussion with the Medical Monitor. The new HIV regimen should be designed to allow continued dosing of LDV/SOF. LDV/SOF should be continued unless an HIV regimen cannot be constructed that would allow coadministration with the new HIV regimen.

If no resistance is detected from genotype/phenotype testing, the subject may remain on HIV study drugs and an HIV-1 RNA should be repeated (2 to 4 weeks after date of test with viral load ≥ 50 copies/mL). Investigators should carefully evaluate the benefits and risks of remaining on HIV study drug for each individual subject and document this assessment in the source documents. Investigators who opt to discontinue HIV study drugs for an individual subject must discuss with the Medical Monitor <u>prior</u> to study drug discontinuation.

Please refer to Figure 6-1 for the management of subjects who meet the criteria for virologic rebound.

Figure 6-1. HIV Virologic Rebound Schema

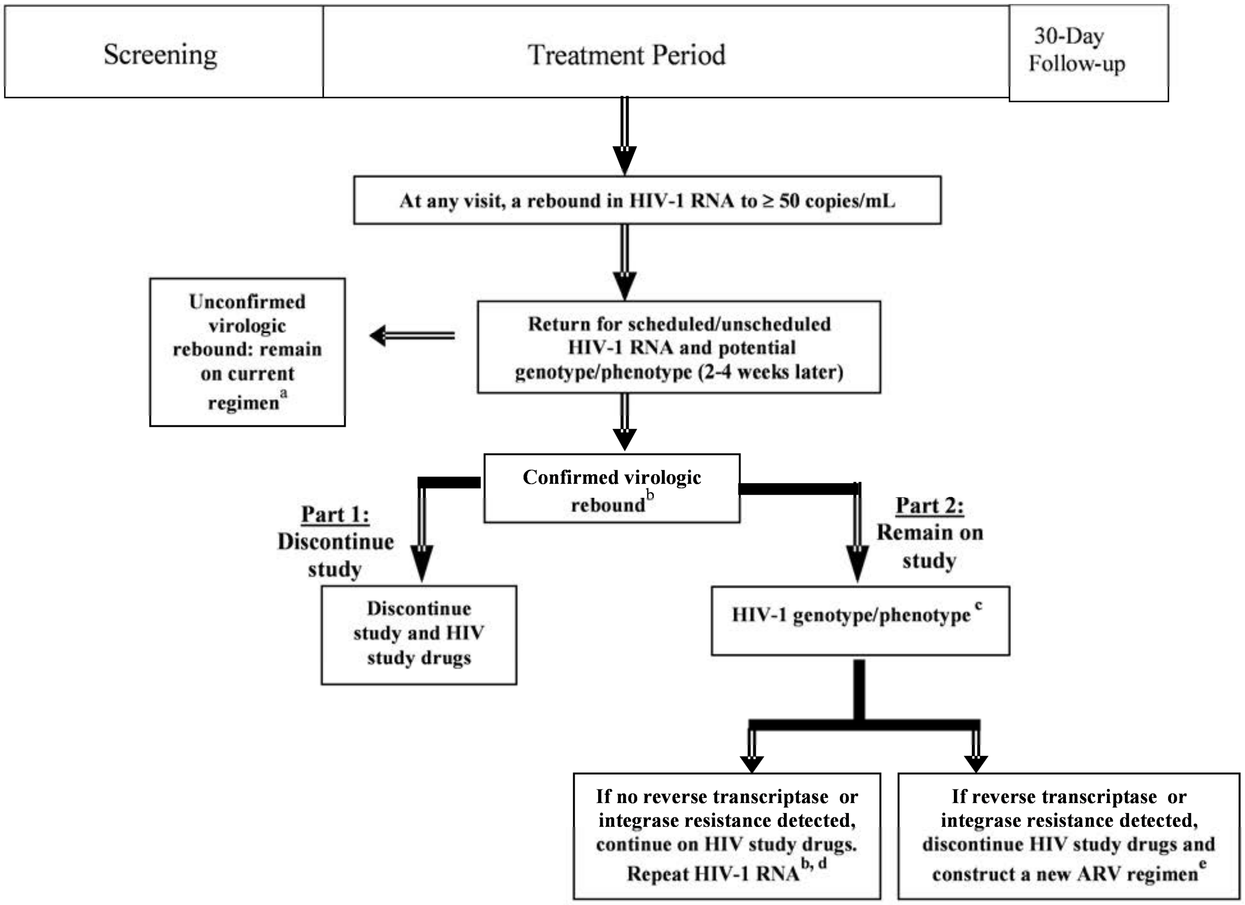

a. If virologic rebound is not confirmed, the subject will remain on their current HIV regimen.
b. If virologic rebound is confirmed, and the HIV-1 RNA is ≥ 400 copies/mL, the HIV-1 genotype and phenotype (reverse transcriptase, integrase, and protease) will be analyzed.
c. Based on the results of the genotype/phenotype assays, the subject will remain on HIV study drugs or study drugs will be discontinued. If genotyping/phenotyping fails, a new ARV regimen may be configured at the discretion of the Investigator.
d. If no resistance detected, HIV-1 RNA will be repeated (2 to 4 weeks later). Investigator reviews HIV study drug continuation/discontinuation options and discuss with Medical Monitor prior to study drug discontinuation.
e. A new ARV regimen will be configured, after discussion with the Medical Monitor. The new HIV regimen should be designed to allow continued dosing of LDV/SOF. LDV/SOF should be continued unless an HIV regimen cannot be constructed that would allow coadministration with the new HIV regimen.

6.9.2. Subjects with ≥ 400 copies/mL of HIV-1 at the Last Study Visit

Subjects with HIV-1 RNA < 50 copies/mL could subsequently experience unconfirmed blips of HIV-1 RNA ≥ 400 copies/mL. Such subjects will be analyzed for resistance if the unconfirmed rebound happens at the Post-HCV Treatment Week 12 visit, or last visit while receiving HIV study drugs (or within 72 hours of discontinuation of study treatment).

6.10. HCV Virologic Response-Based Treatment Stopping Criteria

The following on treatment HCV virologic response-based treatment stopping criteria will be utilized:

- Confirmed > 1 $\log_{10}$ increase from on treatment nadir
- HCV RNA ≥ LLOQ through 8 weeks of treatment

Confirmation should be performed as soon as possible and must occur no later than 2 weeks after an initial observation indicating virologic failure during the HCV on treatment phase.

All subjects who terminate LDV/SOF treatment early will complete the ESDD visit (see Section 6.5.1) and post-HCV treatment follow-up visits at 4 and 12 weeks after the last dose of LDV/SOF (see Section 6.4.2).

6.11. End of Study

End of study is defined as completion of the Post-HCV Treatment Week 12 visit and the 30-Day Follow-Up visit.

6.12. Post Study Care

After a subject has completed/terminated their participation in the study, long-term care for the subject will remain the responsibility of their primary treating physician.

7. ADVERSE EVENTS AND TOXICITY MANAGEMENT

7.1. Definitions of Adverse Events, Adverse Reactions, and Serious Adverse Events

7.1.1. Adverse Events

An **adverse event** (AE) is any untoward medical occurrence in a clinical study subject administered a medicinal product, which does not necessarily have a causal relationship with the treatment. An AE can therefore be any unfavorable and/or unintended sign, symptom, or disease temporally associated with the use of a medicinal product, whether or not considered related to the medicinal product. AEs may also include pre- or post-treatment complications that occur as a result of protocol specified procedures, overdose, drug abuse/misuse reports, or occupational exposure. Preexisting events that increase in severity or change in nature during or as a consequence of participation in the clinical study will also be considered AEs.

An AE does not include the following:

- Medical or surgical procedures such as surgery, endoscopy, tooth extraction, and transfusion. The condition that led to the procedure may be an adverse event and must be reported.
- Pre-existing diseases, conditions, or laboratory abnormalities present or detected before the screening visit that do not worsen
- Situations where an untoward medical occurrence has not occurred (e.g., hospitalization for elective surgery, social and/or convenience admissions)
- Overdose without clinical sequelae (see Section 7.7.1)
- Any medical condition or clinically significant laboratory abnormality with an onset date before the consent form is signed and not related to a protocol-associated procedure is not an AE. It is considered to be pre-existing and should be documented on the medical history CRF.

7.1.2. Serious Adverse Events

A **serious adverse event** (SAE) is defined as an event that, at any dose, results in the following:

- Death
- Life-threatening (Note: The term “life-threatening” in the definition of “serious” refers to an event in which the subject was at risk of death at the time of the event; it does not refer to an event that hypothetically might have caused death if it were more severe.)
- In-patient hospitalization or prolongation of existing hospitalization

- Persistent or significant disability/incapacity
- A congenital anomaly/birth defect
- A medically important event or reaction: such events may not be immediately life-threatening or result in death or hospitalization but may jeopardize the subject or may require intervention to prevent one of the other outcomes constituting SAEs. Medical and scientific judgment must be exercised to determine whether such an event is a reportable under expedited reporting rules. Examples of medically important events include intensive treatment in an emergency room or at home for allergic bronchospasm; blood dyscrasias or convulsions that do not result in hospitalization; and development of drug dependency or drug abuse. For the avoidance of doubt, infections resulting from contaminated medicinal product will be considered a medically important event and subject to expedited reporting requirements.

7.1.3. Clinical Laboratory Abnormalities and Other Abnormal Assessments as Adverse Events or Serious Adverse Events

Laboratory abnormalities without clinical significance are not recorded as AEs or SAEs. However, laboratory abnormalities (eg, clinical chemistry, hematology, and urinalysis) that require medical or surgical intervention or lead to IMP interruption, modification, or discontinuation must be recorded as an AE, as well as an SAE, if applicable. In addition, laboratory or other abnormal assessments (eg, electrocardiogram, x-rays, vital signs) that are associated with signs and/or symptoms must be recorded as an AE or SAE if they meet the definition of an AE or SAE as described in Sections 7.1.1 and 7.1.2. If the laboratory abnormality is part of a syndrome, record the syndrome or diagnosis (eg, anemia), not the laboratory result (ie, decreased hemoglobin).

For specific information on handling of clinical laboratory abnormalities in this study, please refer to Section 7.5.

7.2. Assessment of Adverse Events and Serious Adverse Events

The investigator or qualified subinvestigator is responsible for assessing AEs and SAEs for causality and severity, and for final review and confirmation of accuracy of event information and assessments.

7.2.1. Assessment of Causality for Study Drugs and Procedures

The investigator or qualified subinvestigator is responsible for assessing the relationship to IMP therapy using clinical judgment and the following considerations:

- **No**: Evidence exists that the adverse event has an etiology other than the IMP. For SAEs, an alternative causality must be provided (eg, pre-existing condition, underlying disease, intercurrent illness, or concomitant medication).
- **Yes**: There is reasonable possibility that the event may have been caused by the investigational medicinal product.

It should be emphasized that ineffective treatment should not be considered as causally related in the context of adverse event reporting.

The relationship to study procedures (eg, invasive procedures such as venipuncture or biopsy) should be assessed using the following considerations:

- **No:** Evidence exists that the adverse event has an etiology other than the study procedure.
- **Yes:** The adverse event occurred as a result of protocol procedures, (eg, venipuncture)

7.2.2. Assessment of Severity

Severity should be recorded and graded according to the GSI Grading Scale for Severity of Adverse Events and Laboratory Abnormalities (Appendix 4). For adverse events associated with laboratory abnormalities, the event should be graded on the basis of the clinical severity in the context of the underlying conditions; this may or may not be in agreement with the grading of the laboratory abnormality.

7.3. Investigator Requirements and Instructions for Reporting Adverse Events and Serious Adverse Events to Gilead

Requirements for collection prior to study drug initiation:

After informed consent, but prior to initiation of study medication, the following types of events should be reported on the eCRF: all SAEs and adverse events related to protocol-mandated procedures.

<u>Adverse Events</u>

Following initiation of study medication, collect all AEs, regardless of cause or relationship, until 30 days or 4 weeks after last administration of study IMP and report to the eCRF database as instructed.

All AEs should be followed up until resolution or until the adverse event is stable, if possible. Gilead Sciences may request that certain AEs be followed beyond the protocol defined follow up period.

<u>Serious Adverse Events</u>

All SAEs, regardless of cause or relationship, that occurs after the subject first consents to participate in the study (ie, signing the informed consent) and throughout the duration of the study, including the protocol-required post treatment follow-up period, must be reported to the eCRF database and Gilead Drug Safety and Public Health (DSPH) as instructed. This also includes any SAEs resulting from protocol-associated procedures performed after informed consent is signed.

Investigators are not obligated to actively seek SAEs after the protocol defined follow up period; however, if the investigator learns of any SAEs that occur after study participation has concluded and the event is deemed relevant to the use of IMP, he/she should promptly document and report the event to Gilead DSPH.

- All AEs and SAEs will be recorded in the eCRF database within the timelines outlined in the eCRF completion guideline.

<u>Electronic Serious Adverse Event (eSAE) Reporting Process</u>

- Site personnel record all SAE data in the eCRF database and from there transmit the SAE information to Gilead DSPH within 24 hours of the investigator's knowledge of the event. Detailed instructions can be found in the eCRF completion guidelines.

- If for any reason it is not possible to record the SAE information electronically, ie, the eCRF database is unable to be accessed, record the SAE on the paper serious adverse event reporting form and submit within 24 hours as described above.

GSI Drug Safety and Public Health (DSPH):	Fax:	+1 (650) 522-5477
	E-mail:	safety_fc@gilead.com

- As soon as it is possible to do so, any SAE reported via paper must be transcribed into the eCRF Database according to instructions in the eCRF completion guidelines.

- If an SAE has been reported via a paper form because the eCRF database has been locked, no further action is necessary.

- For fatal or life-threatening events, copies of hospital case reports, autopsy reports, and other documents are also to be submitted by e-mail or fax when requested and applicable. Transmission of such documents should occur without personal subject identification, maintaining the traceability of a document to the subject identifiers.

- Additional information may be requested to ensure the timely completion of accurate safety reports.

- Any medications necessary for treatment of the SAE must be recorded onto the concomitant medication section of the subject's eCRF and the event description section of the SAE form.

- All AEs and SAEs will be recorded in the eCRF database within the timelines outlined in the eCRF completion guideline.

7.4. Gilead Reporting Requirements

Depending on relevant local legislation or regulations, including the applicable US FDA Code of Federal Regulations (CFR), the EU Clinical Trials Directive (2001/20/EC) and relevant updates, and other country-specific legislation or regulations, Gilead may be required to expedite to

worldwide regulatory agencies reports of SAEs, serious adverse drug reactions (SADRs), or suspected unexpected serious adverse reactions (SUSARs). In accordance with the EU Clinical Trials Directive (2001/20/EC), Gilead or a specified designee will notify worldwide regulatory agencies and the relevant IEC in concerned Member States of applicable SUSARs as outlined in current regulations.

Assessment of expectedness for SAEs will be determined by Gilead using reference safety information specified in the IB or relevant local label as applicable.

All investigators will receive a safety letter notifying them of relevant SUSAR reports associated with any study IMP. The investigator should notify the IRB or IEC of SUSAR reports as soon as is practical, where this is required by local regulatory agencies, and in accordance with the local institutional policy.

7.5. Toxicity Management

- All clinical and clinically significant laboratory toxicities relating to HIV ARV agents will be managed according to uniform guidelines detailed in Appendix 3.
- Management for toxicity should be differentiated between ARV and HCV medications.
- Grade 3 and 4 clinically significant laboratory abnormalities should be confirmed by repeat testing within 3 calendar days of receipt of results and before investigational medicinal product discontinuation, unless such a delay is not consistent with good medical practice.
- Clinical events and clinically significant laboratory abnormalities will be graded according to the Table for GSI Grading Scale for Severity of Adverse Events and Laboratory Abnormalities (Appendix 4).
- When restarting investigational medicinal product following resolution of the adverse event, the investigational medicinal product should be restarted at full dose after discussion with the Medical Monitor.
- Any recurrence of the investigational medicinal product-related Grade 3 or 4 clinical or clinically significant laboratory adverse event following dose interruption mandates permanent discontinuation of investigational medicinal product.
- Any questions regarding toxicity management should be directed to the Medical Monitor

7.5.1. Grades 1 and 2 Laboratory Abnormality or Clinical Event

Continue investigational medicinal product at the discretion of the Investigator.

7.5.2. Grade 3 Laboratory Abnormality or Clinical Event

- For Grade 3 clinically significant laboratory abnormality or clinical event, investigational medicinal product may be continued if the event is considered to be unrelated to investigational medicinal product.
- For a Grade 3 clinical event, or clinically significant laboratory abnormality confirmed by repeat testing, that is considered to be related to investigational medicinal product, investigational medicinal product should be withheld until the toxicity returns to ≤ Grade 2.
- If a laboratory abnormality recurs to ≥ Grade 3 following rechallenge with investigational medicinal product and is considered related to investigational medicinal product, then investigational medicinal product should be permanently discontinued and the subject managed according to local practice. Recurrence of laboratory abnormalities considered unrelated to investigational medicinal product may not require permanent discontinuation.

7.5.3. Grade 4 Laboratory Abnormality or Clinical Event

- For a Grade 4 clinical event or clinically significant Grade 4 laboratory abnormality confirmed by repeat testing that is considered related to investigational medicinal product, investigational medicinal product should be permanently discontinued and the subject managed according to local practice. The subject should be followed as clinically indicated until the laboratory abnormality returns to baseline or is otherwise explained, whichever occurs first. A clinically significant Grade 4 laboratory abnormality that is not confirmed by repeat testing should be managed according to the algorithm for the new toxicity grade.
- Investigational medicinal product may be continued without dose interruption for a clinically non-significant Grade 4 laboratory abnormality (eg, Grade 4 creatinine kinase (CK) after strenuous exercise or triglyceride elevation that is nonfasting or that can be medically managed) or a clinical event considered unrelated to investigational medicinal product

7.5.4. Management of Potential Nephrotoxicity

Estimated glomerular filtration rate, according to the Cockcroft-Gault ($eGFR_{CG}$) formula for creatinine clearance, will be followed post-baseline during the study. All subjects with estimated $eGFR_{CG}$ < 30 mL/min must have serum creatinine measured again within 3 calendar days of receipt of results. At the time of this repeat serum creatinine assessment, Cystatin C will also be measured and the eGFR by CKD-EPI (cystatin C) will be calculated and compared with the baseline measurement. Any subjects who have an estimated $eGFR_{CG}$ < 30 mL/min that also experience > 20% reduction in eGFR by CKD-EPI (cystatin C) from baseline or who have other clinical and/or laboratory evidence of acute renal failure will be discussed with the Medical Monitor and may discontinue from study drugs.

For subjects with $eGFR_{CG}$ < 30 mL/min who are not discontinued based on toxicity management procedures above and considered to have stable renal function per Principal Investigator and Medical Monitor, it is not mandatory to repeat eGFR assessments within 3 days.

CKD-EPI (cystatin C) formula adjusted for age and sex:

$$\text{eGFR (mL/min/1.73 m}^2\text{)} = 133 \times \min(\text{Scys}/0.8, 1)^{-0.499} \times \max(\text{Scys}/0.8, 1)^{1.328} \times 0.996^{\text{Age}} \; [\times 0.932 \text{ if female}],$$

where Scys is serum cystatin C (mg/L), min (Scys/0.8,1) indicates the minimum of Scys/κ or 1, and max (Scys/0.8,1) indicates the maximum of Scys/κ or 1.

All subjects with a change from baseline serum creatinine of ≥ 0.4 mg/dL must have serum creatinine repeated, with a concurrent urinalysis and urine chemistry, within two weeks of receipt of results. If a subject has a confirmed change from baseline serum creatinine of ≥ 0.4 mg/dL, the Medical Monitor should be notified and a consultation with a nephrologist should be obtained.

7.6. Stopping Rules for HCV Treatment

The Medical Monitor must be consulted prior to dose discontinuation of LDV/SOF unless the investigator believes that immediate action is warranted to ensure the continued safety of the subject.

Administration of study drug may be discontinued in the event of a clinical or laboratory event.

There is no option for dose reduction of LDV/SOF. If LDV/SOF FDC is stopped due to toxicity, it must not be restarted. The ESDD visit should be completed after discontinuation of LDV/SOF. The Post-HCV Treatment Week 4 and Week 12 visits must also be completed.

Subjects who meet any of the following laboratory criteria must stop HCV study treatment:

- Elevation of ALT and/or AST > 5x Day 1 or nadir, confirmed by immediate repeat testing
- Abnormal elevation of ALT > 3x Day 1 and total bilirubin > 2 x ULN, confirmed by immediate repeat testing
- Elevation of ALT > 15 x ULN, confirmed by immediate repeat testing
- Any Grade 3 or greater rash associated with constitutional symptoms
- Any Grade 4 adverse event or laboratory abnormality assessed (and confirmed by immediate repeat testing) as related to LDV/SOF

7.7. Special Situations Reports

7.7.1. Definitions of Special Situations

Special situation reports include all reports of medication error, abuse, misuse, overdose, reports of adverse events associated with product complaints, occupational exposure with an AE, and pregnancy reports regardless of an associated AE.

Medication error is any unintentional error in the prescribing, dispensing, or administration of a medicinal product while in the control of the health care provider, subject, or consumer.

Abuse is defined as persistent or sporadic intentional excessive use of a medicinal product by a subject.

Misuse is defined as any intentional and inappropriate use of a medicinal product that is not in accordance with the protocol instructions or the local prescribing information.

An overdose is defined as an accidental or intentional administration of a quantity of a medicinal product given per administration or cumulatively which is above the maximum recommended dose as per protocol or in the product labelling (as it applies to the daily dose of the subject in question). In cases of a discrepancy in drug accountability, overdose will be established only when it is clear that the subject has taken the excess dose(s). Overdose cannot be established when the subject cannot account for the discrepancy except in cases in which the investigator has reason to suspect that the subject has taken the additional dose(s).

Product complaint associated with an adverse event is defined as complaints arising from potential deviations in the manufacture, packaging, or distribution of the medicinal product associated with an adverse event, which is any untoward medical occurrence in a study subject administered a pharmaceutical product, which does not necessarily have a causal relationship with the study drug.

Occupational exposure with an AE is defined as exposure to a medicinal product as a result of one's professional or non-professional occupation.

7.7.2. Instructions for Reporting Special Situations

7.7.2.1. Instructions for Reporting Pregnancies

The investigator should report pregnancies in female study subjects that are identified after initiation of study medication and throughout the study, including the post study drug follow-up period, to the Gilead DSPH using the pregnancy report form within 24 hours of becoming aware of the pregnancy. The same reporting obligations shall apply to female partners of male study subject (ie, partner pregnancies).

Refer to Section 7.3 and the eCRF completion guidelines for full instructions on the mechanism of pregnancy reporting.

The pregnancy itself is not considered an AE nor is an induced elective abortion to terminate a pregnancy without medical reasons.

Any premature termination of pregnancy (eg, a spontaneous abortion, an induced therapeutic abortion due to complications or other medical reasons) must be reported within 24 hours as an SAE. The underlying medical reason for this procedure should be recorded as the AE term.

A spontaneous abortion is always considered to be an SAE and will be reported as described in Sections 7.1.2 and 7.3. Furthermore, any SAE occurring as an adverse pregnancy outcome post study must be reported to Gilead DSPH.

The subject should receive appropriate monitoring and care until the conclusion of the pregnancy. The outcome (of both female subject pregnancies and male subject's partner pregnancies) should be reported to Gilead DSPH using the pregnancy outcome report form. If the end of the pregnancy occurs after the study has been completed, the outcome should be reported directly to Gilead DSPH. Gilead DSPH contact information is as follows:

GSI Drug Safety and Public Health (DSPH):	Fax:	+1 (650) 522-5477
	E-mail:	safety_fc@gilead.com

Refer to Appendix 5 for Pregnancy Precautions, Definition for Female of Childbearing Potential, and Contraceptive Requirements.

7.7.2.2. Reporting Other Special Situations

All other special situation reports must be reported on the special situations report form and forwarded to Gilead DSPH within 24 hours of the investigator becoming aware of the situation. These reports must consist of situations that involve study IMP and/or Gilead concomitant medications, but do not apply to non-Gilead concomitant medications.

Special situations involving non-Gilead concomitant medications do not need to be reported on the special situations report form; however, for special situations that result in AEs due to a non-Gilead concomitant medication, the AE should be reported on the AE form.

Any inappropriate use of concomitant medications prohibited by this protocol should not be reported as "misuse," but may be more appropriately documented as a protocol deviation.

Refer to Section 7.3 and the eCRF completion guidelines for full instructions on the mechanism of special situations reporting.

All clinical sequelae in relation to these special situation reports will be reported as AEs or SAEs at the same time using the AE eCRF and/or the SAE report form. Details of the symptoms and signs, clinical management, and outcome will be reported, when available.

8. STATISTICAL CONSIDERATIONS

8.1. Analysis Objectives and Endpoints

8.1.1. Analysis Objectives

The primary objective of this study is as follows:

- To evaluate efficacy of LDV/SOF as measured by the proportion of subjects achieving HCV RNA below the lower limit of quantification (LLOQ) 12 weeks after the last dose of LDV/SOF (SVR 12).

The secondary objectives of this study are:

- To determine the proportion of subjects achieving HCV RNA below LLOQ 4 weeks after the last dose of LDV/SOF (SVR 4);
- To evaluate maintenance of HIV-1 RNA suppression after switching to E/C/F/TAF or F/R/TAF 24 weeks from the start of the F/TAF-based regimen;
- To evaluate the safety and tolerability of switching to E/C/F/TAF or F/R/TAF from the current ARV therapy in virologically-suppressed, HIV-1/HCV co-infected subjects;
- To evaluate the safety and tolerability of 12 weeks of treatment for HCV with LDV/SOF in virologically-suppressed, HIV-1/HCV co-infected subjects who switched to E/C/F/TAF or F/R/TAF.

8.1.2. Primary Endpoint

The primary efficacy endpoint is SVR12 (HCV RNA < LLOQ 12 weeks after completion of LDV/SOF treatment).

8.1.3. Secondary Endpoint

Secondary efficacy endpoints are:

- SVR4 (HCV RNA < LLOQ 4 weeks after completion of LDV/SOF treatment).
- The proportion of subjects with HIV-1 RNA ≥ 50 copies/mL (virologic failure) 24 weeks after start of the F/TAF-based regimen using modified FDA snapshot algorithm

Note: Most often, 24 weeks of HIV therapy will coincide with the Post-HCV Treatment Week 4 visit

Secondary safety endpoint is:

- Grades 1 through 4 adverse events after switch to E/C/F/TAF or F/R/TAF throughout the study and during coadministration with LDV/SOF treatment.

8.1.4. Other Endpoints of Interest

No other endpoints of interest will be considered.

8.2. Analysis Conventions

8.2.1. Analysis Sets

8.2.1.1. Efficacy

HIV Full Analysis Set: The primary analysis set for HIV efficacy analysis will be the HIV full analysis set (HIV FAS) and is defined as all subjects randomized and who received at least one dose of F/TAF-based regimen.

HCV Full Analysis Set: The primary analysis set for HCV efficacy analysis will be the HCV full analysis set (HCV FAS) and is defined as all subjects who received at least one dose of LDV/SOF.

Subjects will be grouped according to the treatment they were randomized to.

8.2.1.2. Safety

HIV Safety Analysis Set: The primary analysis set for HIV safety analyses will be the HIV safety analysis set and is defined as all subjects randomized to the study and who received at least one dose of F/TAF-based regimen.

HCV Safety Analysis Set: The primary analysis set for HCV safety analyses with coadministration with the F/TAF-based regimen in Part 2 will be the HCV safety analysis set and is defined as all subjects who received at least one dose of LDV/SOF.

All data collected during treatment will be included in the safety summaries. Subjects will be grouped according to the treatment they actually received.

8.2.1.3. Pharmacokinetics

The PK analysis set will include all subjects who were randomized and have received at least one dose of either the F/TAF-based regimen (at Week 4) or coadministration of the F/TAF-based regimen and LDV/SOF, and for whom concentration data of any analyte of interest are available. The PK analysis set will be used for listing PK concentration data and summaries.

8.3. Demographic Data and Baseline Characteristics

Demographic and baseline measurements will be summarized by standard descriptive methods using the safety analysis set. Demographic and baseline measurements will be summarized by treatment group (E/C/F/TAF; F/R/TAF) and overall.

Summaries will include sex, race/ethnicity, age, height, weight and body-mass index.

Baseline data will include a summary of risk factors for HIV-1 infection and HCV infection, and enrollment distribution.

For categorical demographic and baseline characteristics, a Cochran-Mantel-Haenszel (CMH) test will be used to compare treatment groups. For continuous demographic and baseline characteristics, a Wilcoxon rank sum test will be used to compare the treatment groups.

8.4. Efficacy Analysis

8.4.1. Primary Analysis

The primary efficacy endpoint is SVR12 (HCV RNA < LLOQ 12 weeks after discontinuation of therapy). The primary analysis will be performed after all enrolled subjects have been followed through post-HCV treatment Week 12 or discontinued from study.

In the primary efficacy analysis, the SVR12 rate will be compared to the performance goal of 88% by using two-sided exact one-sample binomial test at the 0.05 significance level. The basis for this benchmark includes the overall trend toward increasing SVR rates in recent years; and the general appeal of using a fixed clinically relevant threshold as a measure of treatment benefit of LDV/SOF.

The null (H_0) and alternative (H_1) hypotheses used to assess superiority of LDV/SOF relative to the performance goal of 88% are:

- H_0: SVR12 = 88%
- H_1: SVR12 ≠ 88%

The primary efficacy analysis will also include the construction of an associated two-sided exact 95% CI.

Furthermore, SVR12 will also be summarized by the HIV treatment arms (F/TAF-based regimen).

8.4.2. Secondary Analyses

The methods to assess proportion of HIV-1 RNA ≥ 50 copies/mL (virologic failure) 24 weeks after start of the F/TAF-based regimen using a modified FDA snapshot algorithm will be provided in detail in the Statistical Analysis Plan.

The changes from baseline in CD4+ count at Week 8 and Post-HCV Treatment Week 12 will be summarized using descriptive statistics.

The analysis of SVR4 will be summarized using similar methods as for the primary endpoint.

8.5. Safety Analysis

All safety data collected on or after the date F/TAF-based ARV regimen was first dispensed up to the date of last dose of study drug(s) (either F/TAF-based regimen alone or in combination with LDV/SOF) plus 30 days will be summarized overall, according to the study drug(s) received.

Additionally, safety data collection on or after the first dose of LDV/SOF until the last dose of LDV/SOF plus 30 days (ie, during coadministration of HIV/HCV study drugs), will also be summarized.

Data for pretreatment and post-last-dose-plus-30-days will be included in data listings.

8.5.1. Extent of Exposure

A subject's extent of exposure to each study drug will be generated from the corresponding study drug administration data. Duration of exposure to each study drug will be expressed as the number of weeks between the first and last dose for each study drug, inclusive, regardless of temporary interruptions in study drug administration.

Dosing information for individual subjects will be listed.

8.5.2. Adverse Events

Clinical and laboratory adverse events (AEs) will be coded using the Medical Dictionary for Regulatory Activities (MedDRA). System Organ Class (SOC), High-Level Group Term (HLGT), High-Level Term (HLT), Preferred Term (PT), and Lower-Level Term (LLT) will be attached to the clinical database.

HIV treatment-emergent AEs are events that meet one of the following criteria up to 30 days after the permanent discontinuation of HIV study drug(s):

- AEs with onset dates on or after the first dose date of HIV study drug(s) and no later than 30 days after permanent discontinuation of HIV study drug(s), or,
- AEs that result in permanent study drug discontinuation of HIV study drug(s).

The number and percentage of subjects with treatment-emergent AEs (by SOC and PT) will be summarized as described in Section 8.5. A similar definition will be used to define HCV treatment-emergent AEs during coadministration of HIV/HCV study drugs. Additional summaries will include summaries for AEs by grade, Investigator's assessment of relationship to study drug, and effect on study drug dosing.

All treatment-emergent and non-treatment emergent AEs will be included in a data listing.

8.5.3. Laboratory Evaluations

Selected laboratory data will be summarized using only observed data. Absolute values and changes from baseline at all scheduled time points will be summarized.

Graded laboratory abnormalities will be defined using the GSI Grading Scale for severity of Adverse Events and Laboratory Abnormalities (Appendix 4).

Incidence of treatment-emergent laboratory abnormalities, defined as values that increase at least one toxicity grade from baseline at any time post baseline up to the date of last dose of HIV study drug(s) plus 30 days, will be summarized as described in Section 8.5. Similarly, treatment-emergent abnormalities during coadministration of HIV/HCV study drugs will also be summarized. If baseline data are missing, then any graded abnormality (ie, at least a Grade 1) will be considered treatment emergent. The maximum toxicity grade will be summarized by laboratory parameter.

Laboratory abnormalities that occur before the first dose of HIV study drug(s) or after the subject has been discontinued from HIV study drug(s) plus 30 days will be included in a data listing.

8.5.4. Other Safety Evaluations

Weight will be summarized by visit.

Safety ECGs will be listed and summarized for subjects in the Safety Analysis Set. The number and percent of subjects with abnormal safety ECG will be summarized by visit.

8.6. Pharmacokinetic Analysis

Population PK assessments may be conducted for some of the relevant analytes: TAF, TFV, RPV, FTC, EVG, COBI, LDV, SOF, or GS-331007. Population PK assessments will be summarized in a separate report.

8.7. Biomarker Analysis

The renal, vascular and inflammatory markers (see Sections 6.8.2 – 6.8.4 for details) will be summarized by treatment group and visit using descriptive statistics. The difference in change from baseline in these biomarkers between the treatment groups (E/C/F/TAF and F/R/TAF) will be tested using Wilcoxon rank sum test.

8.8. Sample Size

A sample size of 240 subjects will provide at least 85% power to detect an improvement of at least 6 percentage points in SVR12 rate from the performance goal of 88% by using a two-sided exact one-sample binomial test at significance level of 0.05.

9. RESPONSIBILITIES

9.1. Investigator Responsibilities

9.1.1. Good Clinical Practice

The investigator will ensure that this study is conducted in accordance with the principles of the Declaration of Helsinki (as amended in Edinburgh, Tokyo, Venice, Hong Kong, and South Africa), International Conference on Harmonisation (ICH) guidelines, or with the laws and regulations of the country in which the research is conducted, whichever affords the greater protection to the study subject. These standards are consistent with the European Union Clinical Trials Directive 2001/20/EC and Good Clinical Practice Directive 2005/28/EC.

The investigator will ensure adherence to the basic principles of Good Clinical Practice, as outlined in 21 CFR 312, subpart D, “Responsibilities of Sponsors and Investigators,” 21 CFR 50, “Protection of Human Subjects,” and 21 CFR 56, ”Institutional Review Boards”.

The investigator and all applicable sub-investigators will comply with 21 CFR 54, ”Financial Disclosure by Clinical Investigators”, providing documentation of their financial interest or arrangements with Gilead, or proprietary interests in the investigational drug under study. This documentation must be provided prior to the investigator’s (and any sub-investigator’s) participation in the study. The investigator and sub-investigator agree to notify Gilead of any change in reportable interests during the study and for 1 year following completion of the study. Study completion is defined as the date when the last subject completes the protocol-defined activities.

9.1.2. Institutional Review Board (IRB)/Independent Ethics Committee (IEC) Review and Approval

The investigator (or sponsor as appropriate according to local regulations) will submit this protocol, informed consent form, and any accompanying material to be provided to the subject (such as advertisements, subject information sheets, or descriptions of the study used to obtain informed consent) to an IRB/IEC. The investigator will not begin any study subject activities until approval from the IRB/IEC has been documented and provided as a letter to the investigator.

Before implementation, the investigator will submit to and receive documented approval from the IRB/IEC any modifications made to the protocol or any accompanying material to be provided to the subject after initial IRB/IEC approval, with the exception of those necessary to reduce immediate risk to study subjects.

9.1.3. Informed Consent

The investigator is responsible for obtaining written informed consent from each individual participating in this study after adequate explanation of the aims, methods, objectives, and potential hazards of the study and before undertaking any study-related procedures. The

investigator must use the most current IRB- or IEC-approved consent form for documenting written informed consent. Each informed consent (or assent as applicable) will be appropriately signed and dated by the subject or the subject's legally authorized representative and the person conducting the consent discussion, and also by an impartial witness if required by IRB/IEC or local requirements.

9.1.4. Confidentiality

The Investigator must assure that subjects' anonymity will be strictly maintained and that their identities are protected from unauthorized parties. Only subject initials, date of birth, another unique identifier (as allowed by local law) and an identification code will be recorded on any form or biological sample submitted to the Sponsor, or laboratory. Laboratory specimens must be labeled in such a way as to protect subject identity while allowing the results to be recorded to the proper subject. Refer to specific laboratory instructions NOTE: The Investigator must keep a screening log showing codes, names, and addresses for all subjects screened and for all subjects enrolled in the trial. Subject data will be processed in accordance with all applicable regulations.

The investigator agrees that all information received from Gilead, including but not limited to the investigator brochure, this protocol, eCRF, the IMP, and any other study information, remain the sole and exclusive property of Gilead during the conduct of the study and thereafter. This information is not to be disclosed to any third party (except employees or agents directly involved in the conduct of the study or as required by law) without prior written consent from Gilead. The investigator further agrees to take all reasonable precautions to prevent the disclosure by any employee or agent of the study site to any third party or otherwise into the public domain.

9.1.5. Study Files and Retention of Records

The investigator must maintain adequate and accurate records to enable the conduct of the study to be fully documented and the study data to be subsequently verified. These documents should be classified into at least the following two categories: (1) investigator's study file, and (2) subject clinical source documents.

The investigator's study file will contain the protocol/amendments, eCRF and query forms, IRB/IEC and governmental approval with correspondence, informed consent, drug records, staff curriculum vitae and authorization forms, and other appropriate documents and correspondence.

The required source data should include sequential notes containing at least the following information for each subject:

- Subject identification (name, date of birth, gender);
- Documentation that subject meets eligibility criteria, ie, history, physical examination, and confirmation of diagnosis (to support inclusion and exclusion criteria);
- Documentation of the reason(s) a consented subject is not enrolled
- Participation in study (including study number);

- Study discussed and date of informed consent;
- Dates of all visits;
- Documentation that protocol specific procedures were performed;
- Results of efficacy parameters, as required by the protocol;
- Start and end date (including dose regimen) of IMP, including dates of dispensing and return;
- Record of all adverse events and other safety parameters (start and end date, and including causality and severity);
- Concomitant medication (including start and end date, dose if relevant; dose changes);
- Date of study completion and reason for early discontinuation, if it occurs.

All clinical study documents must be retained by the investigator until at least 2 years or according to local laws, whichever is longer, after the last approval of a marketing application in an ICH region (ie, United States, Europe, or Japan) and until there are no pending or planned marketing applications in an ICH region; or, if no application is filed or if the application is not approved for such indication, until 2 years after the investigation is discontinued and regulatory authorities have been notified. Investigators may be required to retain documents longer if specified by regulatory requirements, by local regulations, or by an agreement with Gilead. The investigator must notify Gilead before destroying any clinical study records.

Should the investigator wish to assign the study records to another party or move them to another location, Gilead must be notified in advance.

If the investigator cannot provide for this archiving requirement at the study site for any or all of the documents, special arrangements must be made between the investigator and Gilead to store these records securely away from the site so that they can be returned sealed to the investigator in case of an inspection. When source documents are required for the continued care of the subject, appropriate copies should be made for storage away from the site.

9.1.6. Case Report Forms

For each subject consented, an eCRF will be completed by an authorized study staff member whose training for this function is documented according to study procedures. eCRF should be completed on the day of the subject visit to enable the sponsor to perform central monitoring of safety data. The Eligibility Criteria eCRF should be completed only after all data related to eligibility have been received. Subsequent to data entry, a study monitor will perform source data verification within the electronic data capture (EDC) system. Original entries as well as any changes to data fields will be stored in the audit trail of the system. Prior to database lock (or any interim time points as described in the clinical data management plan), the investigator will use his/her log in credentials to confirm that the forms have been reviewed, and that the entries

accurately reflect the information in the source documents. The eCRF capture the data required per the protocol schedule of events and procedures. System-generated or manual queries will be issued to the investigative site staff as data discrepancies are identified by the monitor or internal Gilead staff, who routinely review the data for completeness, correctness, and consistency. The site coordinator is responsible for responding to the queries in a timely manner, within the system, either by confirming the data as correct or updating the original entry, and providing the reason for the update (eg, data entry error). At the conclusion of the trial, Gilead will provide the site with a read-only archive copy of the data entered by that site. This archive must be stored in accordance with the records retention requirements outlined in Section 9.1.5.

9.1.7. Investigational Medicinal Product Accountability and Return

Gilead recommends that used and unused IMP supplies be returned to the shipping facility from which it came for eventual destruction. The study monitor will provide instructions for return. If return is not possible, the study monitor will evaluate each study center's IMP disposal procedures and provide appropriate instruction for destruction of unused IMP supplies. If the site has an appropriate standard operating procedure (SOP) for drug destruction as determined by Gilead Quality Assurance (QA), the site may destroy used (empty or partially empty) and unused IMP supplies in accordance with that site's approved SOP. A copy of the site's approved SOP will be obtained for central files.

If IMP is destroyed on site, the investigator must maintain accurate records for all IMP destroyed. Records must show the identification and quantity of each unit destroyed, the method of destruction, and the person who disposed of the IMP. Upon study completion, copies of the IMP accountability records must be filed at the site. Another copy will be returned to Gilead.

The study monitor will review IMP supplies and associated records at periodic intervals.

9.1.8. Inspections

The investigator will make available all source documents and other records for this trial to Gilead's appointed study monitors, to IRB/IEC, or to regulatory authority or health authority inspectors.

9.1.9. Protocol Compliance

The investigator is responsible for ensuring the study is conducted in accordance with the procedures and evaluations described in this protocol.

9.2. Sponsor Responsibilities

9.2.1. Protocol Modifications

Protocol modifications, except those intended to reduce immediate risk to study subjects, may be made only by Gilead. The investigator must submit all protocol modifications to the IRB/IEC in accordance with local requirements and receive documented IRB/IEC approval before modifications can be implemented.

9.2.2. Study Report and Publications

A clinical study report (CSR) will be prepared and provided to the regulatory agencies. Gilead will ensure that the report meets the standards set out in the ICH Guideline for Structure and Content of Clinical Study Reports (ICH E3). Note that an abbreviated report may be prepared in certain cases.

Investigators in this study may communicate, orally present, or publish in scientific journals or other scholarly media only after the following conditions have been met: the results of the study in their entirety have been publicly disclosed by or with the consent of Gilead in an abstract, manuscript, or presentation form or the study has been completed at all study sites for at least 2 years.

The investigator will submit to Gilead any proposed publication or presentation along with the respective scientific journal or presentation forum at least 30 days before submission of the publication or presentation.

No such communication, presentation, or publication will include Gilead's confidential information (see Section 9.1.4).

The investigator will comply with Gilead's request to delete references to its confidential information (other than the study results) in any paper or presentation and agrees to withhold publication or presentation for an additional 60 days in order to obtain patent protection if deemed necessary.

9.3. Joint Investigator/Sponsor Responsibilities

9.3.1. Payment Reporting

Investigators and their study staff may be asked to provide services performed under this protocol, e.g. attendance at Investigator's Meetings. If required under the applicable statutory and regulatory requirements, Gilead will capture and disclose to Federal and State agencies any expenses paid or reimbursed for such services, including any clinical trial payments, meal, travel expenses or reimbursements, consulting fees, and any other transfer of value.

9.3.2. Access to Information for Monitoring

In accordance with regulations and guidelines, the study monitor must have direct access to the investigator's source documentation in order to verify the accuracy of the data recorded in the CRF/eCRF.

The monitor is responsible for routine review of the CRF/eCRF at regular intervals throughout the study to verify adherence to the protocol and the completeness, consistency, and accuracy of the data being entered on them. The monitor should have access to any subject records needed to verify the entries on the CRF/eCRF. The investigator agrees to cooperate with the monitor to ensure that any problems detected through any type of monitoring (central, on site) are resolved.

9.3.3. Access to Information for Auditing or Inspections

Representatives of regulatory authorities or of Gilead may conduct inspections or audits of the clinical study. If the investigator is notified of an inspection by a regulatory authority the investigator agrees to notify the Gilead medical monitor immediately. The investigator agrees to provide to representatives of a regulatory agency or Gilead access to records, facilities, and personnel for the effective conduct of any inspection or audit.

9.3.4. Study Discontinuation

Both the sponsor and the investigator reserve the right to terminate the study at any time. Should this be necessary, both parties will arrange discontinuation procedures and notify the appropriate regulatory authority(ies), IRBs, and IECs. In terminating the study, Gilead and the investigator will assure that adequate consideration is given to the protection of the subjects' interests.

10. REFERENCES

Backus LI, Boothroyd DB, Phillips BR, Belperio P, Halloran J, Mole LA. A sustained virologic response reduces risk of all-cause mortality in patients with hepatitis C. Clin Gastroenterol Hepatol 2011;9 (6):509-16 e1.

Bhagani S, Sulkowski MS, Cooper C, Kwo PY, Kowdley K, Mertens M, et al. Ledipasvir/Sofosbuvir is Safe and Effective for the Treatment of Patients with Genotype 1 Chronic HCV Infection in Both HCV Mono-and HCV/HIV Co-infected Patients [Abstract: 386]. European AIDS Clinical Society (EACS); 2015 21-24 October; Barcelona, Spain.

Campsmith ML, Rhodes PH, Hall HI, Green TA. Undiagnosed HIV prevalence among adults and adolescents in the United States at the end of 2006. J Acquir Immune Defic Syndr 2010;53 (5):619-24.

Carrat F, Bani-Sadr F, Pol S, Rosenthal E, Lunel-Fabiani F, Benzekri A, et al. Pegylated interferon alfa-2b vs standard interferon alfa-2b, plus ribavirin, for chronic hepatitis C in HIV-infected patients: a randomized controlled trial. JAMA 2004;292 (23):2839-48.

Chesney M. Factors affecting adherence to antiretroviral therapy. Clin Infect Dis. 2000;30 (Suppl 2):S-171-S6.

Cockcroft DW, Gault MH. Prediction of creatinine clearance from serum creatinine. Nephron 1976;16:31-41.

Custodio J, Doyle E, Pang PS, Das M, H. C, Ma G, et al. Lack of Drug Interactions between Boosted and Unboosted Tenofovir Alafenamide-based Antiretroviral Single Tablet Regimens (Emtricitabine/Rilpivirine/Tenofovir Alafenamide and Elvitegravir/Cobicistat/Emtricitabine/Tenofovir Alafenamide) and the anti-HCV Single Tablet Regimen Ledipasvir/Sofosbuvir [Abstract 727]. Infectious Diseases Society of America (IDSA); 2015 7-11 October; San Diego, CA.

Deng LP, Gui XE, Zhang YX, Gao SC, Yang RR. Impact of human immunodeficiency virus infection on the course of hepatitis C virus infection: a meta-analysis. World J Gastroenterol 2009;15 (8):996-1003.

Denniston MM, Jiles RB, Drobeniuc J, Klevens RM, Ward JW, McQuillan GM, et al. Chronic Hepatitis C Virus Infection in the United States, National Health and Nutrition Examination Survey 2003 to 2010. Ann Intern Med 2014;160:293-300.

Department for Health and Human Services (DHHS). Guidelines for the Use of Antiretroviral Agents in HIV-1-Infected Adults and Adolescents. Developed by the DHHS Panel on Antiretroviral Guidelines for Adults and Adolescents – A Working Group of the Office of AIDS Research Advisory Council (OARAC). Downloaded from http://aidsinfo.nih.gov/guidelines on 5/11/2015. Last Updated 08 April, 2015.

Gay CL, Mayo AJ, Mfalila CK, Chu H, Barry AC, Kuruc JD, et al. Efficacy of NNRTI-based antiretroviral therapy initiated during acute HIV infection. AIDS 2011;25 (7):941-9.

Gilead Sciences Canada Inc. SOVALDI® (Sofosbuvir) Tablets 400 mg. Product Monograph. Mississauga, ON. December 2013:

Gilead Sciences Inc. SOVALDI® (sofosbuvir) tablets, for oral use. US Prescribing Information. Foster City, CA. December 2013:

Gilead Sciences Inc. HARVONI® (ledipasvir and sofosbuvir) tablets, for oral use. US Prescribing Information. Foster City, CA. Revised November 2015:

Gilead Sciences Inc. HARVONI® (ledipasvir and sofosbuvir) tablets, for oral use. US Prescribing Information. Foster City, CA October 2014:

Gilead Sciences Limited. SOVALDI 400 mg film-coated tablets. Summary of Product Characteristics. County Cork Ireland. January 2014. 2014:

Graham CS, Baden LR, Yu E, Mrus JM, Carnie J, Heeren T, et al. Influence of human immunodeficiency virus infection on the course of hepatitis C virus infection: a meta-analysis. Clin Infect Dis 2001;33 (4):562-9.

HCV Guidelines. Recommendations for Testing, Managing, and Treating Hepatitis C. 2015.

Laguno M, Murillas J, Blanco JL, Martinez E, Miquel R, Sanchez-Tapias JM, et al. Peginterferon alfa-2b plus ribavirin compared with interferon alfa-2b plus ribavirin for treatment of HIV/HCV co-infected patients. AIDS 2004;18 (13):F27-36.

Lavanchy D. Evolving epidemiology of hepatitis C virus. Clin Microbiol Infect 2011;17 (2):107-15.

Ly KN, Xing J, Klevens RM, Jiles RB, Ward JW, Holmberg SD. The increasing burden of mortality from viral hepatitis in the United States between 1999 and 2007. Ann Intern Med 2012;156 (4):271-8.

Mahajan R, Xing J, Liu SJ, Ly KN, Moorman AC, Rupp L, et al. Mortality Among Persons in Care With Hepatitis C Virus Infection: The Chronic Hepatitis Cohort Study (CHeCS), 2006-2010. Clin Infect Dis 2014.

Mills A, Arribas JR, Andrade-Villanueva J, DiPerri G, Van Lunzen J, Koenig E, et al. Switching from tenofovir disoproxil fumarate to tenofovir alafenamide in antiretroviral regimens for virologically suppressed adults with HIV-1 infection: a randomised, active-controlled, multicentre, open-label, phase 3, non-inferiority study [Supplementary Webappendix]. Lancet 2015.

Mocroft A, Vella S, Benfield TL, Chiesi A, Miller V, Gargalianos P, et al. Changing patterns of mortality across Europe in patients infected with HIV-1. EuroSIDA Study Group. Lancet 1998;352 (9142):1725-30.

Myers RP, Liu M, Shaheen AA. The burden of hepatitis C virus infection is growing: a Canadian population-based study of hospitalizations from 1994 to 2004. Can J Gastroenterol 2008;22 (4):381-7.

Naggie S, Cooper C, Saag M, Workowski K, Ruane P, Towner WJ, et al. Ledipasvir and Sofosbuvir for HCV in Patients Coinfected with HIV-1. N Engl J Med 2015.

Nakano T, Lau GM, Sugiyama M, Mizokami M. An updated analysis of hepatitis C virus genotypes and subtypes based on the complete coding region. Liver Int 2012;32 (2):339-45.

Palella FJ, Delaney KM, Moorman AC, Loveless MO, Fuhrer J, Satten GA, et al. Declining Morbidity and Mortality Among Patients With Advanced Human Immunodeficiency Virus Infection. N Eng J Med 1998;338 (13):853-60.

Panel on Antiretroviral Guidelines for Adults and Adolescents. Guidelines for the use of antiretroviral agents in HIV-1-infected adults and adolescents. Department of Health and Human Services. 1–239. Available at http://www.aidsinfo.nih.gov/ContentFiles/AdultandAdolescentGL.pdf. Accessed August 22. 2012.

Salmon-Ceron D, Lewden C, Morlat P, Bevilacqua S, Jougla E, Bonnet F, et al. Liver disease as a major cause of death among HIV infected patients: role of hepatitis C and B viruses and alcohol. J Hepatol 2005;42 (6):799-805.

Sax PE, Wohl D, Yin MT, Post F, DeJesus E, Saag M, et al. Tenofovir alafenamide versus tenofovir disoproxil fumarate, coformulated with elvitegravir, cobicistat, and emtricitabine, for initial treatment of HIV-1 infection: two randomised, double-blind, phase 3, non-inferiority trials. Lancet 2015;385:2606-15.

Selik RM, Mokotoff ED, Branson B, Owen SM, Whitmore S, Hall HI. Morbidity and Mortality Weekly Report (MMWR). Revised Surveillance Case Definition for HIV Infection - United States 2014. Centers for Disease Control and Prevention, 2014.

Smith BD, Morgan ML, Beckett GA, Falck-Ytter Y, Holtzman D, Teo CG, et al. Recommendations for the Identification of Chronic Hepatitis C Virus Infection Among Persons Born During 1945–1965. Centers for Disease Control and Prevention MMWR August, 2012.

Staples CT, Jr., Rimland D, Dudas D. Hepatitis C in the HIV (human immunodeficiency virus) Atlanta V.A. (Veterans Affairs Medical Center) Cohort Study (HAVACS): the effect of coinfection on survival. Clin Infect Dis 1999;29 (1):150-4.

Sterne J, Hernán M, Ledergerber B, Tilling K, Weber R, Sendi P, et al. Long-term effectiveness of potent antiretroviral therapy in preventing AIDS and death: a prospective cohort study. Lancet 2005;366 (9483):378-84.

Stone VE JJ, Tolson J, Pilon T. Potential impact of once daily regimens on adherence to HAART [abstract]. . 40th Annual Meeting of the Infectious Disease Society of America; 2002 Oct 24-27; Chicago. pp. p.129, Abstract 486.

The Joint United Nations Programme on HIV/AIDS (UNAIDS). Regional Fact Sheet 2012. North America, Western and Central Europe. 2012.

Thomas DL. The challenge of hepatitis C in the HIV-infected person. Annu Rev Med 2008;59:473-85.

Torriani FJ, Rodriguez-Torres M, Rockstroh JK, Lissen E, Gonzalez-Garcia J, Lazzarin A, et al. Peginterferon Alfa-2a plus ribavirin for chronic hepatitis C virus infection in HIV-infected patients. N Engl J Med 2004;351 (5):438-50.

World Health Organization (WHO). World Health Organization, UNAIDS, UNICEF. Global HIV/AIDS Response: Epidemic update and health sector progress towards universal access. Progress Report 2011. 2011.

11. APPENDICES

Appendix 1. Investigator Signature Page
Appendix 2. Study Procedures Table
Appendix 3. Management of Clinical and Laboratory Adverse Events (HIV ARV Agents only)
Appendix 4. GSI Grading Scale for Severity of Adverse Events and Laboratory Abnormalities
Appendix 5. Pregnancy Precautions, Definition for Female of Childbearing Potential, and Contraceptive Requirements
Appendix 6. CDC Classification Systems, AIDS-Indicator Conditions

Appendix 1. Investigator Signature Page

GILEAD SCIENCES, INC.
333 LAKESIDE DRIVE
FOSTER CITY, CA 94404

STUDY ACKNOWLEDGEMENT

A Phase 3b Randomized, Open-label, Controlled Study of the Efficacy, Safety and Tolerability of 12 Weeks of Ledipasvir/Sofosbuvir (LDV/SOF) Treatment for HIV/HCV Co-infected Subjects who Switch to Elvitegravir/Cobicistat/Emtricitabine/Tenofovir Alafenamide (E/C/F/TAF) or Emtricitabine/Rilpivirine/Tenofovir Alafenamide (F/R/TAF) prior to LDV/SOF HCV Treatment, the HIV/HCV Co-STARs study (Co-infection treatment with Single Tablet Antiviral Regimens)

GS-US-366-1992 Amendment 1, 27 May 2016

This protocol has been approved by Gilead Sciences, Inc. The following signature documents this approval.

Richard Haubrich

Richard Haubrich (Printed)
Medical Monitor

PPD

Signature

1 June 2016

Date

INVESTIGATOR STATEMENT

I have read the protocol, including all appendices, and I agree that it contains all necessary details for me and my staff to conduct this study as described. I will conduct this study as outlined herein and will make a reasonable effort to complete the study within the time designated.

I will provide all study personnel under my supervision copies of the protocol and access to all information provided by Gilead Sciences, Inc. I will discuss this material with them to ensure that they are fully informed about the drugs and the study.

Principal Investigator Name (Printed)

Signature

Date

Site Number

Appendix 2. Study Procedures Table

			End of Week[c] Part 1 On Treatment		End of Week[c] – Part 2							
					On Treatment				Post-HCV Treatment			
	Screening[a]	Day 1[b]	4	6	8	12	16	20	4	12	30-Day Follow-Up[d]	ESDD[e]
Written Informed Consent	X											
Medical History	X											
Liver Cirrhosis Determination	X											
Concomitant Medications	X	X	X		X	X	X	X	X	X	X	X
Adverse Events	X	X	X		X	X	X	X	X	X	X	X
Complete Physical Examination	X	X								X		X
Symptom-Directed Physical Examination[f]			X		X	X	X	X	X		X	
Vital Signs & Weight	X	X	X		X	X	X	X	X	X	X	X
Height	X											
12-Lead ECG (performed supine)	X											X
Urinalysis & Urine Chemistry	X	X	X		X	X	X	X	X	X	X	X
Urine Storage Sample		X	X		X	X	X	X	X	X		X
Urine Pregnancy Test[g]		X	X		X	X	X	X	X	X	X	X
Serum Pregnancy Test[g]	X											
Chemistry Profile[h]	X	X	X		X	X	X	X	X	X	X	X
Hematology Profile[i]	X	X	X		X	X	X	X	X	X	X	X
Coagulation Assessments[j]	X	X	X		X	X	X	X	X	X		
Metabolic Assessments[k]		X			X			X		X		
CD4+ Cell Count	X	X	X		X	X	X	X	X	X	X	X

			End of Week[c] Part 1 On Treatment		End of Week[c] – Part 2							
					On Treatment				Post-HCV Treatment			
	**Screening[a]**	**Day 1[b]**	**4**	**6**	**8**	**12**	**16**	**20**	**4**	**12**	**30-Day Follow-Up[d]**	**ESDD[e]**
Plasma HIV-1 RNA	X	X	X	X	X	X	X	X	X	X	X	X
HIV-1 Proviral Genotype[l]	X											
HIV-1 Genotype/Phenotype[m]												X
Plasma HCV RNA	X	X			X	X	X	X	X	X	X	X
HCV Genotype & Subtype	X											
HCV IL28B Genotype	X											
HIV, HBV & HCV Serologies[n]	X											
Evaluations of Bone & Renal Safety, Inflammation and Platelet and Coagulation Function[o]		X	X		X	X		X		X		X
Plasma Storage Sample[p]		X	X	X	X	X	X	X	X	X		X
Serum Storage Sample[p]		X	X		X	X	X	X	X	X		X
PPD		X	X		X			X		X		X
Estimated GFR_{CG}[r]	X	X	X		X	X	X	X	X	X		X
Population PK[s]			X		X	X	X	X				
Health Related Questionnaires[t]		X			X			X		X		X
Randomization		X										
Study Drug Dispensation and Accountability[u]		X	X		X	X	X	X	X	X		X[v]

a Evaluations to be completed within 42 days prior to the Day 1 visit
b Subjects will be dispensed HIV study drugs only at the Day 1 visit; initiation of HIV treatment with the study drugs must take place within 24 hours after the Day 1 visit.
c All study visits are to be scheduled relative to the Day 1 visit date. Visit windows are ± 2 days of the protocol specified date through Post-HCV Treatment Week 4 visit, and ± 4 days of the protocol specified date at Post-HCV Treatment Week 12.

d For the purpose of scheduling a 30-Day Follow-Up Visit, a ± 6 days window may be used

e Early Study Drug Discontinuation visit to occur within 72 hours of last dose of study drug. Refer to Section 6.5.1 for management of early study drug discontinuation of Parts 1 and 2.

f Symptom-directed physical examination as needed

g For females of childbearing potential only as defined by Appendix 5. Positive urine pregnancy tests will be confirmed with a serum test.

h Chemistry profile: alkaline phosphatase, AST, ALT, total bilirubin, direct and indirect bilirubin, total protein, albumin, bicarbonate, BUN, calcium, chloride, creatinine, glucose, phosphorus, magnesium, potassium, sodium, and uric acid. At visits in which metabolic assessments are to be performed, analyses of glucose will be done as part of the fasting metabolic assessments and not as part of the chemistry profile

i CBC with differential and platelet count

j Coagulation assessments: International Normalized Ratio (INR), prothrombin time (PT), and activated partial thromboplastin time (APTT)

k Metabolic assessments (collected fasted, no food or drink, except water, at least 8 hours prior to blood collection): glucose and lipid panel (total cholesterol, HDL, direct LDL, and triglycerides)

l If a historical genotype report prior to first ARV is not available, or subject has 3 or more prior ARV regimens, whole blood sample for proviral genotype analysis of archived resistance

m HIV-1 genotype/phenotype resistance testing for subjects with unconfirmed virologic rebound with HIV-1 RNA value ≥ 400 copies/mL

n Serology testing includes HIV and HCV antibody, and HBV core antibody (HBcAb), surface antibody (HBsAb), surface antigen (HBsAg), e-antibody (HBeAg), e-antibody (HBeAb), and HBV DNA.

o Blood for bone safety, parathyroid (PTH) and serum OH-25 vitamin D; Inflammation may include cystatin-C, IL-6, hs-CRP, sCD14, sCD163, sTNF-1R, and Lp-PLA2; Platelet and coagulation function may include soluble glycoprotein VI (sGPVI), P-selectin, soluble CD40 ligand, and D-dimer will be collected. Urine for renal safety, including retinol binding protein, beta-2-microglobulin, urine albumin, and urine protein will be collected. Samples will be collected fasted. If the subject has not fasted prior to the visit, the visit may proceed, but the subject must return within 72 hours in a fasted state.

p Plasma storage samples for safety, virology, and/or PK testing. Serum storage samples for possible additional clinical testing.

q PPD

r Estimated GFR according to the Cockcroft-Gault formula for creatinine clearance (see Section 4.2 for details)

s Two (2) blood samples will be collected: 1) the predose sample should be drawn prior to observed study drug dosing; 2) the postdose sample should be drawn between 15 mins and 4 hours postdose

t Health related questionnaires: adherence for HIV (VAS), HIVTSQs – Status version (at Day 1 only), HIVTSQc – Change version (at all other visits), SF-36, FACIT-F, CLDQ-HCV, and WPAI: Hepatitis C.

u Part 1: HIV study drugs are dispensed after all Day 1 assessments are completed and randomization occurred, and through the duration of the study. Part 2: After determination of HIV suppression (< 50 copies/mL) based on the Week 6 HIV-1 RNA value and tolerability of HIV study drugs, LDV/SOF will be dispensed at Weeks 8, 12, and 16 only. Post-HCV Treatment Week 12: drug accountability only; study drug will not be dispensed at this visit.

v If subjects discontinue during Part 1, only drug accountability will be performed; study drug will not be dispensed at this visit. In Part 2, if subject discontinues LDV/SOF but still continues with HIV study drug, HIV study drug will be dispensed until the last study visit. If subject discontinues HIV study drug but still continues with LDV/SOF, LDV/SOF will be dispensed. If subject discontinues both HIV and HCV study drugs, only drug accountability will be performed; no dispensation will occur at this visit.

Appendix 3. Management of Clinical and Laboratory Adverse Events (HIV ARV Agents only)

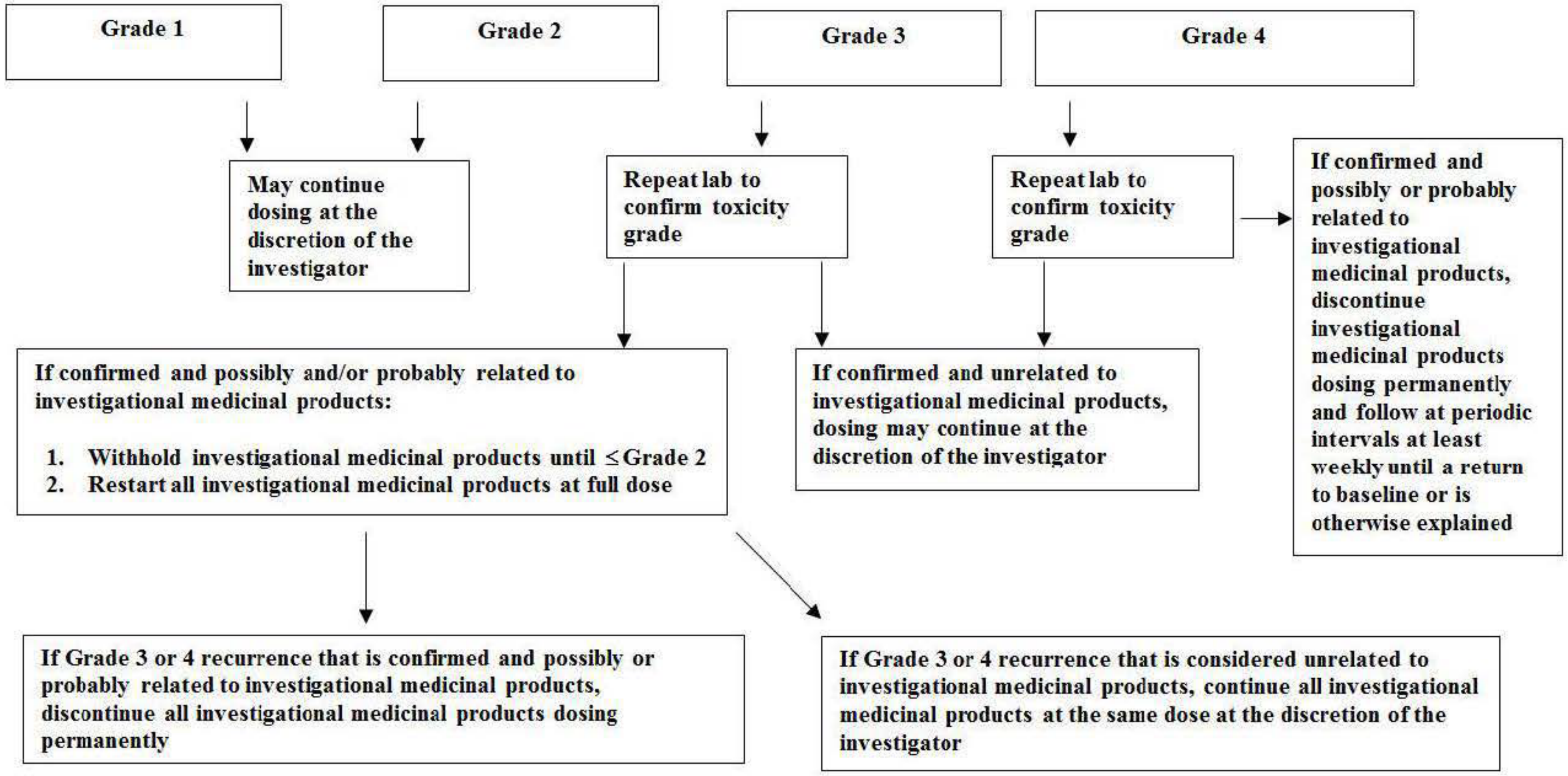

Appendix 4. GSI Grading Scale for Severity of Adverse Events and Laboratory Abnormalities

Antiviral Toxicity Grading Scale Version: 01 April 2015

HEMATOLOGY				
	**Grade 1**	**Grade 2**	**Grade 3**	**Grade 4**
Hemoglobin<br>HIV POSITIVE<br>**Adult and Pediatric ≥ 57 Days**	8.5 to 10.0 g/dL<br>85 to 100 g/L	7.5 to < 8.5 g/dL<br>75 to < 85 g/L	6.5 to < 7.5 g/dL<br>65 to < 75 g/L	< 6.5 g/dL<br>< 65 g/L
HIV NEGATIVE<br>**Adult and Pediatric ≥ 57 Days**	10.0 to 10.9 g/dL<br>100 to 109 g/L<br>OR<br>Any decrease from Baseline<br>2.5 to < 3.5 g/dL<br>25 to < 35 g/L	9.0 to < 10.0 g/dL<br>90 to < 100 g/L<br>OR<br>Any decrease from Baseline<br>3.5 to < 4.5 g/dL<br>35 to < 45 g/L	7.0 to < 9.0 g/dL<br>70 to < 90 g/L<br>OR<br>Any decrease from Baseline<br>≥ 4.5 g/dL<br>≥ 45 g/L	< 7.0 g/dL<br>< 70 g/L
**Infant, 36–56 Days**<br>(HIV POSITIVE OR NEGATIVE)	8.5 to 9.4 g/dL<br>85 to 94 g/L	7.0 to < 8.5 g/dL<br>70 to < 85 g/L	6.0 to < 7.0 g/dL<br>60 to < 70 g/L	< 6.0 g/dL<br>< 60 g/L
**Infant, 22–35 Days**<br>(HIV POSITIVE OR NEGATIVE)	9.5 to 10.5 g/dL<br>95 to 105 g/L	8.0 to < 9.5 g/dL<br>80 to < 95 g/L	7.0 to < 8.0 g/dL<br>70 to < 80 g/L	< 7.0 g/dL<br>< 70 g/L
**Infant, 1–21 Days**<br>(HIV POSITIVE OR NEGATIVE)	12.0 to 13.0 g/dL<br>120 to 130 g/L	10.0 to < 12.0 g/dL<br>100 to < 120 g/L	9.0 to < 10.0 g/dL<br>90 to < 100 g/L	< 9.0 g/dL<br>< 90 g/L
Absolute Neutrophil Count (ANC)<br>**Adult and Pediatric,**<br>**≥ 7 Months#**	1000 to 1300/mm^3<br>1.00 to 1.30 GI/L	750 to < 1000/mm^3<br>0.75 to < 1.00 GI/L	500 to < 750/mm^3<br>0.50 to < 0.75 GI/L	< 500/mm^3<br>< 0.50 GI/L

HEMATOLOGY				
	**Grade 1**	**Grade 2**	**Grade 3**	**Grade 4**
Absolute CD4+ Count<br>HIV NEGATIVE ONLY<br>**Adult and Pediatric**<br>**> 13 Years**	300 to 400/mm^3<br>300 to 400/μL	200 to < 300/mm^3<br>200 to < 300/μL	100 to < 200/mm^3<br>100 to < 200/μL	< 100/mm^3<br>< 100/μL
Absolute Lymphocyte Count<br>HIV NEGATIVE ONLY<br>**Adult and Pediatric**<br>**> 13 Years**	600 to 650/mm^3<br>0.60 to 0.65 GI/L	500 to < 600/mm^3<br>0.50 to < 0.60 GI/L	350 to < 500/mm^3<br>0.35 to < 0.50 GI/L	< 350/mm^3<br>< 0.35 GI/L
Platelets	100,000 to < 125,000/mm^3<br>100 to < 125 GI/L	50,000 to < 100,000/mm^3<br>50 to < 100 GI/L	25,000 to < 50,000/mm^3<br>25 to < 50 GI/L	< 25,000/mm^3<br>< 25 GI/L
WBCs	2000/mm^3 to 2500/mm^3<br>2.00 GI/L to 2.50 GI/L	1,500 to < 2,000/mm^3<br>1.50 to < 2.00 GI/L	1000 to < 1,500/mm^3<br>1.00 to < 1.50 GI/L	< 1000/mm^3<br>< 1.00 GI/L
Hypofibrinogenemia	100 to 200 mg/dL<br>1.00 to 2.00 g/L	75 to < 100 mg/dL<br>0.75 to < 1.00 g/L	50 to < 75 mg/dL<br>0.50 to < 0.75 g/L	< 50 mg/dL<br>< 0.50 g/L
Hyperfibrinogenemia	> ULN to 600 mg/dL<br>> ULN to 6.0 g/L	> 600 mg/dL<br>> 6.0 g/L	—<br>—	—<br>—
Fibrin Split Product	20 to 40 μg/mL<br>20 to 40 mg/L	> 40 to 50 μg/mL<br>> 40 to 50 mg/L	> 50 to 60 μg/mL<br>> 50 to 60 mg/L	> 60 μg/mL<br>> 60 mg/L
Prothrombin Time (PT)	> 1.00 to 1.25 × ULN	> 1.25 to 1.50 × ULN	> 1.50 to 3.00 × ULN	> 3.00 × ULN
International Normalized Ratio of prothrombin time (INR)	1.1 to 1.5 x ULN	>1.5 to 2.0 x ULN	>2.0 to 3.0 x ULN	>3.0 x ULN

**HEMATOLOGY**				
	**Grade 1**	**Grade 2**	**Grade 3**	**Grade 4**
Activated Partial Thromboplastin Time (APTT)	> 1.00 to 1.66 × ULN	> 1.66 to 2.33 × ULN	> 2.33 to 3.00 × ULN	> 3.00 × ULN
Methemoglobin	5.0 to 10.0%	> 10.0 to 15.0%	> 15.0 to 20.0%	> 20.0%

An overlap between the Grade 1 scale and the Lab's normal range for absolute neutrophils may result for pediatric subjects. Please follow the Gilead convention of grading any result within the LLN and ULN a 0.

CHEMISTRY				
	**Grade 1**	**Grade 2**	**Grade 3**	**Grade 4**
Hyponatremia	130 to <LLN mEq/L<br>130 to <LLN mmol/L	125 to < 130 mEq/L<br>125 to < 130 mmol/L	121 to < 125 mEq/L<br>121 to < 125 mmol/L	< 121 mEq/L<br>< 121 mmol/L
Hypernatremia	>ULN to 150 mEq/L<br>>ULN to 150 mmol/L	> 150 to 154 mEq/L<br>> 150 to 154 mmol/L	> 154 to 159 mEq/L<br>> 154 to 159 mmol/L	> 159 mEq/L<br>> 159 mmol/L
Hypokalemia<br>**Adult and Pediatric ≥ 1 Year**	3.0 to <LLN mEq/L<br>3.0 to <LLN mmol/L	2.5 to < 3.0 mEq/L<br>2.5 to < 3.0 mmol/L	2.0 to < 2.5 mEq/L<br>2.0 to < 2.5 mmol/L	< 2.0 mEq/L<br>< 2.0 mmol/L
**Infant <1 Year**	3.0 to 3.4 mEq/L<br>3.0 to 3.4 mmol/L	2.5 to < 3.0 mEq/L<br>2.5 to <3.0 mmolL	2.0 to < 2.5 mEq/L<br>2.0 t o <2.5 mmolL	< 2.0 mEq/L<br><2.0 mmolL
Hyperkalemia<br>**Adult and Pediatric ≥ 1 Year**	5.6 to 6.0 mEq/L<br>5.6 to 6.0 mmol/L	> 6.0 to 6.5 mEq/L<br>> 6.0 to 6.5 mmol/L	> 6.5 to 7.0 mEq/L<br>> 6.5 to 7.0 mmol/L	> 7.0 mEq/L<br>> 7.0 mmol/L
**Infant <1 Year**	>ULN to 6.0 mEq/L<br>>ULN to 6.0 mmol/L	> 6.0 to 6.5 mEq/L<br>> 6.0 to 6.5 mmol/L	> 6.5 to 7.0 mEq/L<br>> 6.5 to 7.0 mmol/L	> 7.0 mEq/L<br>> 7.0 mmol/L
Hypoglycemia<br>**Adult and Pediatric ≥ 1 Month**	55 to 64 mg/dL<br>3.03 to 3.58 mmol/L	40 to < 55 mg/dL<br>2.20 to < 3.03 mmol/L	30 to < 40 mg/dL<br>1.64 to < 2.20 mmol/L	< 30 mg/dL<br>< 1.64 mmol/L
**Infant, < 1 Month**	50 to 54 mg/dL<br>2.8 to 3.0 mmol/L	40 to < 50 mg/dL<br>2.2 to < 2.8 mmol/L	30 to < 40 mg/dL<br>1.7 to < 2.2 mmol/L	< 30 mg/dL<br>< 1.7 mmol/L

CHEMISTRY				
	**Grade 1**	**Grade 2**	**Grade 3**	**Grade 4**
Hyperglycemia, Nonfasting	116 to 160 mg/dL<br>6.42 to 8.91 mmol/L	> 160 to 250 mg/dL<br>> 8.91 to 13.90 mmol/L	> 250 to 500 mg/dL<br>> 13.90 to 27.79 mmol/L	> 500 mg/dL<br>> 27.79 mmol/L
Hyperglycemia, Fasting	110 to 125 mg/dL<br>6.08 to 6.96 mmol/L	>125 to 250 mg/dL<br>>6.96 to 13.90 mmol/L	>250 to 500 mg/dL<br>>13.90 to 27.79 mmol/L	>500 mg/dL<br>>27.79 mmol/L
Hypocalcemia (corrected for albumin if appropriate*)<br>**Adult and Pediatric ≥2 Years**	7.8 <LLN mg/dL<br>1.94 to <LLN mmol/L	7.0 to < 7.8 mg/dL<br>1.74 to < 1.94 mmol/L	6.1 to < 7.0 mg/dL<br>1.51 to < 1.74 mmol/L	< 6.1 mg/dL<br>< 1.51 mmol/L
**Pediatric ≥7 days -2 Years**	7.8 to 8.4 mg/dL<br>1.94 to 2.10 mmol/L	7.0 to <7.8 mg/dL<br>1.74 to <1.94 mmolL	6.1 to <7.0 mg/dL<br>1.51 to < 1.74 mmolL	< 6.1 mg/dL<br>< 1.51 mmol/L
**Infant, < 7 Days**	6.5 to 7.5 mg/dL<br>1.61 to 1.88 mmol/L	6.0 to < 6.5 mg/dL<br>1.49 to < 1.61 mmol/L	5.5 to < 6.0 mg/dL<br>1.36 to < 1.49 mmol/L	< 5.5 mg/dL<br>< 1.36 mmol/L
Hypercalcemia (corrected for albumin if appropriate*)<br>**Adult and Pediatric ≥ 7 Days**	>ULN to 11.5 mg/dL<br>>ULN to 2.88 mmol/L	> 11.5 to 12.5 mg/dL<br>> 2.88 to 3.13 mmol/L	> 12.5 to 13.5 mg/dL<br>> 3.13 to 3.38 mmol/L	> 13.5 mg/dL<br>> 3.38 mmol/L
**Infant, < 7 Days**	11.5 to 12.4 mg/dL<br>2.86 to 3.10 mmol/L	> 12.4 to 12.9 mg/dL<br>> 3.10 to 3.23 mmol/L	> 12.9 to 13.5 mg/dL<br>> 3.23 to 3.38 mmol/L	> 13.5 mg/dL<br>> 3.38 mmol/L
Hypocalcemia (ionized)	3.0 mg/dL to < LLN<br>0.74 mmol/L to < LLN	2.5 to < 3.0 mg/dL<br>0.62 to < 0.74 mmol/L	2.0 to < 2.5 mg/dL<br>0.49 to < 0.62 mmol/L	< 2.0 mg/dL<br>< 0.49 mmol/L
Hypercalcemia (ionized)	> ULN to 6.0 mg/dL<br>> ULN to 1.50 mmol/L	> 6.0 to 6.5 mg/dL<br>> 1.50 to 1.63 mmol/L	> 6.5 to 7.0 mg/dL<br>> 1.63 to 1.75 mmol/L	> 7.0 mg/dL<br>> 1.75 mmol/L
Hypomagnesemia	1.40 to <LLN mg/dL<br>1.2 to <LLN mEq/L<br>0.58 to <LLN mmol/L	1.04 to < 1.40 mg/dL<br>0.9 to < 1.2 mEq/L<br>0.43 to < 0.58 mmol/L	0.67 to < 1.04 mg/dL<br>0.6 to < 0.9 mEq/L<br>0.28 to < 0.43 mmol/L	< 0.67 mg/dL<br>< 0.6 mEq/L<br>< 0.28 mmol/L

CHEMISTRY				
	**Grade 1**	**Grade 2**	**Grade 3**	**Grade 4**
Hypophosphatemia<br>**Adult and Pediatric**<br>**> 14 Years**	2.0 to < LLN mg/dL<br>0.63 to < LLN mmol/L	1.5 to < 2.0 mg/dL<br>0.47 to < 0.63 mmol/L	1.0 to < 1.5 mg/dL<br>0.31 to < 0.47 mmol/L	< 1.0 mg/dL<br>< 0.31 mmol/L
**Pediatric 1 Year–14 Years**	3.0 to <LLN mg/dL<br>0.96 to <LLN mmol/L	2.5 to < 3.0 mg/dL<br>0.80 to < 0.96 mmol/L	1.5 to < 2.5 mg/dL<br>0.47 to < 0.80 mmol/L	< 1.5 mg/dL<br>< 0.47 mmol/L
**Pediatric < 1 Year**	3.5 to <LLN mg/dL<br>1.12 to <LLN mmol/L	2.5 to < 3.5 mg/dL<br>0.80 to < 1.12 mmol/L	1.5 to < 2.5 mg/dL<br>0.47 to < 0.80 mmol/L	< 1.5 mg/dL<br>< 0.47 mmol/L
Hyperbilirubinemia<br>**Adult and Pediatric**<br>**> 14 Days**	> 1.0 to 1.5 × ULN	> 1.5 to 2.5 × ULN	> 2.5 to 5.0 × ULN	> 5.0 × ULN
**Infant, ≤ 14 Days**<br>(non-hemolytic)	NA	20.0 to 25.0 mg/dL<br>342 to 428 µmol/L	> 25.0 to 30.0 mg/dL<br>> 428 to 513 µmol/L	> 30.0 mg/dL<br>> 513 µmol/L
**Infant, ≤ 14 Days**<br>(hemolytic)	NA	NA	20.0 to 25.0 mg/dL<br>342 to 428 µmol/L	> 25.0 mg/dL<br>> 428 µmol/L
Blood Urea Nitrogen	1.25 to 2.50 × ULN	> 2.50 to 5.00 × ULN	> 5.00 to 10.00 × ULN	> 10.00 × ULN
Hyperuricemia	>ULN to 10.0 mg/dL<br>>ULN to 597 µmol/L	> 10.0 to 12.0 mg/dL<br>> 597 to 716 µmol/L	> 12.0 to 15.0 mg/dL<br>> 716 to 895 µmol/L	> 15.0 mg/dL<br>> 895 µmol/L

CHEMISTRY				
	**Grade 1**	**Grade 2**	**Grade 3**	**Grade 4**
Hypouricemia<br>**Adult and Pediatric**<br>**≥ 1 year**	1.5 mg/dL to < LLN<br>87 µmol/L to < LLN	1.0 to < 1.5 mg/dL<br>57 to < 87 µmol/L	0.5 to < 1.0 mg/dL<br>27 to < 57 µmol/L	< 0.5 mg/dL<br>< 27 µmol/L
**Infant < 1 Year**	N/A	1.0 mg/dl to <LLN-<br>57 µmol to <LLN	0.5 to < 1.0 mg/dL<br>27 to < 57 µmol/L	< 0.5 mg/dL<br>< 27 µmol/L
Creatinine**	> 1.50 to 2.00 mg/dL<br>> 133 to 177 µmol/L	> 2.00 to 3.00 mg/dL<br>> 177 to 265 µmol/L	> 3.00 to 6.00 mg/dL<br>> 265 to 530 µmol/L	> 6.00 mg/dL<br>> 530 µmol/L
Bicarbonate<br>**Adult and Pediatric**<br>**≥ 4 Years**	16.0 mEq/L to < LLN<br>16.0 mmol/L to < LLN	11.0 to < 16.0 mEq/L<br>11.0 to < 16.0 mmol/L	8.0 to < 11.0 mEq/L<br>8.0 to < 11.0 mmol/L	< 8.0 mEq/L<br>< 8.0 mmol/L
**Pediatric < 4 Years**	NA	11.0 mEq/Lto <LLN<br>11.0 mmol/L to <LLN	8.0 to < 11.0 mEq/L<br>8.0 to < 11.0 mmol/L	< 8.0 mEq/L<br>< 8.0 mmol/L
Triglycerides<br>(Fasting)	NA	500 to 750 mg/dL<br>5.64–8.47 mmol/L	> 750 to 1200 mg/dL<br>> 8.47–13.55 mmol/L	> 1200 mg/dL<br>> 13.55 mmol/L
LDL (Fasting)<br>Adult	130 to 160 mg/dL<br>3.35 to 4.15 mmol/L	>160 to 190 mg/dL<br>>4.15 to 4.92 mmol/L	> 190 mg/dL<br>>4.92 mmol/L	NA
LDL (Fasting)<br>Pediatric >2 to <18 years	110 to 130 mg/dL<br>2.84 to 3.37 mmol/L	>130 to 190 mg/dL<br>>3.37 to 4.92 mmol/L	> 190 mg/dL<br>>4.92 mmol/L	NA
Hypercholesterolemia<br>(Fasting)	200 to 239 mg/dL<br>5.16 to 6.19 mmol/L	> 239 to 300 mg/dL<br>> 6.19 to 7.77 mmol/L	> 300 mg/dL<br>> 7.77 mmol/L	NA
**Pediatric < 18 Years**	170 to 199 mg/dL<br>4.39 to 5.15 mmol/L	> 199 to 300 mg/dL<br>> 5.15 to 7.77 mmol/L	> 300 mg/dL<br>> 7.77 mmol/L	NA
Creatine Kinase	3.0 to < 6.0 × ULN	6.0 to < 10.0 × ULN	10.0 to < 20.0 × ULN	≥ 20.0 × ULN

* Calcium should be corrected for albumin if albumin is < 4.0 g/dL

** An overlap between the Grade 1 scale and the Lab's normal range for creatinine may result for Male subjects >70 yrs. Please follow the Gilead convention of grading any result within the LLN and ULN a 0.

ENZYMES				
	**Grade 1**	**Grade 2**	**Grade 3**	**Grade 4**
AST (SGOT)	1.25 to 2.50 × ULN	> 2.50 to 5.00 × ULN	> 5.00 to 10.00 × ULN	> 10.00 × ULN
ALT (SGPT)	1.25 to 2.50 × ULN	> 2.50 to 5.00 × ULN	> 5.00 to 10.00 × ULN	> 10.00 × ULN
GGT	1.25 to 2.50 × ULN	> 2.50 to 5.00 × ULN	> 5.00 to 10.00 × ULN	> 10.00 × ULN
Alkaline Phosphatase	1.25 to 2.50 × ULN	> 2.50 to 5.00 × ULN	> 5.00 to 10.00 × ULN	> 10.00 × ULN
Total Amylase	> 1.0 to 1.5 × ULN	> 1.5 to 2.0 × ULN	> 2.0 to 5.0 × ULN	> 5.0 × ULN
Pancreatic Amylase	> 1.0 to 1.5 × ULN	> 1.5 to 2.0 × ULN	> 2.0 to 5.0 × ULN	> 5.0 × ULN
Lipase	> 1.0 to 1.5 × ULN	> 1.5 to 3.0 × ULN	> 3.0 to 5.0 × ULN	> 5.0 × ULN
Albumin<br>**Pediatrics <16 years**	-	2.0 to < LLN g/dL<br>20 to < LLN g/L	< 2.0 g/dL<br>< 20 g/L	NA
**≥ 16 years**	3.0 g/dL to < LLN<br>30 g/L to < LLN	2.0 to < 3.0 g/dL<br>20 to < 30 g/L	< 2.0 g/dL<br>< 20 g/L	NA

URINALYSIS

	**Grade 1**	**Grade 2**	**Grade 3**	**Grade 4**
Hematuria (Dipstick)	1+	2+	3-4+	NA
Hematuria (Quantitative)<br>See Note below<br>Females	>ULN - 10 RBC/HPF	> 10-75 RBC/HPF	> 75 RBC/HPF	NA
Males	6-10 RBC/HPF	> 10-75 RBC/HPF	> 75 RBC/HPF	NA
Proteinuria (Dipstick)	1+	2–3+	4+	NA
Proteinuria, 24 Hour Collection				
**Adult and Pediatric ≥ 10 Years**	200 to 999 mg/24 h	>999 to 1999 mg/24 h	>1999 to 3500 mg/24 h	> 3500 mg/24 h
**Pediatric > 3 Mo to < 10 Years**	201 to 499 mg/m^2/24 h	>499 to 799 mg/m^2/24 h	>799 to 1000 mg/m^2/24 h	> 1000 mg/ m^2/24 h
Glycosuria (Dipstick)	1+	2-3+	4+	NA

Notes:

- Toxicity grades for Quantitative and Dipstick Hematuria will be assigned by Covance Laboratory, however for other laboratories, toxicity grades will only be assigned to Dipstick Hematuria.
- With the exception of lipid tests, any graded laboratory test with a result that is between the LLN and ULN should be assigned Grade 0.
- If the severity of a clinical AE could fall under either one of two grades (eg, the severity of an AE could be either Grade 2 or Grade 3), select the higher of the two grades for the AE.

CARDIOVASCULAR				
	**Grade 1**	**Grade 2**	**Grade 3**	**Grade 4**
Cardiac Arrhythmia (general)<br>(By ECG or physical exam)	Asymptomatic AND No intervention indicated	Asymptomatic AND Non-urgent medical intervention indicated	Symptomatic, non-life-threatening AND Non-urgent medical intervention indicated	Life-threatening arrhythmia OR Urgent intervention indicated
Cardiac-ischemia/Infarction	NA	NA	Symptomatic ischemia (stable angina) OR Testing consistent with ischemia	Unstable angina OR Acute myocardial infarction
Hemorrhage (significant acute blood loss)	NA	Symptomatic AND No transfusion indicated	Symptomatic AND Transfusion of ≤ 2 units packed RBCs (for children ≤ 10 cc/kg) indicated	Life-threatening hypotension OR Transfusion of > 2 units packed RBCs indicated (for children ≤ 10 cc/kg) indicated
Hypertension (with repeat testing at same visit)<br><br>**Pediatric ≤ 17 Years** (with repeat testing at same visit)	140–159 mmHg systolic<br>OR<br>90–99 mmHg diastolic<br><br>NA	> 159–179 mmHg systolic<br>OR<br>> 99–109 mmHg diastolic<br><br>91st–94th percentile adjusted for age, height, and gender (systolic and/or diastolic)	> 179 mmHg systolic<br>OR<br>> 109 mmHg diastolic<br><br>≥ 95th percentile adjusted for age, height, and gender (systolic and/or diastolic)	Life-threatening consequences (eg, malignant hypertension) OR Hospitalization (other than ER visit) indicated<br><br>Life-threatening consequences (eg, malignant hypertension) OR Hospitalization indicated (other than emergency room visit)
Hypotension	NA	Symptomatic, corrected with oral fluid replacement	Symptomatic, IV fluids indicated	Shock requiring use of vasopressors or mechanical assistance to maintain blood pressure
Pericardial Effusion	Asymptomatic, small effusion requiring no intervention	Asymptomatic, moderate or larger effusion requiring no intervention	Effusion with non-life-threatening physiologic consequences OR Effusion with nonurgent intervention indicated	Life-threatening consequences (eg, tamponade) OR Urgent intervention indicated
Prolonged PR Interval	PR interval<br>0.21 to 0.25 sec	PR interval<br>> 0.25 sec	Type II 2nd degree AV block OR Ventricular pause > 3.0 sec	Complete AV block

CARDIOVASCULAR				
	**Grade 1**	**Grade 2**	**Grade 3**	**Grade 4**
**Pediatric ≤ 16 Years**	1st degree AV block (PR > normal for age and rate)	Type I 2nd degree AV block	Type II 2nd degree AV block	Complete AV block
Prolonged QTc	Asymptomatic, QTc interval 0.45 to 0.47 sec OR Increase interval < 0.03 sec above baseline	Asymptomatic, QTc interval 0.48 to 0.49 sec OR Increase in interval 0.03 to 0.05 sec above baseline	Asymptomatic, QTc interval ≥ 0.50 sec OR Increase in interval ≥ 0.06 sec above baseline	Life-threatening consequences, eg, Torsade de pointes or other associated serious ventricular dysrhythmia
**Pediatric ≤ 16 Years**	Asymptomatic, QTc interval 0.450 to 0.464 sec	Asymptomatic, QTc interval 0.465 to 0.479 sec	Asymptomatic, QTc interval ≥ 0.480 sec	Life-threatening consequences, eg, Torsade de pointes or other associated serious ventricular dysrhythmia
Thrombosis/Embolism	NA	Deep vein thrombosis AND No intervention indicated (eg, anticoagulation, lysis filter, invasive procedure)	Deep vein thrombosis AND Intervention indicated (eg, anticoagulation, lysis filter, invasive procedure)	Embolic event (eg, pulmonary embolism, life-threatening thrombus)
Vasovagal Episode (associated with a procedure of any kind)	Present without loss of consciousness	Present with transient loss of consciousness	NA	NA
Ventricular Dysfunction (congestive heart failure, CHF)	NA	Asymptomatic diagnostic finding AND intervention indicated	New onset with symptoms OR Worsening symptomatic CHF	Life-threatening CHF

RESPIRATORY				
	**Grade 1**	**Grade 2**	**Grade 3**	**Grade 4**
Bronchospasm (acute)	FEV1 or peak flow reduced to 70% to 80%	FEV1 or peak flow 50% to 69%	FEV1 or peak flow 25% to 49%	Cyanosis OR FEV1 or peak flow < 25% OR Intubation
Dyspnea or Respiratory Distress	Dyspnea on exertion with no or minimal interference with usual social & functional activities	Dyspnea on exertion causing greater than minimal interference with usual social & functional activities	Dyspnea at rest causing inability to perform usual social & functional activities	Respiratory failure with ventilatory support indicated
**Pediatric < 14 Years**	Wheezing OR minimal increase in respiratory rate for age	Nasal flaring OR Intercostal retractions OR Pulse oximetry 90% to 95%	Dyspnea at rest causing inability to perform usual social & functional activities OR Pulse oximetry < 90%	Respiratory failure with ventilatory support indicated

OCULAR/VISUAL				
	**Grade 1**	**Grade 2**	**Grade 3**	**Grade 4**
Uveitis	Asymptomatic but detectable on exam	Symptomatic anterior uveitis OR Medical intervention indicated	Posterior or pan-uveitis OR Operative intervention indicated	Disabling visual loss in affected eye(s)
Visual Changes (from baseline)	Visual changes causing no or minimal interference with usual social & functional activities	Visual changes causing greater than minimal interference with usual social & functional activities	Visual changes causing inability to perform usual social & functional activities	Disabling visual loss in affected eye(s)

SKIN				
	**Grade 1**	**Grade 2**	**Grade 3**	**Grade 4**
Alopecia	Thinning detectable by study participant or caregiver (for disabled adults)	Thinning or patchy hair loss detectable by health care provider	Complete hair loss	NA
Cutaneous Reaction – Rash	Localized macular rash	Diffuse macular, maculopapular, or morbilliform rash OR Target lesions	Diffuse macular, maculopapular, or morbilliform rash with vesicles or limited number of bullae OR Superficial ulcerations of mucous membrane limited to one site	Extensive or generalized bullous lesions OR Stevens-Johnson syndrome OR Ulceration of mucous membrane involving two or more distinct mucosal sites OR Toxic epidermal necrolysis (TEN)
Hyperpigmentation	Slight or localized	Marked or generalized	NA	NA
Hypopigmentation	Slight or localized	Marked or generalized	NA	NA
Pruritis (itching – no skin lesions)<br>(See also Injection Site Reactions: Pruritis associated with injection)	Itching causing no or minimal interference with usual social & functional activities	Itching causing greater than minimal interference with usual social & functional activities	Itching causing inability to perform usual social & functional activities	NA

GASTROINTESTINAL				
	**Grade 1**	**Grade 2**	**Grade 3**	**Grade 4**
Anorexia	Loss of appetite without decreased oral intake	Loss of appetite associated with decreased oral intake without significant weight loss	Loss of appetite associated with significant weight loss	Life-threatening consequences OR Aggressive intervention indicated [eg, tube feeding or total parenteral nutrition]
Ascites	Asymptomatic	Symptomatic AND Intervention indicated (eg, diuretics or therapeutic paracentesis)	Symptomatic despite intervention	Life-threatening consequences
Cholecystitis	NA	Symptomatic AND Medical intervention indicated	Radiologic, endoscopic, or operative intervention indicated	Life-threatening consequences (eg, sepsis or perforation)
Constipation	NA	Persistent constipation requiring regular use of dietary modifications, laxatives, or enemas	Obstipation with manual evacuation indicated	Life-threatening consequences (eg, obstruction)
Diarrhea				
**Adult and Pediatric ≥ 1 Year**	Transient or intermittent episodes of unformed stools OR Increase of ≤ 3 stools over baseline/24 hr	Persistent episodes of unformed to watery stools OR Increase of 4–6 stools over baseline per 24 hrs.	Bloody diarrhea OR Increase of ≥ 7 stools per 24-hour period OR IV fluid replacement indicated	Life-threatening consequences (eg, hypotensive shock)
**Pediatric < 1 Year**	Liquid stools (more unformed than usual) but usual number of stools	Liquid stools with increased number of stools OR Mild dehydration	Liquid stools with moderate dehydration	Liquid stools resulting in severe dehydration with aggressive rehydration indicated OR Hypotensive shock
Dysphagia-Odynophagia	Symptomatic but able to eat usual diet	Symptoms causing altered dietary intake without medical intervention indicated	Symptoms causing severely altered dietary intake with medical intervention indicated	Life-threatening reduction in oral intake

GASTROINTESTINAL				
	**Grade 1**	**Grade 2**	**Grade 3**	**Grade 4**
Mucositis/Stomatitis (clinical exam)<br>See also Proctitis, Dysphagia-Odynophagia	Erythema of the mucosa	Patchy pseudomembranes or ulcerations	Confluent pseudomembranes or ulcerations OR Mucosal bleeding with minor trauma	Tissue necrosis OR Diffuse spontaneous mucosal bleeding OR Life-threatening consequences (eg, aspiration, choking)
Nausea	Transient (< 24 hours) or intermittent nausea with no or minimal interference with oral intake	Persistent nausea resulting in decreased oral intake for 24–48 hours	Persistent nausea resulting in minimal oral intake for > 48 hours OR Aggressive rehydration indicated (eg, IV fluids)	Life-threatening consequences (eg, hypotensive shock)
Pancreatitis	NA	Symptomatic AND Hospitalization not indicated (other than ER visit)	Symptomatic AND Hospitalization indicated (other than ER visit)	Life-threatening consequences (eg, sepsis, circulatory failure, hemorrhage)
Proctitis (functional-symptomatic)<br>Also see Mucositis/ Stomatitis for Clinical Exam	Rectal discomfort AND No intervention indicated	Symptoms causing greater than minimal interference with usual social & functional activities OR Medical intervention indicated	Symptoms causing inability to perform usual social/ functional activities OR Operative intervention indicated	Life-threatening consequences (eg, perforation)
Vomiting	Transient or intermittent vomiting with no or minimal interference with oral intake	Frequent episodes of vomiting with no or mild dehydration	Persistent vomiting resulting in orthostatic hypotension OR Aggressive rehydration indicated	Life-threatening consequences (eg, hypotensive shock)

NEUROLOGICAL				
	**Grade 1**	**Grade 2**	**Grade 3**	**Grade 4**
Alteration in Personality-Behavior or in Mood (eg, agitation, anxiety, depression, mania, psychosis)	Alteration causing no or minimal interference with usual social & functional activities	Alteration causing greater than minimal interference with usual social & functional activities	Alteration causing inability to perform usual social & functional activities	Behavior potentially harmful to self or others (eg, suicidal/homicidal ideation or attempt, acute psychosis) OR Causing inability to perform basic self-care functions
Altered Mental Status<br>For Dementia, see Cognitive and Behavioral/Attentional Disturbance (including dementia and ADD)	Changes causing no or minimal interference with usual social & functional activities	Mild lethargy or somnolence causing greater than minimal interference with usual social & functional activities	Confusion, memory impairment, lethargy, or somnolence causing inability to perform usual social & functional activities	Delirium OR obtundation, OR coma
Ataxia	Asymptomatic ataxia detectable on exam OR Minimal ataxia causing no or minimal interference with usual social & functional activities	Symptomatic ataxia causing greater than minimal interference with usual social & functional activities	Symptomatic ataxia causing inability to perform usual social & functional activities	Disabling ataxia causing inability to perform basic self-care functions
Cognitive and Behavioral/Attentional Disturbance (including dementia and Attention Deficit Disorder)	Disability causing no or minimal interference with usual social & functional activities OR Specialized resources not indicated	Disability causing greater than minimal interference with usual social & functional activities OR Specialized resources on part-time basis indicated	Disability causing inability to perform usual social & functional activities OR Specialized resources on a full-time basis indicated	Disability causing inability to perform basic self-care functions OR Institutionalization indicated
CNS Ischemia (acute)	NA	NA	Transient ischemic attack	Cerebral vascular accident (CVA, stroke) with neurological deficit

NEUROLOGICAL				
	**Grade 1**	**Grade 2**	**Grade 3**	**Grade 4**
Developmental delay – **Pediatric ≤ 16 Years**	Mild developmental delay, either motor or cognitive, as determined by comparison with a developmental screening tool appropriate for the setting	Moderate developmental delay, either motor or cognitive, as determined by comparison with a developmental screening tool appropriate for the setting	Severe developmental delay, either motor or cognitive, as determined by comparison with a developmental screening tool appropriate for the setting	Developmental regression, either motor or cognitive, as determined by comparison with a developmental screening tool appropriate for the setting
Headache	Symptoms causing no or minimal interference with usual social & functional activities	Symptoms causing greater than minimal interference with usual social & functional activities	Symptoms causing inability to perform usual social & functional activities	Symptoms causing inability to perform basic self-care functions OR Hospitalization indicated (other than ER visit) OR Headache with significant impairment of alertness or other neurologic function
Insomnia	NA	Difficulty sleeping causing greater than minimal interference with usual social/functional activities	Difficulty sleeping causing inability to perform usual social & functional activities	Disabling insomnia causing inability to perform basic self-care functions
Neuromuscular Weakness (including myopathy & neuropathy)	Asymptomatic with decreased strength on exam OR Minimal muscle weakness causing no or minimal interference with usual social & functional activities	Muscle weakness causing greater than minimal interference with usual social & functional activities	Muscle weakness causing inability to perform usual social & functional activities	Disabling muscle weakness causing inability to perform basic self-care functions OR Respiratory muscle weakness impairing ventilation
Neurosensory Alteration (including paresthesia and painful neuropathy)	Asymptomatic with sensory alteration on exam or minimal paresthesia causing no or minimal interference with usual social & functional activities	Sensory alteration or paresthesia causing greater than minimal interference with usual social & functional activities	Sensory alteration or paresthesia causing inability to perform usual social & functional activities	Disabling sensory alteration or paresthesia causing inability to perform basic self-care functions

NEUROLOGICAL				
	**Grade 1**	**Grade 2**	**Grade 3**	**Grade 4**
Seizure: (new onset)	NA	1 seizure	2–4 seizures	Seizures of any kind that are prolonged, repetitive (eg, status epilepticus), or difficult to control (eg, refractory epilepsy)
Seizure: (pre-existing)<br>For Worsening of Existing Epilepsy the Grades Should Be Based on an Increase from Previous Level of Control to Any of These Levels	NA	Increased frequency of pre-existing seizures (non-repetitive) without change in seizure character OR infrequent breakthrough seizures while on stable meds in a previously controlled seizure disorder	Change in seizure character from baseline either in duration or quality (eg, severity or focality)	Seizures of any kind that are prolonged, repetitive (eg, status epilepticus), or difficult to control (eg, refractory epilepsy)
Seizure<br>**– Pediatric < 18 Years**	Seizure, generalized onset with or without secondary generalization, lasting < 5 minutes with < 24 hours post ictal state	Seizure, generalized onset with or without secondary generalization, lasting 5–20 minutes with < 24 hours post ictal state	Seizure, generalized onset with or without secondary generalization, lasting > 20 minutes	Seizure, generalized onset with or without secondary generalization, requiring intubation and sedation
Syncope (not associated with a procedure)	NA	Present	NA	NA
Vertigo	Vertigo causing no or minimal interference with usual social & functional activities	Vertigo causing greater than minimal interference with usual social & functional activities	Vertigo causing inability to perform usual social & functional activities	Disabling vertigo causing inability to perform basic self-care functions

MUSCULOSKELETAL				
	**Grade 1**	**Grade 2**	**Grade 3**	**Grade 4**
Arthralgia<br>See also Arthritis	Joint pain causing no or minimal interference with usual social & functional activities	Joint pain causing greater than minimal interference with usual social & functional activities	Joint pain causing inability to perform usual social & functional activities	Disabling joint pain causing inability to perform basic self-care functions
Arthritis<br>See also Arthralgia	Stiffness or joint swelling causing no or minimal interference with usual social & functional activities	Stiffness or joint swelling causing greater than minimal interference with usual social & functional activities	Stiffness or joint swelling causing inability to perform usual social & functional activities	Disabling joint stiffness or swelling causing inability to perform basic self-care functions
Bone Mineral Loss	BMD t-score or z-score –2.5 to –1.0	BMD t-score or z-score < –2.5	Pathological fracture (including loss of vertebral height)	Pathologic fracture causing life-threatening consequences
**Pediatric < 21 Years**	BMD z-score –2.5 to –1.0	BMD z-score < –2.5	Pathological fracture (including loss of vertebral height)	Pathologic fracture causing life-threatening consequences
Myalgia<br>(non-injection site)	Muscle pain causing no or minimal interference with usual social & functional activities	Muscle pain causing greater than minimal interference with usual social & functional activities	Muscle pain causing inability to perform usual social & functional activities	Disabling muscle pain causing inability to perform basic self-care functions
Osteonecrosis	NA	Asymptomatic with radiographic findings AND No operative intervention indicated	Symptomatic bone pain with radiographic findings OR Operative intervention indicated	Disabling bone pain with radiographic findings causing inability to perform basic self-care functions

**SYSTEMIC**				
	**Grade 1**	**Grade 2**	**Grade 3**	**Grade 4**
Acute Systemic Allergic Reaction	Localized urticaria (wheals) with no medical intervention indicated	Localized urticaria with medical intervention indicated OR Mild angioedema with no medical intervention indicated	Generalized urticaria OR Angioedema with medical intervention indicated OR Symptomatic mild bronchospasm	Acute anaphylaxis OR Life-threatening bronchospasm OR laryngeal edema
Chills	Symptoms causing no or minimal interference with usual social & functional activities	Symptoms causing greater than minimal interference with usual social & functional activities	Symptoms causing inability to perform usual social & functional activities	NA
Fatigue<br>Malaise	Symptoms causing no or minimal interference with usual social & functional activities	Symptoms causing greater than minimal interference with usual social & functional activities	Symptoms causing inability to perform usual social & functional activities	Incapacitating fatigue/malaise symptoms causing inability to perform basic self-care functions
Fever (nonaxillary)	37.7°C to 38.6°C<br>99.8°F to 101.5°F	38.7°C to 39.3°C<br>101.6°F to 102.8°F	39.4°C to 40.5°C<br>102.9°F to 104.9°F	> 40.5°C<br>> 104.9°F
Pain- Indicate Body Site<br>See also Injection Site Pain, Headache, Arthralgia, and Myalgia	Pain causing no or minimal interference with usual social & functional activities	Pain causing greater than minimal interference with usual social & functional activities	Pain causing inability to perform usual social & functional activities	Disabling pain causing inability to perform basic self-care functions OR Hospitalization (other than ER visit) indicated
Unintentional Weight Loss	NA	5% to 9% loss in body weight from baseline	10% to 19% loss in body weight from baseline	≥ 20% loss in body weight from baseline OR Aggressive intervention indicated [eg, tube feeding or total parenteral nutrition]

**INJECTION SITE REACTION**				
	**Grade 1**	**Grade 2**	**Grade 3**	**Grade 4**
Injection Site Pain (pain without touching)<br>Or Tenderness (pain when area is touched)	Pain/tenderness causing no or minimal limitation of use of limb	Pain/tenderness limiting use of limb OR Pain/tenderness causing greater than minimal interference with usual social & functional activities	Pain/tenderness causing inability to perform usual social & functional activities	Pain/tenderness causing inability to perform basic self-care function OR Hospitalization (other than ER visit) indicated for management of pain/tenderness
Injection Site Reaction (Localized), > 15 Years<br><br>**Pediatric ≤ 15 Years**	Erythema OR Induration of 5 × 5 cm to 9 × 9 cm (or 25–81 × cm^2)<br><br>**Erythema OR Induration OR Edema present but ≤ 2.5 cm diameter**	Erythema OR Induration OR Edema > 9 cm any diameter (or > 81 cm^2)<br><br>**Erythema OR Induration OR Edema > 2.5 cm diameter but < 50% surface area of the extremity segment (eg, upper arm/thigh)**	Ulceration OR Secondary infection OR Phlebitis OR Sterile abscess OR Drainage<br><br>**Erythema OR Induration OR Edema involving ≥ 50% surface area of the extremity segment (eg, upper arm/thigh) OR Ulceration OR Secondary infection OR Phlebitis OR Sterile abscess OR Drainage**	Necrosis (involving dermis and deeper tissue)<br><br>**Necrosis (involving dermis and deeper tissue)**
Pruritis Associated with Injection<br>See also Skin: Pruritis (itching—no skin lesions)	Itching localized to injection site AND Relieved spontaneously or with < 48 h treatment	Itching beyond the injection site but not generalized OR Itching localized to injection site requiring ≥ 48 h treatment	Generalized itching causing inability to perform usual social & functional activities	NA

**ENDOCRINE/METABOLIC**				
	**Grade 1**	**Grade 2**	**Grade 3**	**Grade 4**
Lipodystrophy (eg, back of neck, breasts, abdomen)	Detectable by study participant or caregiver (for young children and disabled adults)	Detectable on physical exam by health care provider	Disfiguring OR Obvious changes on casual visual inspection	NA
Diabetes Mellitus	NA	New onset without need to initiate medication OR Modification of current meds to regain glucose control	New onset with initiation of indicated med OR Diabetes uncontrolled despite treatment modification	Life-threatening consequences (eg, ketoacidosis, hyperosmolar non-ketotic coma)
Gynecomastia	Detectable by study participant or caregiver (for young children and disabled adults)	Detectable on physical exam by health care provider	Disfiguring OR Obvious on casual visual inspection	NA
Hyperthyroidism	Asymptomatic	Symptomatic causing greater than minimal interference with usual social & functional activities OR Thyroid suppression therapy indicated	Symptoms causing inability to perform usual social & functional activities OR Uncontrolled despite treatment modification	Life-threatening consequences (eg, thyroid storm)
Hypothyroidism	Asymptomatic	Symptomatic causing greater than minimal interference with usual social & functional activities OR Thyroid replacement therapy indicated	Symptoms causing inability to perform usual social & functional activities OR Uncontrolled despite treatment modification	Life-threatening consequences (eg, myxedema coma)
Lipoatrophy (eg, fat loss from the face, extremities, buttocks)	Detectable by study participant or caregiver (for young children and disabled adults)	Detectable on physical exam by health care provider	Disfiguring OR Obvious on casual visual inspection	NA

GENITOURINARY				
	**Grade 1**	**Grade 2**	**Grade 3**	**Grade 4**
Intermenstrual Bleeding (IMB)	Spotting observed by participant OR Minimal blood observed during clinical or colposcopic exam	Intermenstrual bleeding not greater in duration or amount than usual menstrual cycle	Intermenstrual bleeding greater in duration or amount than usual menstrual cycle	Hemorrhage with life-threatening hypotension OR Operative intervention indicated
Urinary Tract obstruction (eg, stone)	NA	Signs or symptoms of urinary tract obstruction without hydronephrosis or renal dysfunction	Signs or symptoms of urinary tract obstruction with hydronephrosis or renal dysfunction	Obstruction causing life-threatening consequences

INFECTION				
	**Grade 1**	**Grade 2**	**Grade 3**	**Grade 4**
Infection (any other than HIV infection)	Localized, no systemic antiμbial treatment indicated AND Symptoms causing no or minimal interference with usual social & functional activities	Systemic antiμbial treatment indicated OR Symptoms causing greater than minimal interference with usual social & functional activities	Systemic antiμbial treatment indicated AND Symptoms causing inability to perform usual social & functional activities OR Operative intervention (other than simple incision and drainage) indicated	Life-threatening consequences (eg, septic shock)

Basic Self-care Functions: Activities such as bathing, dressing, toileting, transfer/movement, continence, and feeding.
Usual Social & Functional Activities: Adaptive tasks and desirable activities, such as going to work, shopping, cooking, use of transportation, pursuing a hobby, etc.

Appendix 5. Pregnancy Precautions, Definition for Female of Childbearing Potential, and Contraceptive Requirements

1) Definitions

a. Definition of Childbearing Potential

For the purposes of this study, a female born subject is considered of childbearing potential following the initiation of puberty (Tanner stage 2) until becoming post-menopausal, unless permanently sterile or with medically documented ovarian failure.

Women are considered to be in a postmenopausal state when they are ≥ 54 years of age with cessation of previously occurring menses for ≥ 12 months without an alternative cause.

Permanent sterilization includes hysterectomy, bilateral oophorectomy, or bilateral salpingectomy in a female subject of any age.

Women who are not post-menopausal or permanently sterile in accordance with these definitions will be considered of childbearing potential.

b. Definition of Male Fertility

For the purposes of this study, a male born subject is considered fertile after the initiation of puberty unless permanently sterile by bilateral orchidectomy or medical documentation.

2) Contraception Requirements for Female Subjects

a. Study Drug Effects on Pregnancy and Hormonal Contraception

Data from clinical pharmacokinetic interaction studies of E/C/F/TAF have demonstrated that E/C/F/TAF reduces the exposure of ethinyl estradiol in hormonal contraceptives. No clinically significant interactions between the components of F/R/TAF and LDV/SOF and hormonal contraception are known. Female subjects who choose hormonal contraception will be required to select a second backup method as defined below.

Non-clinical toxicity studies of LDF/SOF, F/R/TAF, and E/C/F/TAF have demonstrated no adverse effect on fertility or embryo-fetal development. However, there is insufficient clinical data available on use of these products in pregnant women. Please refer to the latest version of the IB (F/R/TAF) and package inserts (E/C/F/TAF and LDV/SOF) for additional information.

b. Contraceptive Methods allowed for Female Subjects of Childbearing Potential

The inclusion of female subjects of childbearing potential requires using at least an *acceptable effective* contraceptive measure. Women must also not rely solely on hormone-containing contraceptives (except for intrauterine devices [IUDs] that may release hormones) as a form of birth control during the study. Women may continue oral, injectable, transdermal, or other hormonally based contraceptives that are designed to inhibit ovulation, if desired, but these will

not be acceptable as a sole form of contraception; an additional form of acceptable effective contraception (methods below) must be chosen even if hormonal ovulation-inhibiting contraception is continued.

Women of childbearing potential must have a negative serum pregnancy test at Screening and a negative pregnancy test on the Day 1 visit prior to randomization. At minimum, a pregnancy test will be performed at the end of relevant systemic exposure. In the event of a delayed menstrual period (over one month between menstruations), a pregnancy test must be performed to rule out pregnancy. This is even true for women of childbearing potential with infrequent or irregular periods.

Women of child bearing potential in this study must agree to use the following forms of contraception from Screening until 30 days after the last dose of study drug.

- Complete abstinence from intercourse of reproductive potential. Abstinence is an acceptable method of contraception only when it is in line with the subject's preferred and usual lifestyle.

Or

- Consistent and correct use of 1 of the following methods of birth control listed below.
  - Intrauterine device (IUD) with a failure rate of < 1% per year. Hormonal IUDs meeting this criterion are acceptable as a sole method.
  - Tubal sterilization
  - Essure micro-insert system (provided confirmation of success 3 months after procedure)
  - Vasectomy in the male partner (provided that the partner is the sole sexual partner and had confirmation of surgical success 3 months after procedure)
  - Combined barrier methods:
    - Diaphragm with spermicide and a male condom without spermicide

      Or

    - Cervical cap with spermicide and male condom without spermicide
- Women who choose to continue on their hormonal contraceptive method (eg, combined oral contraceptives, injectable, transdermal, and intravaginal contraceptives) are asked to also choose one of the above methods to supplement the hormonally based method given the effect of E/C/F/TAF on ethinyl estradiol. These women *may*, however, use their hormonal contraceptive method and choose a single barrier method to supplement their hormonal contraceptive method (preferably the male condom).

Female subjects must also refrain from egg donation and in vitro fertilization during treatment and until at least 30 days after the last dose of study drug.

3) Contraception Requirements for Male Subjects

During the study and for 30 days after the last dose of study drug, male subjects with female partners of childbearing potential should use condoms without spermicide when engaging in intercourse of reproductive potential.

4) Unacceptable Birth Control Methods

Birth control methods that are unacceptable include periodic abstinence (eg, calendar, ovulation, symptothermal, post-ovulation methods), withdrawal (coitus interruptus), spermicides only, and lactational amenorrhea method (LAM). Female condom and male condom should not be used together.

5) Procedures to be Followed in the Event of Pregnancy

Subjects will be instructed to notify the investigator if they become pregnant at any time during the study, or if they become pregnant within 30 days of last study drug dose. Subjects who become pregnant or who suspect that they are pregnant during the study must report the information to the investigator and discontinue study drug immediately. Male subjects should also notify the investigator if their female partner becomes pregnant during the study or within 30 days of last dose of study drugs. Instructions for reporting pregnancy and pregnancy outcome are outlined in Section 7.7.2.1.

Appendix 6. CDC Classification Systems, AIDS-Indicator Conditions

- Bacterial infections, multiple or recurrent
- Candidiasis of bronchi, trachea, or lungs
- Candidiasis of esophagus
- Cervical cancer, invasive
- Coccidioidomycosis, disseminated or extrapulmonary
- Cryptococcosis, extrapulmonary
- Cryptosporidiosis, chronic intestinal (>1 month's duration)
- Cytomegalovirus disease (other than liver, spleen, or nodes)
- Cytomegalovirus retinitis (with loss of vision)
- Encephalopathy attributed to HIV
- Herpes simplex: chronic ulcers (>1 month's duration) or bronchitis, pneumonitis, or esophagitis
- Histoplasmosis, disseminated or extrapulmonary
- Isosporiasis, chronic intestinal (>1 month duration)
- Kaposi sarcoma
- Lymphoma, Burkitt (or equivalent term)
- Lymphoma, immunoblastic (or equivalent term)
- Lymphoma, primary, of brain
- *Mycobacterium avium* complex or *Mycobacterium kansasii*, disseminated or extrapulmonary
- *Mycobacterium tuberculosis* of any site, pulmonary, disseminated, or extrapulmonary
- *Mycobacterium*, other species or unidentified species, disseminated or extrapulmonary
- *Pneumocystis jirovecii* (previously known as "*Pneumocystis carinii*") pneumonia
- Pneumonia, recurrent

- Progressive multifocal leukoencephalopathy
- *Salmonella* septicemia, recurrent
- Toxoplasmosis of brain
- Wasting syndrome attributed to HIV Infection

CDC Revised Surveillance Case Definition for HIV Infection – 2014 {Selik et al 2014}
